# Apc-deficient pituitary stem cells drive adamantinomatous craniopharyngioma through senescence-associated inflammation

**DOI:** 10.1038/s44321-026-00525-2

**Published:** 2026-10-09

**Authors:** James Blackburn, James G Nicholson, Laura Gomez-Corral, Jingyi Xue, Ashutosh Rai, Bryan Padraig Finn, Rachael J L Tan, Kalyana C Chakravarthi, Angelica Gualtieri, Charlotte L Hall, Gabor Czibik, Pinaki Dutta, Márta Korbonits, Federico Roncaroli, Shannon W Davis, Carles Gaston-Massuet

**Affiliations:** 1 Centre for Endocrinology, William Harvey Research Institute, Barts & the London School of Medicine and Dentistry, Queen Mary University of London, London, UK; 2 Department of Biochemistry, Panjab University, Chandigarh, India; 3 Department of Endocrinology, Postgraduate Institute of Medical Education and Research, Chandigarh, India; 4 Geoffrey Jefferson Brain Research Centre, Division of Neuroscience, Faculty of Biology, Medicine and Health, University of Manchester, Manchester, UK; 5 Department of Biological Sciences, University of South Carolina, Columbia, SC USA

**Keywords:** Cancer, Genetics, Gene Therapy & Genetic Disease, Neuroscience

## Abstract

Adamantinomatous craniopharyngiomas (aCPs) are complex intracranial neoplasms that arise in the sellar and suprasellar region of the brain, affecting the endocrine and nervous systems, causing severe sequelae. Although most of aCPs are driven by somatic mutations in β-catenin (*CTNNB1*), some cases remain genetically unresolved. Here, we show that disruption of the Wnt-antagonist and tumour suppressor adenomatous polyposis coli (*Apc*) leads to pituitary tumours that present all classic molecular and histopathological hallmarks of aCPs. Mechanistically, we show that a hypomorphic allele of *Apc* leads to tumour development and that bi-allelic loss of *Apc* in the Sox2+ve pituitary stem cells can initiate aCP formation. Apc-driven tumour-initiating cells show a distinct transcriptional signature of p21-mediated senescence-associated secretory phenotype (SASP) with methylomic analyses revealing shared epigenetic alteration of SASP-like inflammatory programme across aCP subtypes. Our data demonstrate a causal role of disruption of *Apc* in driving aCPs, reinforcing the importance of genetic testing for mutations in *APC* in patients with aCP. Furthermore, our work highlights the need to screen patients with familial adenomatous polyposis (FAP) or the spectrum of *APC*-pathogenic syndromes for aCP development early in life.

## Synopsis

The genetic drivers for a subset of adamantinomatous craniopharyngioma (aCPs) remain unknown. We show that disruption of the Wnt antagonist and tumour suppressor adenomatous polyposis coli (Apc) leads to pituitary tumours that recapitulate the classic molecular and histopathological features of aCP.

- Disruption of Apc in pituitary stem cells drives aCP independently of *Ctnnb1* mutations.
- Sox2⁺ cells can initiate APC-driven tumours by overactivation of Wnt/β-catenin pathway through p21-mediated senescence.
- Our findings identify a causative role of Apc in aCP-formation and support *APC* genetic testing and increase surveillance in patients with FAP and *APC*-related syndromes.

## Abstract (web summary)

The genetic drivers for a subset of adamantinomatous craniopharyngioma (aCPs) remain unknown. We show that disruption of the Wnt antagonist and tumour suppressor adenomatous polyposis coli (Apc) leads to pituitary tumours that recapitulate the classic molecular and histopathological features of aCP.

## Graphical abstract

**Figure:**
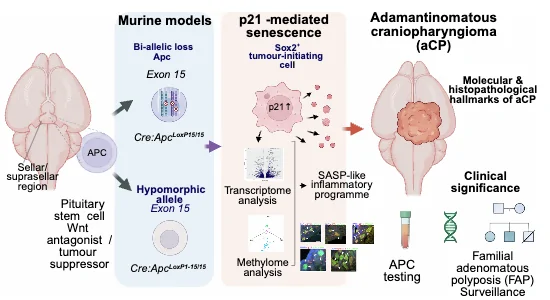

The paper explained **Problem**

Adamantinomatous craniopharyngioma (aCP) is a rare brain tumour that can cause severe and lifelong endocrine, neurological and metabolic complications. Most aCPs are driven by activating mutations in β-catenin (*CTNNB1*), but the genetic drivers of a subset of these tumours remain unknown. Although *APC* mutations have been identified in some aCPs, whether disruption of is sufficient to initiate tumour formation has remained unclear.

**Results**

Using genetically engineered mouse models, we show that disruption of *Apc* in pituitary progenitor/stem cells is sufficient to initiate aCP formation. A hypomorphic *Apc* allele generated postnatal tumours that closely reproduced the histological and molecular features of human aCP. We further demonstrate that Sox2⁺ pituitary stem cells can act as aCP tumour-initiating cells. Transcriptomic and methylome analyses revealed that *Apc* loss activates Wnt/β-catenin signalling and generates tumour-initiating cell clusters characterised by p21-mediated senescence and a pro-inflammatory senescence-associated secretory phenotype (SASP).

**Impact**

Our findings establish APC disruption as a causal mechanism of aCP formation independent of *CTNNB1* mutations, identifying APC-driven tumours as a distinct genetic subtype of aCP. These findings support APC genetic testing in patients with aCP, particularly those lacking *CTNNB1* mutations, and highlight the potential importance of early surveillance for aCP in individuals with familial adenomatous polyposis (FAP) and other germline *APC*-associated syndromes, including Gardner and Turcot syndromes.

## Introduction

Craniopharyngiomas (CPs) are complex neoplasms classified by the World Health Organisation (WHO) as grade 1 benign epithelial tumours typically located within the sellar or suprasellar region of the brain (Louis et al, 2021). They are classified into two distinct subtypes: papillary craniopharyngioma (pCPs), which account for around 10% of all CPs, and adamantinomatous craniopharyngioma (aCPs), representing the remaining 90% (Muller et al, 2019). Molecularly, over 90% of pCPs are driven by mutations in BRAF p.V600E (Brastianos et al, 2014; Larkin et al, 2014), whilst most aCPs are driven by mutations in the *CTNNB1* gene encoding for β-catenin. Although rare, with an incidence of 1.7 per 1,000,000 (Muller et al, 2019; Zacharia et al, 2012), aCPs are the most common suprasellar and non-neuroepithelial intracranial tumour in childhood and have been shown to originate from progenitor/stem cells of the pituitary gland (Andoniadou et al, 2013; Gaston-Massuet et al, 2011). Although classified as benign, aCPs often affect critical neighbouring structures such as the hypothalamus, optic and cranial nerves. This makes total surgical resection technically difficult, resulting in a high rate of recurrence (Karavitaki et al, 2005; Muller et al, 2019).

The prevalence of mutations in β-catenin in human aCPs varies depending on the study (Apps et al, 2020; Brastianos et al, 2014; Hara et al, 2018; He et al, 2021; Hofmann et al, 2006; Malgulwar et al, 2017; Marrero-Gutierrez et al, 2024; Marucci et al, 2015; Wang et al, 2024c). The variability in mutation frequency may be due to small patient cohorts and/or the use of sequencing methods lacking the high depth of reading required for the detection of low allelic frequency variants in these highly heterogeneous tumours. Despite most aCPs being driven by *CTNNB1* mutations, the genetic drivers for a few aCP cases remain unknown (He et al, 2021; Malgulwar et al, 2017; Marrero-Gutierrez et al, 2024; Marucci et al, 2015; Wang et al, 2024c). Previous transgenic murine models have been instrumental to understanding aCP pathogenesis (Andoniadou et al, 2012; Andoniadou et al, 2013; Gaston-Massuet et al, 2011; Gonzalez-Meljem et al, 2017). Pituitary tissue-specific expression of a degradation-resistant form of β-catenin in murine pituitary stem cells has demonstrated that mutations in β-catenin causally drive development of aCPs (Gaston-Massuet et al, 2011). Mutations in negative regulators of the Wnt pathway, that function to degrade β-catenin, such as adenomatous polyposis coli (*APC*), result in increased Wnt/β-catenin signalling, and are involved in the pathogenesis of numerous cancers (Klaus and Birchmeier, 2008). Germline mutations in *APC* cause familial adenomatous polyposis (FAP) (Fodde et al, 2001) and the related spectrum of *APC*-related pathogenic syndromes such as Gardner (Gardner and Richards, 1953) and Turcot Syndrome (Malbari and Lindsay, 2020) that are characterised by colonic adenomas alongside tumours within the brain, bone and skin (Foulkes, 1995; Khattab and Monga, 2025). Recently, an association between FAP and aCPs has been reported in two separate studies (Gorelyshev et al, 2020; Passos et al, 2020). These reports, using whole-exome sequencing, identified 3 cases of aCPs harbouring germline mutations in *APC*, implicating this negative regulator of the Wnt/β-catenin pathway in the pathogenesis of aCPs. However, a causative role for *APC* remains unknown. A further possible association between FAP and aCPs has been hypothesised, as some patients with Gardner syndrome have been reported to develop ectopic aCPs (Alvarez Salgado et al, 2017; Aquilina et al, 2006; Bose et al, 2024; Gabel et al, 2017; Kim et al, 2014; Link et al, 2002; Uemura et al, 2022). However, a causal role of *APC* mutations in the formation of these aCPs, could not be determined due to the lack of tumour genomic sequencing data for these patients (Bose et al, 2024; Powers et al, 2007; Shah et al, 2007; Yan et al, 2009). Moreover, a recent study that analysed two transgenic models to genetically delete *Apc* concluded that genetic deletion of *Apc* in rodent pituitary progenitors was not tumorigenic, resulting in the absence of aCP formation even in older mice (Gonzalez-Meljem et al, 2017). Therefore, although an association between aCPs and mutations in *APC* exists, the causal role for the dysfunction of *Apc* in aCP pathogenesis has remained elusive so far.

Here, we investigate the consequences of *Apc* disruption in pituitary progenitor/stem cells using transgenic mouse models combined with transcriptomic and methylomic analyses. We demonstrate that bi-allelic loss of exon 15 of *Apc* leads to severe embryonic aCP formation, whereas a hypomorphic *Apc* allele produces postnatal tumours that faithfully recapitulate the histological, molecular, and clinical features of human aCPs. We show that *Apc* loss generates specific Wnt-over-activating cell clusters that undergo a p21-mediated senescence-associated secretory phenotype (SASP), producing inflammatory and developmental factors with tumour-promoting potential. Our findings establish *Apc* disruption as an alternative genetic mechanism, leading to Wnt/β-catenin activation, resulting in aCP formation and provide murine models representing a novel genetic subtype of aCP.

## Results

### Bi-allelic loss of *Apc exon 15* in the pituitary embryonic precursors leads to severe aCPs during embryogenesis

**Figure 1:**
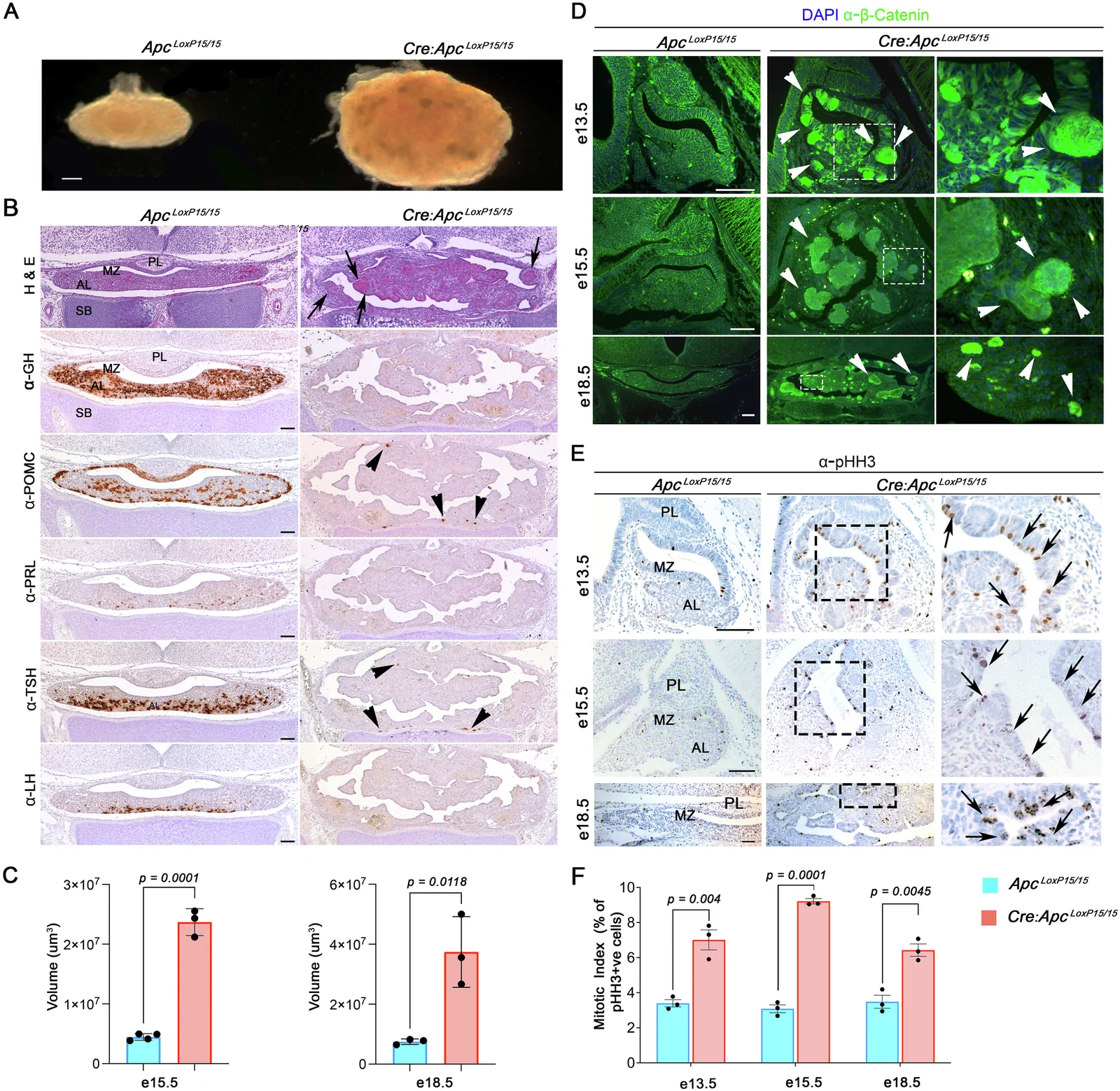
Genetic deletion of *Apc* exon15 in the pituitary progenitors results in large embryonic non-secreting pituitary tumours with classic histological hallmarks of aCPs. (**A**) Wholemount picture of dissected pituitaries at embryonic day e18.5 of a control (*Apc*^*LoxP15/15*^) compared to a mutant *Cre;Apc*^*LoxP15/15*^ exhibiting a large tumour. (**B**) Coronal sections through the pituitary gland at e18.5 stained with H&E reveal a large hyperplastic tumour with epithelial whorl-like structures (arrows) within the tumour parenchyma in the *Cre;Apc*^*LoxP15/15*^ (right column) compared to control pituitary glands (left column). IHC against GH, POMC (melanotrophs and ACTH), PRL, TSH and LH reveals that the *Cre;Apc*^*LoxP15/15*^ tumours are largely devoid of hormone-producing cells, apart from some scattered positive foci of POMC and TSH (arrowheads). (**C**) Volumetric quantifications indicate that the *Cre;Apc*^*LoxP15/15*^ mutant pituitaries have statistically significantly larger volumes compared to control littermates at both e15.5 and e18.5 of gestation. (**D**) Immunofluorescence against β-catenin reveals clusters of nucleo-cytoplasmic β-catenin accumulating cells, which are hallmarks of aCPs in the developing pituitary gland at e13.5, e15.5 and e18.5 in the mutant *Cre;Apc*^*LoxP15/15*^ (white arrowheads) compared to the membranous only β-catenin staining in the control pituitaries. (**E**) IHC against the proliferation marker, phospho-histone H3 (α-pHH3), reveals an increase in pHH3-positive cells in the *Cre;Apc*^*LoxP15/15*^ mutant pituitaries at e13.5, e15.5 and e18.5 compared to control. Quantification of the percentage of pHH3+ve cells revealed a statistically significant increased mitotic index in *Cre;Apc*^*LoxP15/15*^ compared to their control littermates. Right-hand column images in (**D**, **E**) are enlarged images of the squared boxes of the images in the central column. *P* values in (**C**, **F**) were obtained using an unpaired two-tailed Student’s *T* test and data represented as mean ± SEM, from 3/4 pituitaries per genotype. Images are representative of 4–5 embryos per genotype. AL anterior lobe, LH luteinizing hormone, GH growth hormone, H&E haematoxylin and eosin, IHC immunohistochemistry, MZ marginal zone, PL posterior lobe, POMC Pro-opiomelanocortin, PRL prolactin, SB sphenoid bone, TSH thyroid-stimulating hormone. Scale bar in (**A**) represents 100 μm, in (**B**) represents 50 μm; and in (**D**, **E**) represents 200 μm. Source data are available online for this figure.

**Figure 2:**
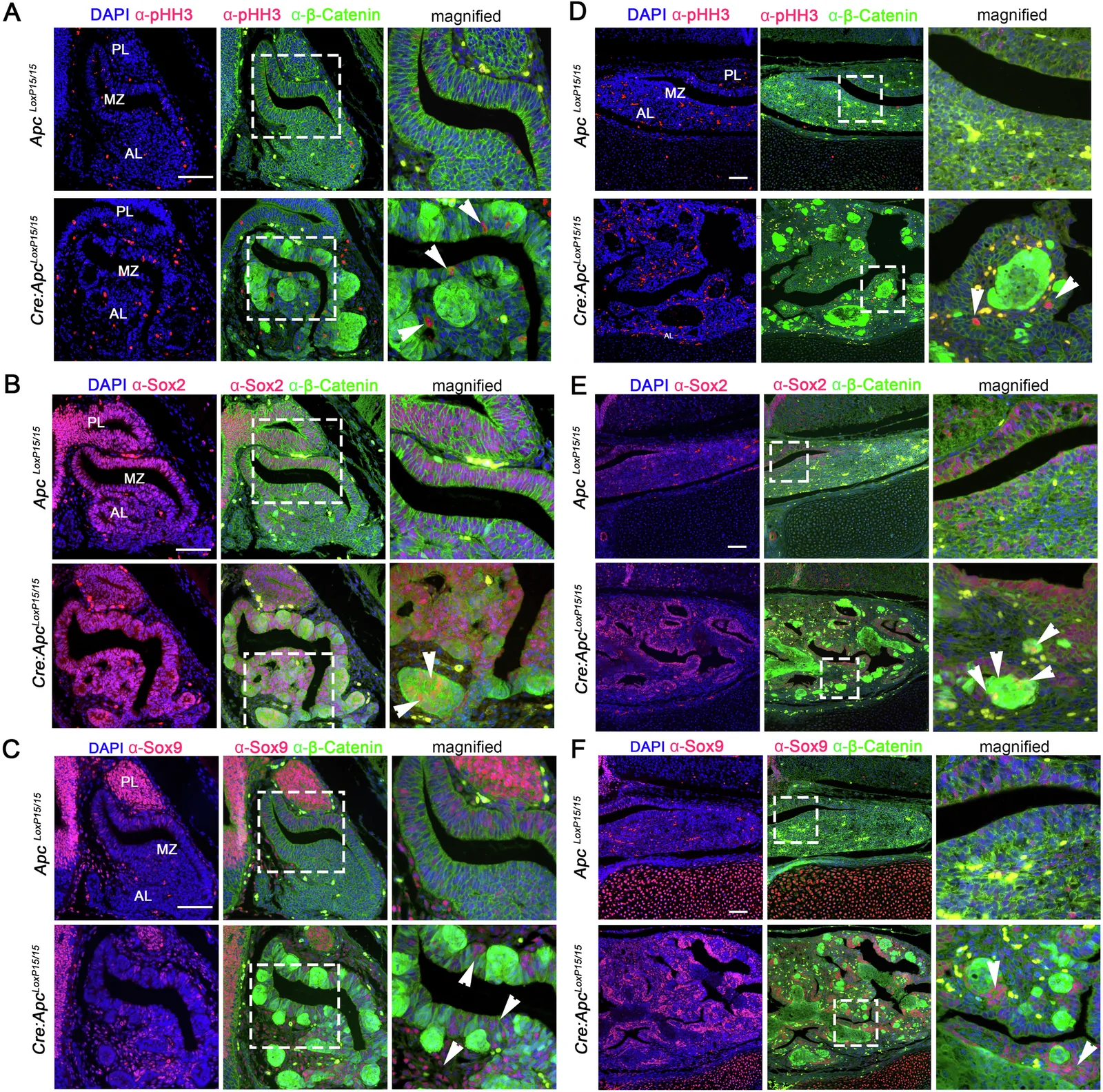
Genetic deletion of *Apc* exon15 (*Cre;Apc*^*LoxP15/15*^) leads to aCP histological hallmarks such as cell clusters of accumulating nucleo-cytoplasmic β-catenin. These express the stem cell marker Sox2, are negative for Sox9 and have low proliferative capacity. (**A**–**F**) Histological sections through the pituitary gland at e13.5 (**A**–**C**) and e18.5 (**D**–**F**) immune-stained with α-pHH3 (**A**, **D**), α-Sox2 (**B**, **E**) and α-Sox9 (**C**, **F**). (**A**, **D**) Double immunofluorescence against pHH3 (red) and β-catenin (green) reveals the presence of clusters of accumulating nucleo-cytoplasmic β-catenin (green), which do not express the proliferation marker pHH3 in the *Cre;Apc*^*LoxP15/15*^. Proliferating cells (pHH3+ve cells, white arrowheads) were always found outside of the β-catenin cell clusters at e13.5 (**A**) and e18.5 (**D**). (**B**, **E**) Double immunofluorescence against β-catenin (green) and α-Sox2 (red) reveals that clusters of accumulating β-catenin express the pituitary stem cell marker Sox2 (white arrowheads). (**C**, **F**) Double immunofluorescence against β-catenin (green) and α-Sox9 (red) shows that clusters of accumulating β-catenin do not express transcription factor Sox9, but rather, the Sox9+ve cells are found surrounding the β-catenin + ve clusters. Note that no clusters of β-catenin were observed in any of the control pituitaries (*Apc*^*LoxP15/15*^) with β-catenin being localised to the cell membrane. Magnified images in the right columns in (**A**–**F**) represent the white-dashed squared areas in the middle column panels. Images are representative of 4 embryos per genotype per gestational stage. AL anterior lobe, MZ marginal zone, PL posterior lobe. Scale bars in (**A**–**C**) represent 200 μm and in (**D**–**F**) represent 100 μm. Source data are available online for this figure.

To determine the causal role of *APC* in craniopharyngioma formation, we employed a murine transgenic approach to delete *exon 15* of *Apc* specifically in embryonic pituitary progenitor cells. We chose to delete the large exon 15 of *Apc* as it contains the mutation hotspot with the most severe reported phenotypes (Rivera et al, 2011). *Exon 15* encompasses 2200 amino acids, including the functional domain responsible for β-catenin degradation and the C-terminal domain involved in microtubule binding (Robanus-Maandag et al, 2010). We used the conditional *Apc* allele in which exon 15 has been flanked with *LoxP* sites *Apc*^*LoxP(Exon15)/LoxP(Exon15)*^ (Robanus-Maandag et al, 2010) to generate a conditional truncated form of *Apc*. To conditionally delete *Apc* within embryonic pituitary progenitor cells, we used the *Prop1:Cre* driver (Davis et al, 2016; Gualtieri et al, 2021), which expresses Cre within pituitary progenitors from embryonic day (e) 10 of gestation, to generate homozygotes *Prop1:Cre;Apc*^*LoxP(Exon15)/LoxP(Exon15)*^ (hereafter referred to as *Cre:Apc*^*LoxP15/15*^ for simplicity). Homozygotes *Cre:Apc*^*LoxP15/15*^ pups died perinatally, with none of the mutant pups reaching postnatal day 7. Dissections of pituitary glands revealed large tumours in all *Cre:Apc*^*LoxP15/15*^ embryos compared to their control (*Apc*^*LoxP15/15*^) littermates (Fig. 1A). Histological analyses using haematoxylin and eosin staining (H&E) and antibodies against terminally differentiated hormone producing cells, revealed that *Cre:Apc*^*LoxP15/15*^ mutant embryos exhibit large pituitary tumours that are almost devoid of all terminally differentiated hormone producing cells (Fig. 1B). These tumours are characterised by aCP-histopathological features such as whorl-like structures consisting of cell clusters of nucleo-cytoplasmic positive β-catenin (Fig. 1D). Abnormally larger pituitary glands were observed as early as embryonic day (e) 11 and progressively worsen throughout gestation, exhibiting statistically significant increase in pituitary volume (Fig. 1C) secondary to increased mitotic index (Fig. 1E,F). To further characterise these tumours, we performed double immunofluorescence with β-catenin and the proliferation marker phospho-histone H3 (α-pHH3) or the pituitary stem cell markers Sox2 or Sox9, as aCP-histological hallmarks (Andoniadou et al, 2012; Buslei et al, 2005; Garcia-Lavandeira et al, 2012; Gaston-Massuet et al, 2011). Double immunofluorescence at e13.5 and e18.5 revealed large clusters of nucleo-cytoplasmic accumulating β-catenin, which were negative for pHH3. Notably, most of the dividing cells were located surrounding the β-catenin+ve cell clusters (Fig. 2A,B). As previously described in β-catenin-driven aCP tumours (Andoniadou et al, 2013; Gaston-Massuet et al, 2011), these β-catenin+ve clusters express the pan-pituitary stem cell marker Sox2 (Fig. 2C,D) with Sox9+ve cells localised around the β-catenin+ve cell clusters (Fig. 2E,F). To confirm the efficiency of recombination of the exon 15 of *Apc*, we used RT-qPCR and identified 100% deletion of *exon 15* by our *Prop1:Cre* driver. To confirm the RT-qPCR results, we used a western blot to detect wild-type (Wt)-Apc protein using an antibody that detects only the C-terminal part of Apc (absent upon deletion of Apc exon 15), and showed absence of Wt-Apc protein in the *Cre:Apc*^*LoxP15/15*^-derived pituitary progenitor cells (Appendix Fig. S1d,e).

**Figure EV1:**
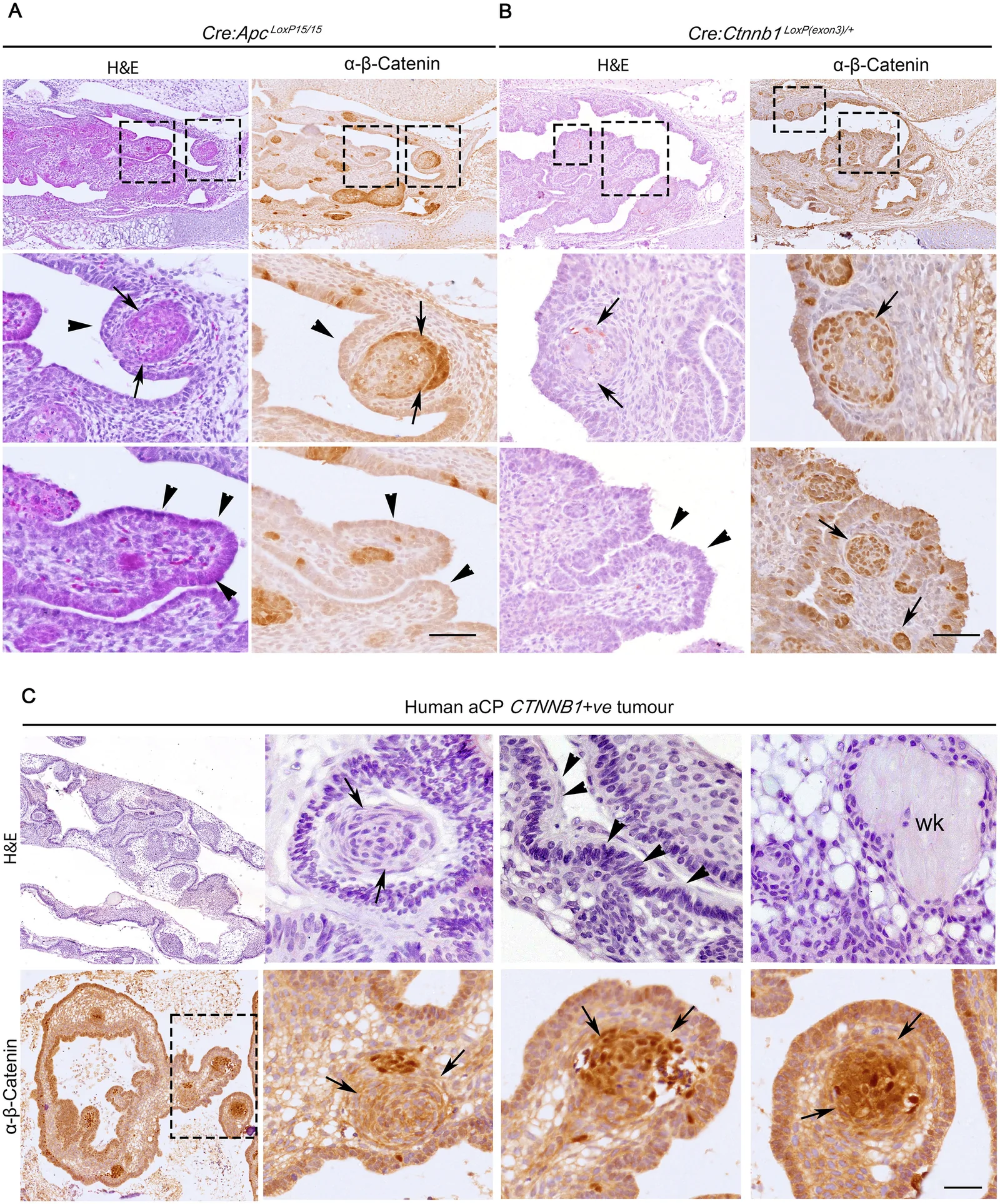
Histopathological features across aCP tumour subtypes. High-magnification images of tumours from embryonic *Cre:Apc*^*LoxP15/15*^ (**A**), murine embryonic *Ctnnb1-*driven tumours (*Cre:Ctnnb1*^*LoxP(exon3)/+*^) (**B**) and human *Ctnnb1+ve* aCP (**C**) reveal common histological diagnostic hallmarks of aCPs. (**A**) High magnification of H&E (from Fig. 1B) or IHC (against β-catenin) of sections from embryonic *Cre:Apc*^*Loxp15/15*^ tumours revealed the presence of classical histopathological features of aCPs, such as whorl-like cell clusters (arrows) and palisading epithelium (arrowheads) that form finger-like protrusions. Consecutive sections stained against β-catenin revealed that these whorl-like cell clusters express nuclear β-catenin (arrows), which is a diagnostic marker for aCPs. Likewise, these histological features were present in sections from murine *Ctnnb1*+ve driven tumours (**B**) (*Cre:Ctnnb1*^*LoxP(exon3)/+*^) with whorl-like clusters of cells (arrows) which express nuclear β-catenin (arrows) and form finger-like protrusions. (**C**) Representative section of a *CTNNB1+ve* human aCP stained with H&E or IHC against β-catenin exhibiting the classical histological diagnostic features of aCP, such as: whorl-like clusters of cells (arrows), palisading epithelium (arrowheads), deposits of wet keratin (wk) (which are also present in our mid-stage Apc-tumours from our murine *Cre:Apc*^*Loxp1-15/15*^ Fig. 4E). These tumours present clusters of accumulating β-catenin (arrows). Images are representative of 4 tumours per genotype. H&E haematoxylin and eosin, IHC immunohistochemistry, wk wet keratin. Scale bars represent 50 μm.

We then compared the histological features of the *Cre:Apc*^*LoxP15/15*^ embryonic tumours to the previously reported murine tumours driven by deletion of *Ctnnb1* (*exon3*) (*Cre:Ctnnb1*^*LoxP(exon3)/+*^) and to the *CTNNB1+ve* human aCPs and identified similar histological hallmarks of aCPs across these tumour subtypes (Fig. EV1). Together, our results demonstrate that bi-allelic loss of *Apc* exon 15 within the pituitary progenitor cells results in intrauterine large aCPs associated with early postnatal lethality due to tumour size and severity, with the absence of differentiation of hormone-secreting cells in the pituitary gland.

### A hypomorphic allele for *Apc* results in postnatal aCP tumour formation

**Figure 3:**
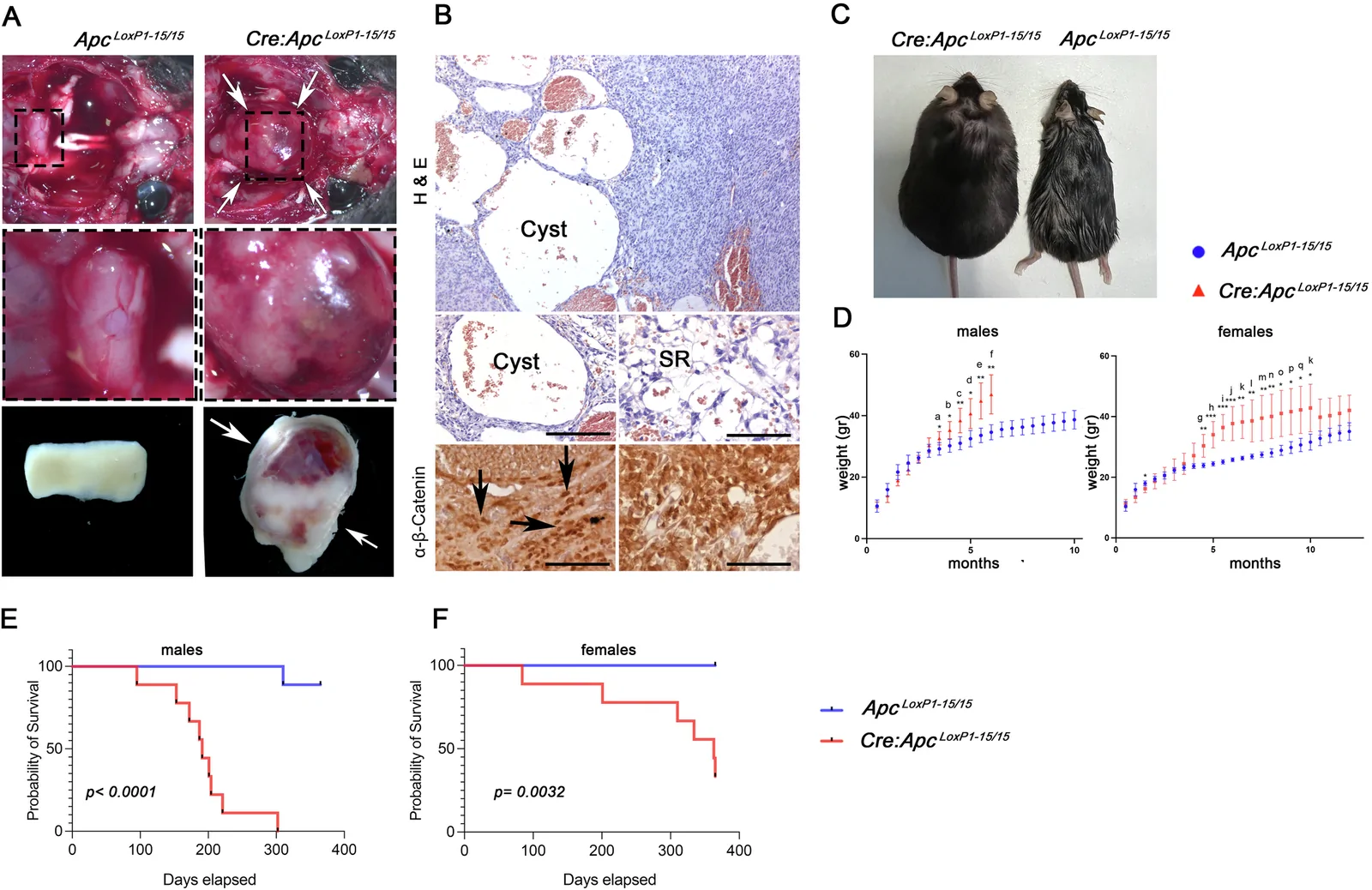
A hypomorphic allele of *Apc* (*Cre:Apc*^*LoxP1-15/15*^) develops large pituitary tumours postnatally. (**A**) Picture after removal of the scalp and brain of a 6-month-old *Cre:Apc*^*LoxP1-15/15*^ mutant revealed pituitary tumours (white arrows). (**B**) H&E sections of the tumours reveal large cystic components (Cyst) and some cells reminiscent of stellate reticulum (SR) cells. IHC against β-catenin shows cells with nuclear positivity within some cell clusters (black arrows). (**C**, **D**) Male and female hypomorphic animals exhibit statistically significant weight gain starting from 4 months of age, with all mutant animals exhibiting obesity compared to their control littermates (*Apc*^*LoxP1-15/15*^*)* (**D**). Kaplan–Meier survival curves from males (**E**) and females (**F**) show that hypomorphic *Cre:Apc*^*LoxP1-15/15*^ mutant animals have shorter lifespans compared to their Wt littermates. Note that males (**E**) have a shorter life span compared to females (**F**), with a 50% probability of survival at 6 months (180 days), whilst females exhibited a longer life span (320 days). Kaplan–Meier curves represent 9 mutants and 9 Wt of males and females of each genotype. *P* values in (**D**) (a *P* = 0.043; b *P* = 0.017; c *P* = 0.008; d *P* = 0.014; e *P* = 0.0075; f *P* = 0.00094; g *P* = 0.0067; h *P* = 0.0016; i *P* = 0.00056; j *P* = 0.0019; k *P* = 0.0017; i *P* = 0.0074; m *P* = 0.0090; n *P* = 0.009, o *P* = 0.011; p *P* = 0.013; q *P* = 0.017; k *P* = 0.021) represent and were obtained using multiple unpaired Student’s *T* test and data represented as mean ± SEM. (**E**, **F**) *P* values of survival curves were obtained by a Log-rank Mantel-Cox test. H&E haematoxylin and eosin, SR stellate reticulum cells. Scale bars in (**B**) represent 100 μm. Source data are available online for this figure.

To overcome the early lethality observed in the *Cre:Apc*^*LoxP15/15*^ animals, we took a genetic approach to generate another mutant allele of *Apc*. To achieve this, we utilised the *Apc*^*LoxP(Exon1-15)*^ line in which *LoxP*-sites have been introduced to flank all the *Apc* exons, from exon 1 to exon 15, enabling deletion of the entire *Apc* locus (Cheung et al, 2010). Homozygotes *Prop1:Cre*:*Apc*^*LoxPExon 1-15/LoxPExon1-15*^ (hereby referred to as *Cre:Apc*^*LoxP1-15/1-15*^ for simplicity) exhibited enlarged pituitary glands due to increased mitotic index (Appendix Fig. S1a,b) with decreased number of growth hormone-producing cells (Appendix Figure S1a). However, no tumour formation occurred in any of the mutant mice (*Cre:Apc*^*LoxP1-15/1-15*^) analysed after 2 years, other than hyperplasia of the anterior pituitary gland. This prompted us to investigate the efficiency of Cre recombination of exons *1-15* at the *Apc* locus by RT-qPCR in our homozygote *Cre:Apc*^*LoxP1-15/1-15*^ pituitaries. Importantly, we identified that 16.37% of Wt *Apc* exon 15 mRNA was being produced, indicating inefficient recombination of the *Apc* locus (Appendix Fig. S1d). This result suggests that a small amount of Wt *Apc* mRNA is sufficient to prevent tumour formation, resulting in pituitary hyperplasia instead of tumours. Hence, we reasoned that further reduction of Wt *Apc* levels by combining the *Cre:Apc*^*LoxP15/+*^ (with 100% recombination efficiency) with the *Cre:Apc*^*LoxP 1-15/+*^ alleles to generate a compound heterozygote *Cre*;*Apc*^*LoxP1-15/15*^ could result in a milder phenotype compared to the *Cre:Apc*^*LoxP15/15*^. Indeed, all *Cre:Apc*^*LoxP1-15/15*^ compound mutants survived to adult compared to the early perinatal lethality observed in the *Cre:Apc*^*LoxP15/15*^ pups (Fig. 1). All *Cre:Apc*^*LoxP1-15/15*^ compound mutants developed large pituitary tumours postnatally, leading to tumour-associated co-morbidities such as severe obesity and reduced life expectancy (Fig. 3A–F). Obesity secondary to tumour formation was apparent in all mutant *Cre:Apc*^*LoxP1-15/15*^ mice from 4 months of age (Fig. 3C,D), an important phenotype that has not been observed in previously published murine aCP models (Gaston-Massuet et al, 2011). Histological analyses of these tumours revealed the presence of large cystic components, stellar reticular-like cells and nucleo-cytoplasmic β-catenin+ve cell clusters, all of which are histological diagnostic hallmarks of aCPs (Figs. 3B and EV1). Interestingly, Kaplan–Meier survival curves revealed a gender difference in survival, with female mice surviving longer than males (Fig. 3E,F).

**Figure 4:**
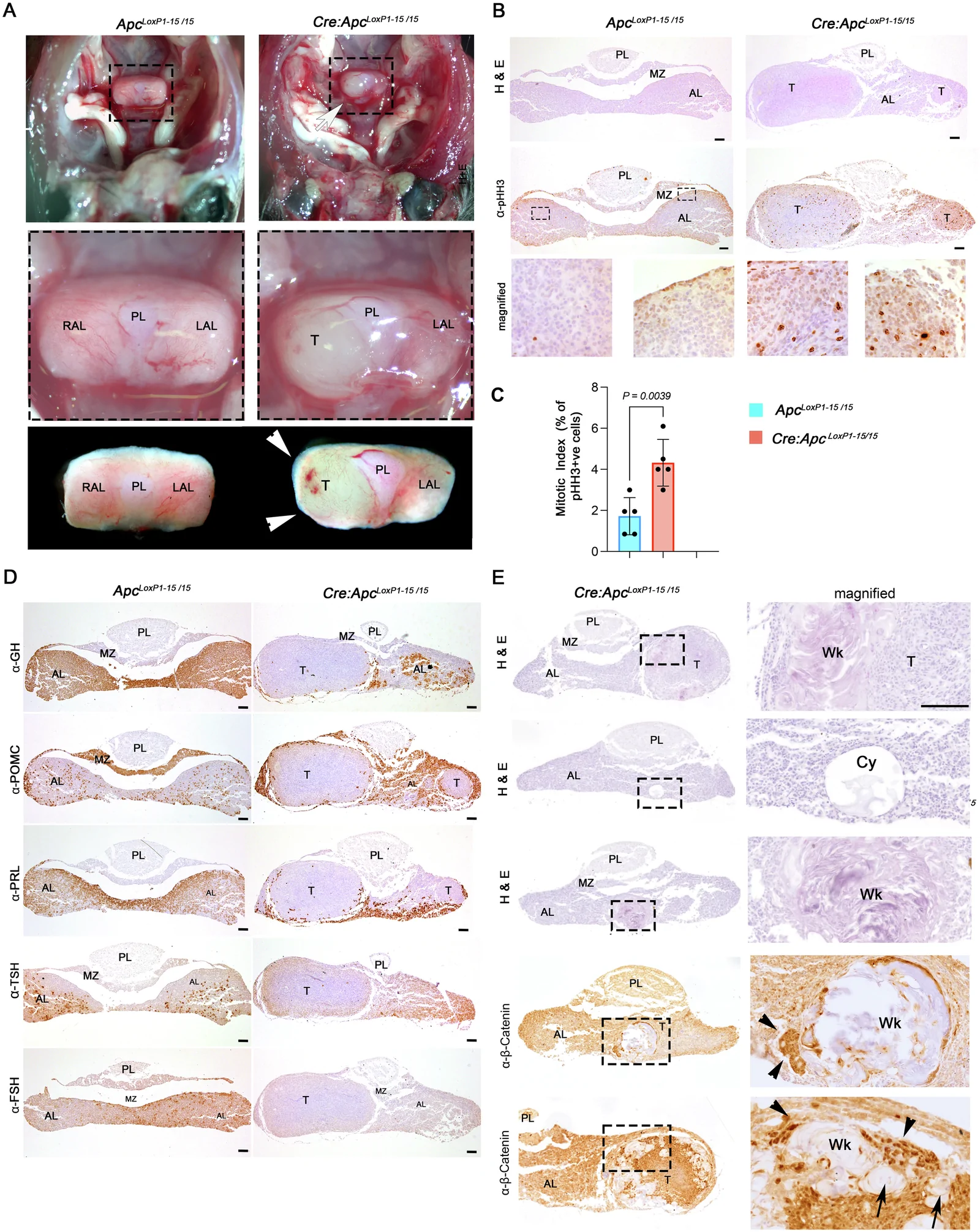
Mid-stage tumours from *Cre:Apc*^*LoxP1-15/15*^ hypomorphs exhibit classic histological hallmarks of aCPs. (**A**) Picture after removal of the scalp and brain in a 3-month-old, *Cre:Apc*^*LoxP1-15/15*^ mutants revealed hyperplastic pituitaries with small tumours (white arrows & T). (**B**) H&E staining of coronal sections through the *Cre:Apc*^*LoxP1-15/15*^ mutant pituitaries revealed developing tumours within the pituitary parenchyma of differing sizes (marked as T). (**C**) These tumours have increased pHH3+ve cells, resulting in a higher mitotic index. (**D**) IHC against GH, POMC (melanotrophs and ACTH), PRL, TSH and FSH reveals that the developing tumours are devoid of hormone-producing cells. (**E**) H&E staining of coronal sections through *Cre:Apc*^*LoxP1-15/15*^ mutant pituitaries revealed histological hallmarks of aCPs, such as deposits of wet keratin (Wk) and small cysts (Cy). IHC against β-catenin in mutant pituitaries revealed the presence of cell clusters of nucleo-cytoplasmic β-catenin (black arrowheads) surrounding nodules of wet keratin (Wk, black arrows). *P* value in (**C**) was obtained using unpaired two-tailed Student’s *T* test, and data represented as mean ± SEM. Images and MI in (**C**) are representative of 5 pituitaries per genotype. AL anterior lobe, FSH follicle-stimulating hormone, GH growth hormone, PL posterior lobe, PRL prolactin, POMC pro-opiomelanocortin, MZ marginal zone, RAL right anterior lobe, LAL left anterior lobe, T tumours, TSH thyroid-stimulating hormone, Wk wet keratin. Scale bars in (**B**, **D**) represent 100 μm, and in (**E**) represent 50 μm. Source data are available online for this figure.

**Figure 5:**
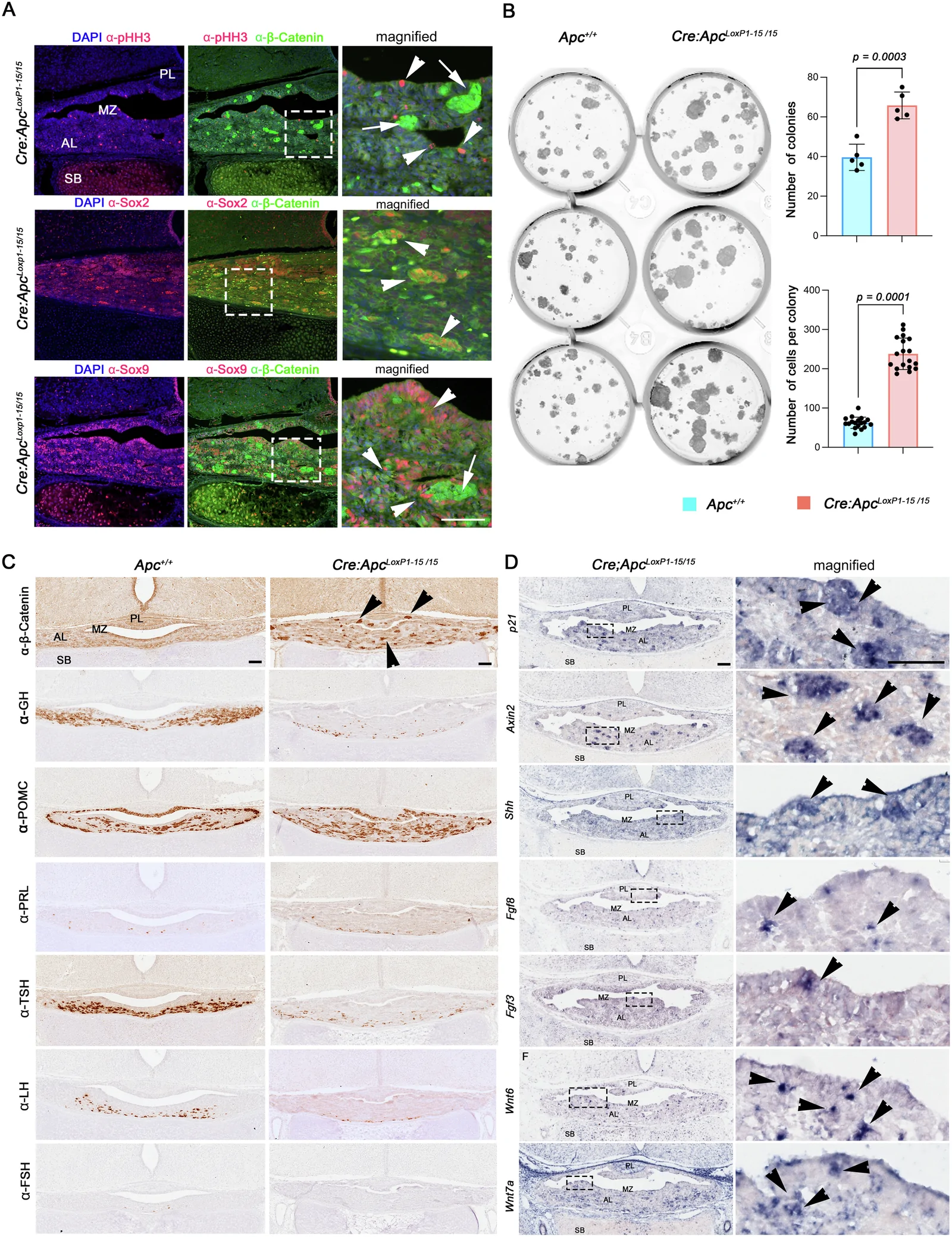
Embryonic pituitaries from *Cre:Apc*^*LoxP 1-15/15*^ embryos exhibit all the features of early aCP development. (**A**) Double immunofluorescence of coronal sections through the developing pituitary gland from the *Cre:Apc*^*LoxP 1-15/15*^ embryos at e18.5 against pHH3, Sox2 and Sox9 with β-catenin. Clusters of nucleo-cytoplasmic β-catenin + ve cells are present throughout the pituitary parenchyma (green, white arrows) with pHH3+ve cells (red, white arrowheads) scattered around the clusters of β-catenin (white arrows in the magnified image). All clusters of accumulating β-catenin express the pituitary stem cell marker Sox2 (red, white arrowheads), and the Sox9+ve cells are located around the β-catenin + ve clusters. The right column images are magnified images from the white squared areas in the middle column. (**B**) Pituitary stem cell culture from hypomorphic *Cre:Apc*^*LoxP 1-15/15*^ mutants exhibits higher clonogenic potential evidenced by both increased number of colonies and number of cells per colony compared to Wt pituitary stem cells. (**C**) IHC against β-catenin in coronal sections through the pituitary and brain at e18.5 revealed clusters of cells with accumulating β-catenin in *Cre:Apc*^*LoxP 1-15/15*^ (right column, black arrowheads) compared to Wt (left column). IHC against GH, TSH, LH and FSH reveals that *Cre:Apc*^*LoxP 1-15/15*^ hypomorphic mutant pituitaries exhibit a reduction in these hormone-producing cells compared to their Wt littermates, whilst POMC (melanotrophs and ACTH) and PRL show similar expression between genotypes. (**D**) In situ hybridisation on coronal sections through the pituitary gland of *Cre:Apc*^*LoxP1-15/15*^ mutants reveals upregulation of the canonical downstream Wnt-effector *Axin2* and the senescence marker *p21* in a cell cluster fashion. The developmental factors *Shh*, *Fgf8*, *Fgf3* and *Wnt6*, *Wnt7a* were highly upregulated within cell clusters throughout the pituitary parenchyma. Right column panels are magnified images from the dotted square areas in the left column. *P* values in (**B**) were obtained using an unpaired two-tailed Student’s *T* test, and data represented as mean ± SEM. Images are representative of five embryos per genotype. AL anterior lobe, LH luteinising hormone, GH growth hormone, IHC immunohistochemistry, MZ marginal zone, PL posterior lobe, PRL prolactin, POMC Pro-opiomelanocortin, SB sphenoid bone, TSH thyroid-stimulating hormone. Scale bars in (**B**) represent 50 μm; and in (**C**, **D**) represent 100 μm. Source data are available online for this figure.

Characterisation of tumours at 3 months of age revealed all the typical histological diagnostic hallmarks of human aCPs, including cysts, nucleo-cytoplasmic β-catenin+ve cell clusters, large foci of wet keratin and increased mitotic index (Fig. 4A–E). Immunostaining of the pituitary gland for terminally differentiated hormones revealed the absence of hormone-producing cells within the developing tumour in keeping with the non-hormone-secreting nature of aCPs (Fig. 4D). H&E staining revealed wet keratin deposits, cysts, and nucleo-cytoplasmic β-catenin+ve cell clusters often surrounding the wet keratin foci (Fig. 4E), features that are also observed in human β-catenin+ve aCPs (Fig. EV1C). To analyse the early tumour characteristics of the *Cre:Apc*^*LoxP1-15/*15^ hypomorphs at e18.5, double immunofluorescence against β-catenin and pHH3, Sox2 or Sox9 was performed. These early tumours exhibit clusters of accumulating nucleo-cytoplasmic β-catenin, which are negative for pHH3, with dividing cells localised to the surrounding of β-catenin+ve cell clusters (Fig. 5A). The β-catenin+ve cell clusters were all positive for the pituitary stem cell marker Sox2, with Sox9+ve cells localised around the β-catenin+ve cell clusters (Fig. 5A). Clonogenic assays found a statistically significant increase in the number of stem cell colonies and the number of cells per colony in mutant *Cre:Apc*^*LoxP1-15/15*^ derived pituitary stem cells at e18.5 compared to Wt pituitaries, indicating that mutant derived pituitary stem cells have more proliferative capacity (Fig. 5B). At this embryonic stage, IHC for terminally differentiated hormones revealed a decrease in hormone-producing cells compared to Wt with a clear decrease in GH, TSH, FSH and LH, whilst no differences were observed in other hormones (Fig. 5C). Next, RT-qPCR was used to identify the level of recombination in the *Cre:Apc*^*LoxP1-15/15*^ compound mutants. This identified that Wt *Apc* exon15 mRNA was present in small amounts (7.5% compared to Wt cells) whilst the exon 15 from the *Cre:Apc*^*LoxP15/15*^ was fully deleted (Appendix Fig. S1d). To verify our RT-qPCR results, we studied the expression of Wt-Apc protein from cultured pituitary progenitor cells derived from either *Cre:Apc*^*LoxP15/1*5^ or *Cre:Apc*^*LoxP1-15/15*^ using an antibody against the C-terminal end of Apc (which does not detect the truncated floxed-out exon15 of *Apc*). Whilst no Wt-Apc protein was detected in the *Cre:Apc*^*LoxP15/15*^, expression of Wt-Apc C-terminal protein was detected in the hypomorph *Cre:Apc*^*loxP1-15/15*^ derived cells (Appendix Fig. S1e). This result shows that *Cre:Apc*^*LoxP1-15/15*^ expresses small amounts of Wt Apc and is therefore a hypomorph allele. Several genetic studies have shown that small amounts of Wt-Apc are sufficient to degrade β-catenin, resulting in partial restoration of colon adenoma formation (Buchert et al, 2010; Clarke, 2006; Dow et al, 2015). Studies using hypomorph allelic series showed that only 10% of Wt-Apc expression was sufficient to reduce tumour burden in vivo and in vitro (Li et al, 2005). Hence, we hypothesise that small amounts of Wt Apc in the *Cre:Apc*^*loxP1-15/15*^ are sufficient to decrease tumour burden, leading to a less severe phenotype, but still enough to result in postnatal tumour formation.

**Figure EV2:**
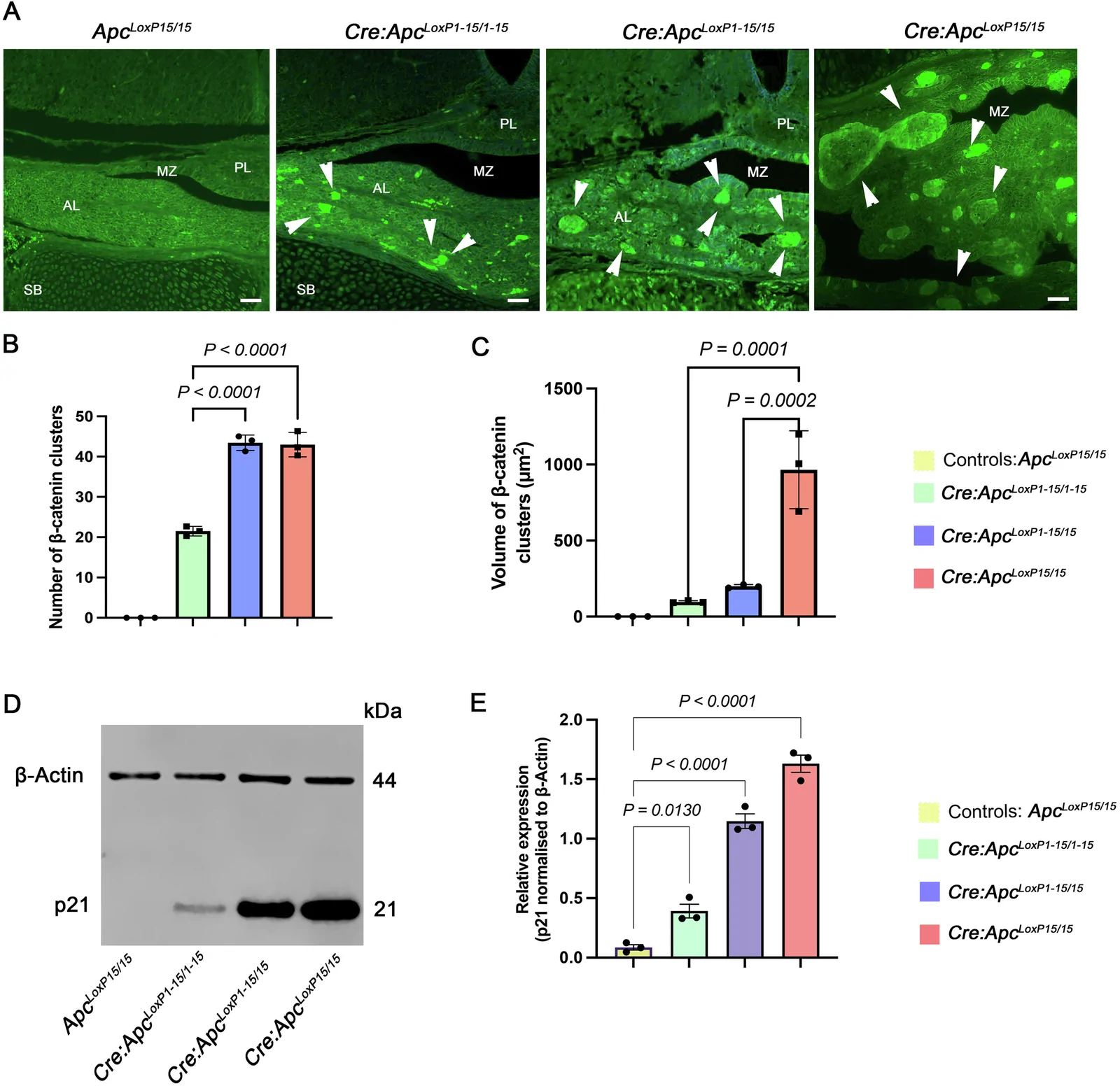
The *Cre;Apc*^*LoxP15/15*^ mutant pituitaries exhibit larger nucleo-cytoplasmic β-catenin accumulating cell clusters and express higher levels of the senescence marker p21 compared to the hypomorph alleles *Cre;Apc*^*LoxP1-15/15*^, *Cre;Apc*^*LoxP1-15/1-15*^*.* (**A**) Immunofluorescence against β-catenin (green) on coronal sections through the pituitary glands reveals that the *Cre;Apc*^*LoxP15/15*^ mice have larger nucleo-cytoplasmic β-catenin cell clusters compared to the hypomorph allele *Cre;Apc*^*LoxP1-15/15*^. The *Cre;Apc*^*LoxP1-15/1-15*^ exhibits small cell clusters (arrowheads) which are not sufficient to drive tumour formation, leading to hyperplasia only (also in Appendix Fig. S1). (**B**) The number of clusters (**B**) and the volume of β-catenin cell clusters (**C**) are significantly higher in the *Cre;Apc*^*LoxP15/15*^ and the *Cre;Apc*^*LoxP1-15/15*^ compared to the *Cre;Apc*^*LoxP1-15/*1-15^ milder phenotype. (**D**) Western blot against p21 from progenitor/stem cells derived from the 3 different genotypes and controls (*Apc*^*LoxP15/15*^), reveals that p21 is highly expressed in the *Cre;Apc*^*LoxP15/15*^ and to a lesser extent in the *Cre;Apc*^*LoxP1-15/15*^ compared to the *Cre;Apc*^*LoxP1-15/*1-15^. (**E**) Quantification of the relative protein expression of p21 normalised to β-actin shows that levels of p21 increase with the severity of the phenotype, with cells derived from *Cre;Apc*^*LoxP15/15*^ expressing the highest levels of p21, suggesting a stronger p21-mediated senescence function. p21 was found to be upregulated in the β-catenin cell clusters by double immunofluorescence (see Appendix Fig. S7). Images in (**A**) are representative of 4 embryos per genotype. *P* values in (**B**, **C**, **E**) were obtained using one-way ANOVA and data represented as mean ± SEM, from three pituitaries per genotype and in triplicate independent experiments. AL anterior lobe, MZ marginal zone, PL posterior lobe, SB sphenoid bone. Scale bars in (**A**) represent 50 μm.

We then compared the phenotypic outcomes of our 3 different genetic models and studied the number and size of β-catenin+ve clusters as readout of overactivation of the Wnt/β-catenin pathway (Fig. EV2). Interestingly, we found that the most severe phenotype (full deletion of *Apc* exon15, *Cre:Apc*^*LoxP15/15*^*)* exhibits very large β-catenin+ve cell clusters compared to the postnatal tumours *Cre:Apc*^*LoxP1-15/15*^, whilst the milder hyperplasia phenotype (without tumour formation, *Cre:Apc*^*LoxP1-15/1-*15^ Appendix Fig. S1) exhibits a considerably reduced number and size of β-catenin+ve clusters compared to the other two models. Together, these results show that the size of the β-catenin+ve cell clusters correlates with the phenotypic severity.

β-catenin-mutated+ve aCPs are characterised by the presence of clusters of accumulating nucleo-cytoplasmic β-catenin that undergo SASP and secrete factors into the tumour microenvironment (Apps et al, 2018; Gonzalez-Meljem et al, 2017; Prince et al, 2024). We, therefore, analysed by in situ hybridisation the expression of some markers of β-catenin+ve clusters previously reported in mouse and human aCPs (Andoniadou et al, 2012; Gaston-Massuet et al, 2011). All clusters analysed from the *Cre:Apc*^*loxP1-15/15*^ hypomorphic pituitaries expressed the canonical Wnt-activating target, *Axin2*, indicating that the β-catenin+ve cell clusters over-activate the Wnt canonical pathway. Moreover, these clusters over-express the developmentally secreted factors *Wnt6*, *Wnt7a*, *Fgf3, Fgf8* and *Shh* in a cluster fashion (Fig. 5D). Importantly, these clusters upregulate the senescence-associated marker *Cdkn1a* (*p21*) in accordance with activation of SASP described in both human (Apps et al, 2018; Prince et al, 2024) and murine aCPs (Andoniadou et al, 2012; Gonzalez-Meljem et al, 2017). Taken together, our results demonstrate that a hypomorphic allele of *Apc* leads to tumour formation postnatally, exhibiting all the characteristic histological and molecular features of aCP tumours, including associated co-morbidities such as tumour-induced obesity. These results further demonstrate a causal role of *Apc* dysfunction in the development of aCPs.

### Bi-allelic loss of *Apc* exon 15 in the Sox2+ve pituitary stem cells is sufficient to initiate aCP tumour formation

**Figure 6:**
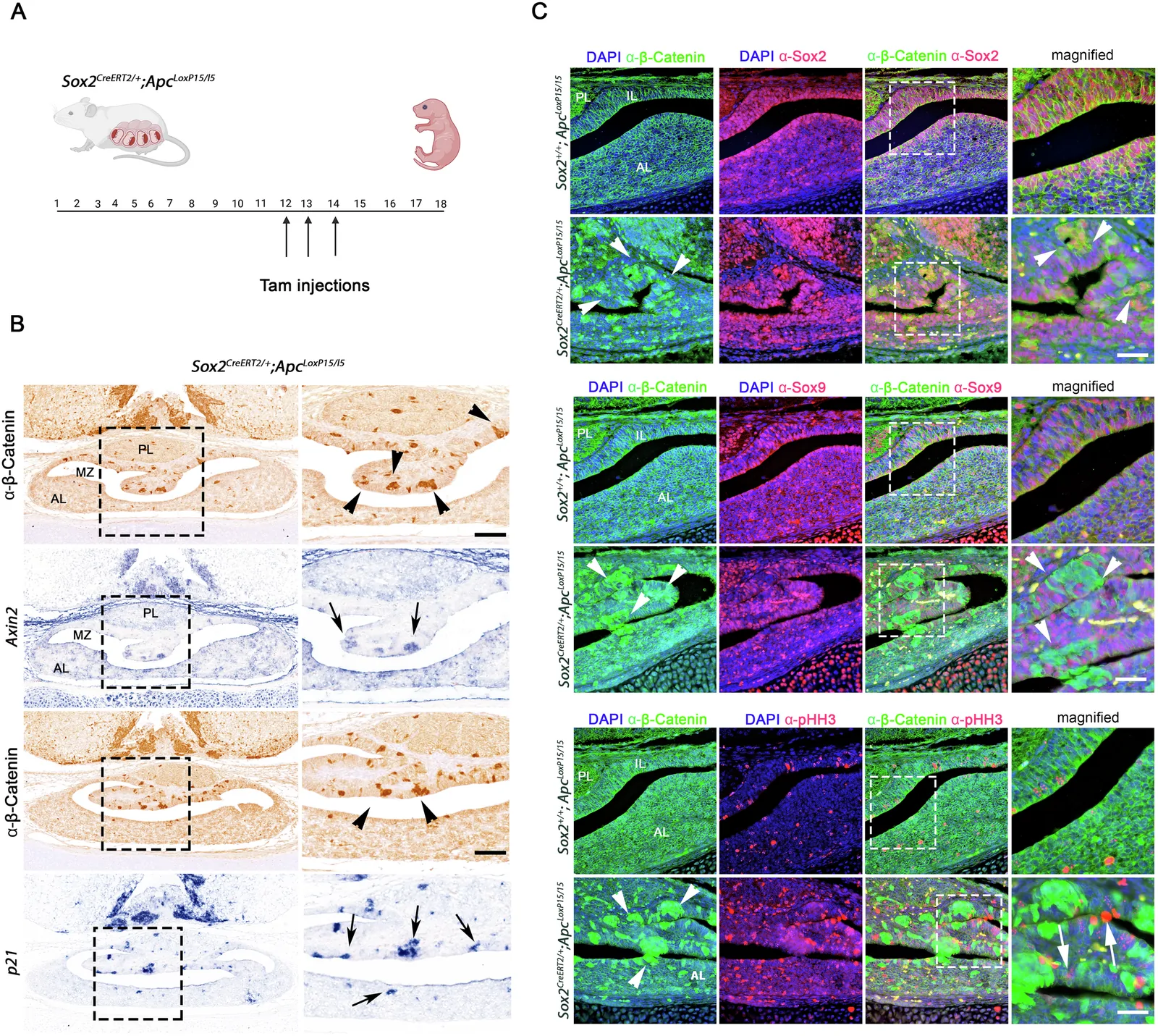
Deletion of *Apc* exon15 in the Sox2+ve pituitary stem cells results in early aCP formation. (**A**) Experimental diagram: to achieve deletion of *Apc* exon15 (*Sox2*^*CreERT2/+*^*;Apc*^*LoxP15/15*^), pregnant females were Tam injected (0.1 mg/g of body weight) with 3 consecutive daily injections at days e12, e13 and e14 of pregnancy. Embryos were dissected at e18.5, and pituitary glands were analysed by in situ hybridisation and immunostaining. (**B**) IHC against β-catenin revealed the presence of multiple cell clusters of nucleo-cytoplasmic accumulating β-catenin (arrowheads). In situ hybridisation on consecutive sections for *Axin2* and the senescence marker *p21* reveals the upregulation of *Axin2* and *p21* within the β-catenin positive clusters (arrows). Pictures in the right column are magnified images from the squared dashed boxes in the left column. (**C**) Double immunofluorescence against Sox2, Sox9, pHH3 (red) and β-catenin (green) revealed that Tam-induced *Sox2*^*CreERT2/+*^*;Apc*^*LoxP15/15*^ embryos develop early aCP lesions characterised by clusters of nucleo-cytoplasmic accumulating β-catenin cells (green, white arrowhead), which co-express the pituitary stem cell marker Sox2. Sox9+ve cells and proliferating cells (pHH3+ve cells) are found around the β-catenin + ve cell clusters (white arrows). Images are representative of three embryos per genotype. AL anterior lobe, IL intermediate lobe, IHC immunohistochemistry, MZ marginal zone, PL posterior lobe, Tam tamoxifen. Scale bars in (**B**, **C**) represent 50 μm. Source data are available online for this figure.

Previous studies using a conditional transgenic line have shown that Sox2+ve pituitary stem cells bearing oncogenic β-catenin (*Ctnnb1*^*LoxpExon3/*+^) can initiate aCP tumour formation (Andoniadou et al, 2013). To further prove a causative role of *Apc* in aCP formation, we employed an inducible *Sox2*^*CreERT2/+*^ transgenic line (Arnold et al, 2011) in which tamoxifen (Tam) induced Cre is expressed within Sox2+ve stem cells. To enable the deletion of *exon 15* of *Apc*, we crossed the *Sox2*^*CreERT2*/+^ to the *Apc*^*LoxP(Exon15)/LoxP(Exon15)*^ strain to generate *Sox2*^*CreERT2/+*^*;Apc*^*LoxP(Exon15)/LoxP(Exon15)*^ embryos (hereafter referred to as *Sox2*^*CreERT2/+*^*;Apc*^*LoxP15/15*^ for simplicity). Deletion of *Apc exon 15* was achieved with 3 consecutive daily injections of Tam at 0.1 mg/g/mouse during pregnancy starting at e12, and embryos were analysed at e18.5 (Fig. 6A). Interestingly, Tam-induced *Sox2*^*CreERT2/+*^*;Apc*^*LoxP15/15*^ embryos exhibited hyperplasia of the anterior pituitary gland with the presence of clusters of accumulating nucleo-cytoplasmic β-catenin (Fig. 6B,C). In situ hybridisation on consecutive sections revealed β-catenin+ve clusters expressing *Axin2* and the senescence marker *p21* (Fig. 6B), similar to our *Apc*-driven models (Figs. 2 and 5). These clusters are Sox2+ve, have low proliferating capacity (negative for pHH3) and are surrounded by Sox9+ve cells (Fig. 6C) in keeping with aCPs (Andoniadou et al, 2013; Apps et al, 2018; Gaston-Massuet et al, 2011).

Since Sox2 is expressed in several stem cell compartments and the *Sox2*^*CreERT2/+*^ line has previously been used to drive *Ctnnb1* mutations in the murine olfactory epithelium (OE) (Engel et al, 2016), we studied the effect of deletion of exon 15 of *Apc* in the Sox2+ve cells of developing OE. We observed that the OE of Tam-induced *Sox2*^*CreERT2/+*^*;Apc*^*LoxP15/15*^ exhibits hyperplasia and disorganised morphology compared to the control littermates (Appendix Fig. S2), supporting the role of Wnt/β-catenin canonical pathway in OE homeostasis (Engel et al, 2016).

We then tested if genetic deletion of *Apc* exon15 in postnatal Sox2+ve pituitary stem cells could lead to aCP tumour initiation in adulthood. To this end, we induced postnatal pups *Sox2*^*CreERT2/+*^*;Apc*^*LoxP15/15*^ with Tam-injections at postnatal days 4 and 5 and histologically analysed the presence of tumours at 10 weeks of age (Appendix Fig. S3). Interestingly, Tam-induced *Sox2*^*CreERT2/+*^*;Apc*^*LoxP15/15*^ exhibit tumours in the anterior lobe of the pituitary with clusters of nucleo-cytoplasmic β-catenin. Taken together, our results indicate that the Sox2+ve pituitary stem cells bearing bi-allelic loss of the tumour suppressor *Apc* (*Sox2*^*CreERT2/+*^*;Apc*^*LoxP15/15*^) are able to initiate aCP tumour formation, resulting in clusters of accumulating β-catenin that express diagnostic hallmarks of aCPs.

### *Apc*-driven cell clusters with nuclear β-catenin translocation undergo SASP and secrete inflammatory mediators, chemokines, cytokines, keratins and developmental growth factors

**Figure 7:**
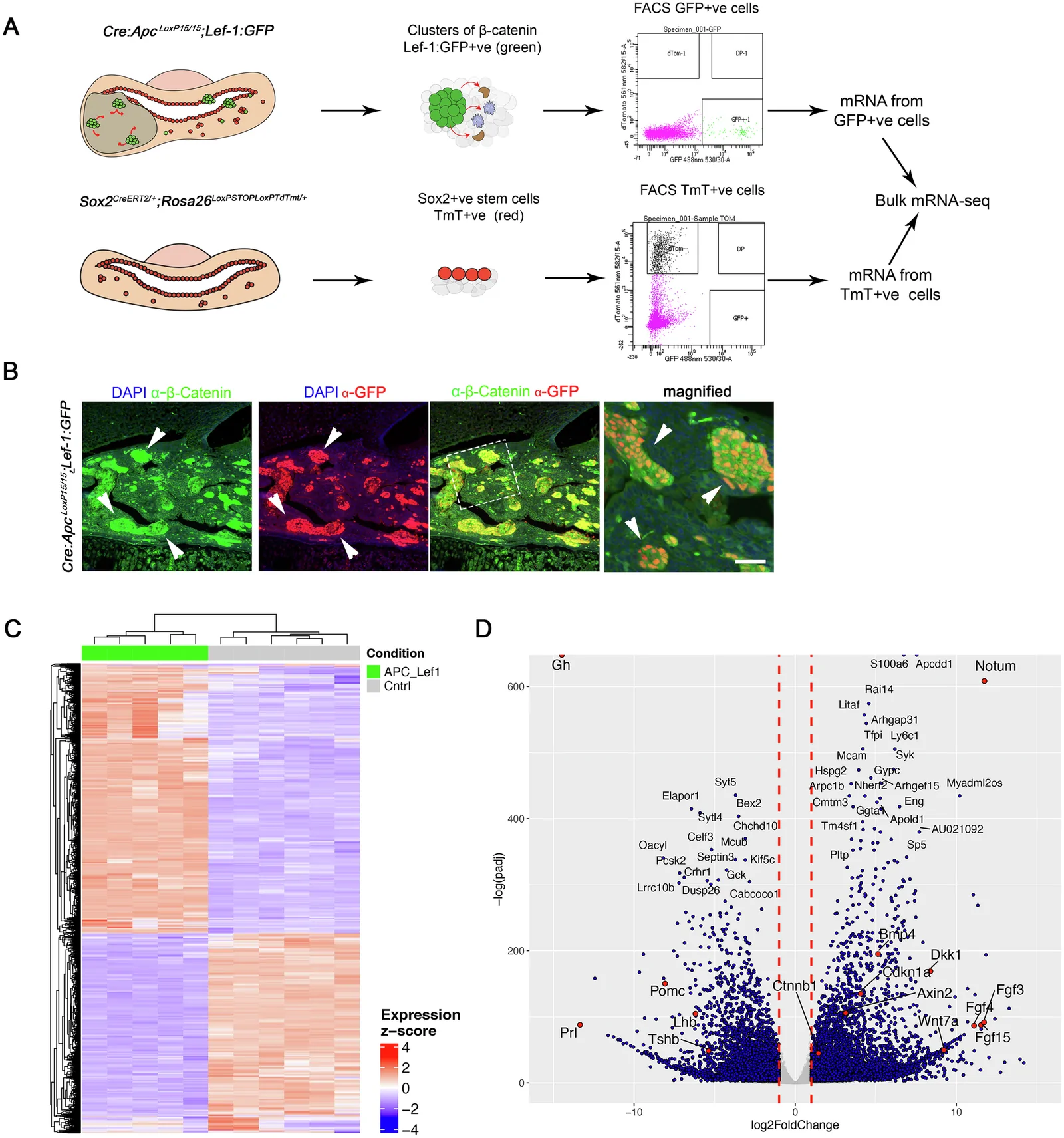
Transcriptomic analyses of early *Apc*-driven β-catenin+ve GFP+ve cell clusters show transcriptional signatures involved in inflammation, developmental growth factors and SASP. (**A**) Diagram representing experimental design: mRNA sequencing from β-catenin + ve clusters from *Apc*-driven tumours (*Cre:Apc*^*LoxP15/15*^*;Lef-1:GFP*) was performed from the FAC-sorted GFP+ve fraction cells (β-catenin + ve GFP+ve cell clusters) and compared to Sox2+ve stem cells from Tam-induced *Sox2*^*CreERT2/+*^*;Rosa26*^*TdTomato*^ (Tomato+ve fraction cells). (**B**) Double immunofluorescence against β-catenin (green) and α-GFP (red) revealed that all the nucleo-cytoplasmic β-catenin + ve cell clusters (white arrows) express GFP under the *Lef1* reporter sequence of the *Cre:Apc*^*LoxP15/15*^*;Lef-1:GFP*. (**C**) Hierarchical clustering of the top 5000 most variable genes separates the cell population’s transcriptome of the *Apc*-driven β-catenin + ve GFP+ve cell clusters from control *Sox2*^*CreERT2/+*^*;Rosa26*^*TdTomato/+*^. (**D**) Volcano plot showing differential gene expression between the β-catenin + ve GFP+ve cell clusters and control Sox2+ve pituitary stem cells. Highlighted in red are key upregulated genes such as the negative regulators of the Wnt/β-catenin pathway *Notum, Dkk1;* the downstream effectors of the activated Wnt/β-catenin pathway, such as *Axin2, Sp5, Wnt7a*, indicating overactivation of the canonical Wnt/β-catenin pathways*;* the developmental secreted factors *Fgf3*, *Fgf4*, *Fgf15* and *Bmp4* are found upregulated in addition to the senescence-associated factor *p21 (Cdkn1a)*. In line with aCPs being non-secreting tumours, pituitary terminal differentiation factors such as *Gh*, *Pomc*, *Lhb*, *Tsh* and *Prl* were downregulated. Differential expression analysis was performed using DESeq2; *P* values were calculated using a two-sided Wald test and adjusted for multiple comparisons using the Benjamini–Hochberg method (adjusted *P* < 0.05). *Gh* growth hormone, FACS Fluorescent-Activating Cell Sorting, *Lhb* luteinising hormone, Prl prolactin, Tsh thyroid-stimulating hormone. Scale bar in (**B**) represents 40 μm, and the images are representative of 4/5 mutant embryos. Source data are available online for this figure.

**Figure 8:**
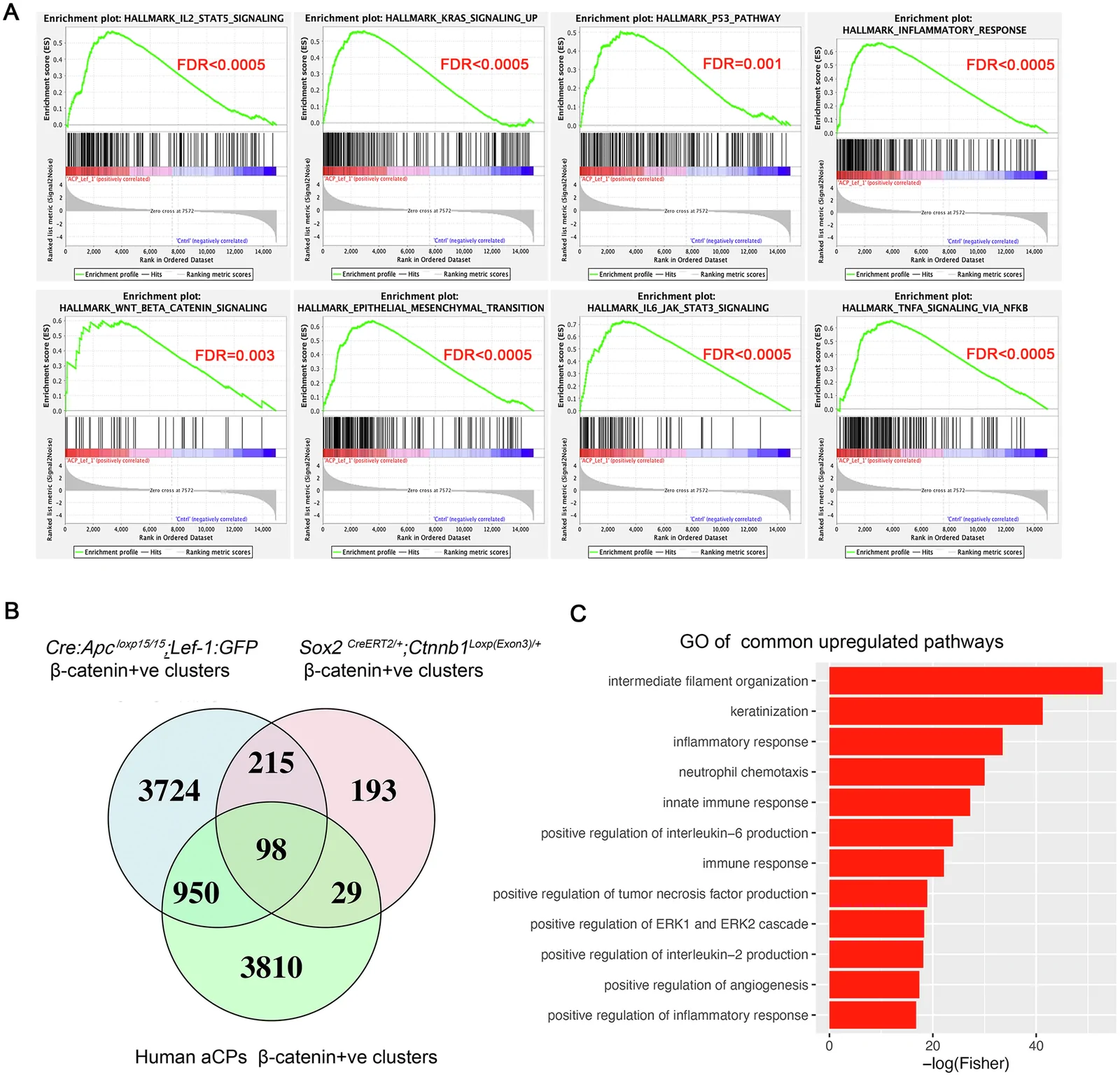
Transcriptomic and methylomic analyses shows that *Apc*-driven tumours exhibit shared similarities with murine and human β-catenin + ve aCPs. (**A**) Unsupervised Gene Set Enrichment analyses confirm that the β-catenin + ve GFP+ve cell clusters over-activate the Wnt/β-catenin canonical pathway with statistically significant upregulation of IL-2-Stat5, IL6-Stat3 and TNF-α signalling pathways involved in inflammatory response and SASP. The KRAS and p53 pathways were also found to be upregulated by the β-catenin + ve GFP+ve cell clusters, as were genes involved in epithelial-to-mesenchymal transition. (**B**) Venn diagram comparing differential gene expression between *Cre:Apc*^*LoxP15/15*^;*Lef-1:GFP* derived clusters and clusters from β-catenin-mutated+ve model (*Sox2*^*CreERT2/+*^*;Ctnnb1*^*Loxp(Exon3*)/+^) and from human aCPs reveals higher number of genes in common between human aCP clusters and *Apc*-driven β-catenin + ve GFP+ve clusters (950 genes in common). (**C**) GO enrichment indicates upregulated pathways in both human aCPs, β-catenin-mutated+ve clusters and our model. These upregulated transcriptional signatures are important in aCP development and include pathways involved with keratinisation, inflammasome:neutrophil chemotaxis, interleukin 6 and 2, angiogenesis, the innate immune response, positive regulation of inflammatory response and MAPK pathways.

β-catenin-mutated+ve aCPs are characterised by whorl-like clusters of accumulating nucleo-cytoplasmic β-catenin, which undergo SASP (Andoniadou et al, 2013; Gonzalez-Meljem et al, 2017) and act as hubs of secreting factors into the tumour microenvironment (Andoniadou et al, 2013; Apps et al, 2018; Gonzalez-Meljem et al, 2017). To investigate whether *Apc*-driven tumours contain clusters that act as signalling hubs, and to compare their molecular signatures to human β-catenin-mutated+ve aCPs, we performed transcriptomic analyses from isolated cell clusters from our *Cre:Apc*^*LoxP15/15*^ model at embryonic stages, aiming to identify early tumour transcriptional signatures. To isolate β-catenin+ve cell clusters, we utilised the transgenic line *Tcf/Lef-1:H2B-GFP* as a readout of the activated Wnt pathway (Ferrer-Vaquer et al, 2010). The *Tcf/Lef-1:H2B-GFP* transgenic line (hereafter referred to as *Lef-1:GFP* for simplicity) harbours a knock-in of the Wnt/β-catenin signalling reporter construct containing 6 *Tcf/Lef1* response elements that drive the expression of GFP, enabling fluorescent marking of Wnt-responsive cells. This transgenic line has been shown to accurately recapitulate the known pattern of Wnt-responsive cells and, therefore, represents an accurate read-out of the activated Wnt/β-catenin canonical pathway (Ferrer-Vaquer et al, 2010). We crossed our model *Cre:Apc*^*LoxP15/15*^ to the *Lef-1:GFP* reporter strain to generate *Cre:Apc*^*LoxP15/15*^*;Lef-1:GFP* compound mutants and assessed if the β-catenin+ve clusters expressed GFP (Fig. 7A,B). Double immunofluorescence against β-catenin and GFP revealed that all β-catenin+ve clusters, which over-activate Wnt-downstream targets, expressed GFP (Fig. 7B). Moreover, no GFP+ve cells were observed outside of these cell clusters, enabling accurate and specific fluorescent labelling of the β-catenin+ve cell clusters using GFP and the ability to perform purification using fluorescence activated cell sorting (FACS) (Fig. 7A). Tumours were dissociated to single cells and the GFP+ve fraction (β-catenin+ve GFP+ve cell clusters) was FAC-sorted before mRNA was extracted, purified and submitted for transcriptomic analyses. The mRNA from the GFP+ve cell fraction was compared to mRNA isolated from Wt pituitary stem cells from the *Sox2*^*CreERT2/+*^*;Rosa26*^*LoxPSTOPLoxPTdTmT*^ which allows for FAC-sorting of Sox2+ve cells expressing the red fluorescent protein TdTomato (or TdTmt for simplicity) (Fig. 7A). Hierarchical clustering of the most variable expressed genes distinguished transcriptional differences between the β-catenin+ve-GFP+ve cluster cells from that of the Sox2+ve TdTmt+ve cell population (Fig. 7C). Volcano plot analysis of differentially expressed genes revealed that *Apc*-driven clusters (β-catenin + ve GFP+ve) downregulate pituitary hormones such *Gh*, *Tsh*, *Pomc*, *Prl* and *Lh* (Fig. 7D) in accordance with the non-secreting nature of aCPs and is in keeping with our previous immunostaining analyses (Figs. 1B and 4D). Upregulated genes indicated that β-catenin+ve GFP+ve cell clusters activate the Wnt/β-catenin canonical pathway: with increase in the Wnt-downstream targets *Axin2*, *Lef1*, *Sp5*, *Tcf4*; and the secreted Wnt ligands: *Wnt7a*, *Wnt6*, *Wnt10a*, *Wnt9b*, *Wnt11*, *Wnt16* and their receptors and co-receptors *Fzd4*, *Fzd10*, *Lgr6*. Moreover, the secreted Wnt antagonists *Notum, Dkk1*, *Dkk2*, *Dkk4*, *Dkk3* and *Wif1* were found to be highly upregulated in the *Apc-*driven β-catenin+ve GFP+ve cell clusters (Appendix Fig. S4a). We identified that genes such as the secreted fibroblast growth factor (*Fgfs*): *Fgf3*, *Fgf15*, *Fgf4*, *Fgf8, Fgf20* and *Fgf17*, the bone morphogenic factors: *Bmp4, Bmp8a*, *Bmp2*, *Bmp5*, *Bmp15* and *Shh* were found highly upregulated (Appendix Fig. S4b,c). Keratins and keratin-associated proteins were highly upregulated by the *Apc*-driven β-catenin+ve GFP+ve clusters, which may contribute to the large deposits of wet keratin observed in our *Apc*-mutant model (Fig. 4E; Appendix Fig. S5a,b). We identified that the GFP+ve β-catenin+ve clusters secrete a large number of interleukins (*Il6,* *Ilb, Il2b, Il36b, Il10, Il16, Il15*) and chemokines (*Ccl2, Ccl3, Ccl4, Ccl5, Ccl7, Ccl11, Cxcl9, Cxcl15, Cxcl3, Cxcl1, Cxcl11*) which have been reported to have pro-inflammatory, chemotactic, immunosuppressor and pro-angiogenic functions during tumour development and are the main components of SASP (Liu et al, 2025; Meguro and Nakanishi, 2025; Ohtani, 2022; Schmitt et al, 2022) (Appendix Fig. S6a–c). In agreement with SASP, we identified that the SASP-associated factor p21 (*Cdkn1a*) was highly expressed in the *Apc*-driven β-catenin+ve GFP+ve cell clusters (Fig. 5D; Appendix Fig. S7). Some of the above-upregulated genes were found overexpressed in our *Apc*-model by in situ hybridisation in a cell cluster fashion (Fig. 5D), and mRNA-seq data were validated by RT-qPCR using mRNA isolated from *Apc-*driven GFP+ve β-catenin+ve cell clusters (Appendix Fig. S5c). Unsupervised gene set enrichment analyses (GSEA) identified pathways upregulated by the *Apc*-driven β-catenin+ve GFP+ve cell clusters, such as positive regulation of inflammation, enrichment of IL2-Stat5 and IL6-Jak-Stat3 signalling pathways. These pathways are important in immunomodulation, inflammation and angiogenesis and are key mediators of SASP (Meguro and Nakanishi, 2025; Ohtani, 2022; Schmitt et al, 2022) (Fig. 8A). Genes involved in positive regulation of TNF-alpha as well as KRAS and p53 pathways were found upregulated in cluster cells (Fig. 8A). To validate our transcriptomic results, we performed double immunofluorescence in our *Apc*-driven tumours (*Cre:Apc*^*LoxP15/15*^) with β-catenin and either p21, p16 and the senescence markers: phosphorylated γ-H2AX (DNA-damage associated protein), NF-Kβ (p65), regulator of SASP that drives expression of pro-inflammatory cytokines and the IL6-downstream signalling mediator pSTAT3 (Wang et al, 2024a). We identified that the *Apc*-driven β-catenin cell clusters express high levels of p21, with increased DNA-damage-associated protein γ-H2AX, NF-Kβ (p65) and pSTAT3, whilst they were negative for p16 in line with our transcriptomic analyses (Appendix Fig. S7).

We then compared our mRNA-seq transcriptomic data from our *Apc*-driven β-catenin+ve GFP+ve clusters to publicly available bulk mRNA-seq transcriptomics data sets, including human β-catenin-mutated+ve aCP bulk mRNA-seq data (Apps et al, 2018) and data from murine models that express a degradation-resistant form of β-catenin within Sox2+ve pituitary stem cells (*Sox2*^*CreERT2/+*^*;Ctnnb1*^*LoxP(Exon3)/+*^) (Gonzalez-Meljem et al, 2017) (Fig. 8B,C). Gene Ontology (GO) analyses identified common upregulated pathways between our murine *Apc*-driven model and human β-catenin-mutated+ve aCPs, such as keratinisation, with a high number of keratin and keratin-associated proteins found upregulated (Fig. 8C; Appendix Fig. S5a,b), inflammatory response, neutrophil chemotaxis, IL2, IL6 and positive regulators of angiogenesis. These pathways are all linked to SASP mechanisms known to occur in β-catenin-mutated+ve aCPs (Apps et al, 2018; Gonzalez-Meljem et al, 2017; Jiang et al, 2023; Prince et al, 2024). One distinction identified in our transcriptomic analyses is that the *Apc*-driven β-catenin+ve GFP+ve clusters do not upregulate the senescence-associated factor p16 (*Cdkn2a*), in contrast to the p16 upregulation reported in some human and murine β-catenin-mutated+ve aCP clusters (Apps et al, 2018; Gonzalez-Meljem et al, 2017).

We then studied the expression levels of p21 in cultured progenitor/stem cells derived from either *Cre:Apc*^*LoxP15/15*^ (severe embryonic tumours), *Cre:Apc*^*LoxP1-15/15*^ (postnatal tumours with milder phenotype) and *Cre:Apc*^*LoxP1-15/1-15*^ (only hyperplasia). Interestingly, cells from (*Cre:Apc*^*LoxP15/15*^) had significantly higher levels of p21 protein compared to *Cre:Apc*^*LoxP1-15/15*^ and to *Cre:Apc*^*LoxP1-15/1-15*^ (Fig. EV2D,E). Higher levels of p21 correlated with a greater size of β-catenin cell clusters (Fig. EV2A), suggesting a more robust senescence in the *Cre:Apc*^*LoxP15/15*^ compared to the other genotypes. These findings support the notion that *Apc-*driven clusters undergo p21-mediated senescence and suggest that the degree of tumour severity correlates with higher levels of p21 expression and size of the β-catenin cell clusters in our three allelic series of *Cre:Apc*^*LoxP15/15*^*, Cre:Apc*^*LoxP1-15/15*^ and *Cre:Apc*^*LoxP1-15/1-15*^. Together, our data show that although *Apc*-driven β-catenin+ve cell clusters are in some respects transcriptionally analogous to previously described β-catenin-mutated+ve aCP (Andoniadou et al, 2013; Gonzalez-Meljem et al, 2017), (Apps et al, 2018; Prince et al, 2024), they exhibit some expression differences.

### Murine aCP models recapitulate epigenomic changes of human aCPs and share epigenetic alterations of SASP-like inflammatory programme

**Figure EV3:**
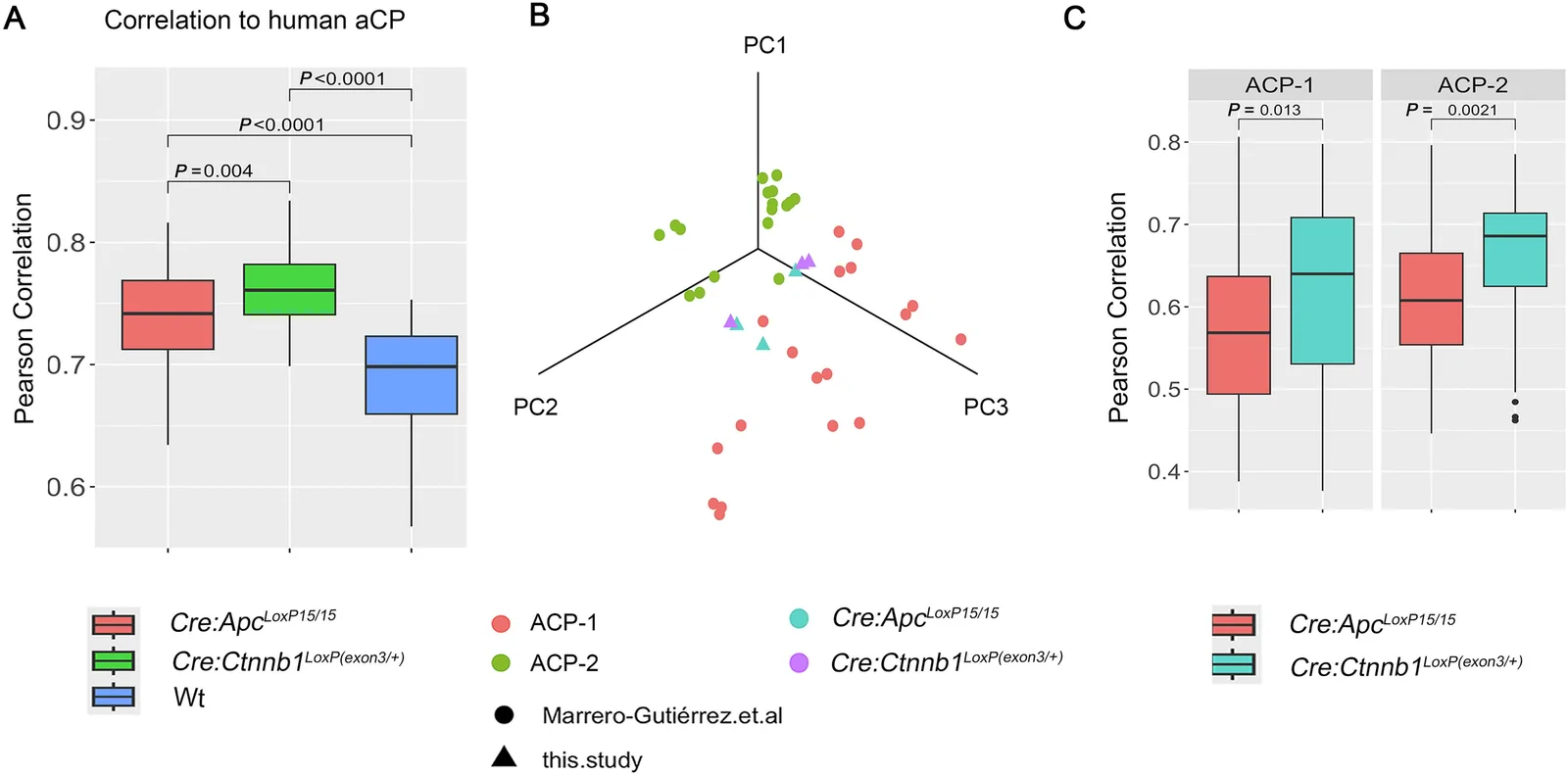
Methylation analyses among aCP genetic subtypes. (**A**) Boxplot showing Pearson Correlations between DNA samples from *Cre:Apc*^*LoxP15/15*^ or *Cre:Ctnnb1*^*LoxP(exon3)/+*^ tumours or Wt murine pituitaries compared to human aCPs samples from Capper et al, 2018 revealed correlation between murine *Acp* and *Ctnnb1.*^*.*^Reported *P* values calculated by Wilcoxon test. (**B**) Principal component analysis of DNA methylation samples from *Cre:Apc*^*LoxP15/15*^ or *Cre:Ctnnb1*^*LoxP(exon3)/+*^ tumours and Wt murine pituitaries alongside reference human samples from Marrero-Gutierrez, et al, 2024 study. (**C**) Boxplot showing Pearson correlations between DNA samples from and *Cre:ApcLoxP*^*15/15*^ or *Cre: Ctnnb1*^*LoxP(Exon3)/+*^ tumours and human samples split according to two reported methylation subtypes ACP *Ctnnb1* mutation status (right). Reported *P* values calculated by the Wilcoxon test. For boxplots in (**A**) and (**C**), the centre line represents the median (50th percentile), box bounds represent the 25th and 75th percentiles, and the lower and upper whiskers represent the minimum and maximum observed values, respectively, within 1.5× the interquartile range; values beyond the whiskers are plotted individually as outliers.

DNA methylation array profiling is now routinely used to aid human CNS tumour diagnoses (Capper et al, 2018; Sill et al, 2026), and its application to aCPs has recently identified two methylation subgroups, ACP-1 and ACP-2, with the latter enriched for *CTNNB1* mutations and associated with larger tumour size (Marrero-Gutierrez et al, 2024). To explore the degree to which murine aCP models recapitulate human tumour epigenomes, we used the Infinium Mouse Methylation array to profile Wt pituitaries alongside bulk tumours from *Cre:Apc*^*LoxP15/15*^ and *Cre:Ctnnb1*^*LoxP(exon3)/+*^ mice. Using orthologous probes between mouse and human arrays to facilitate cross-species analysis (Schoof et al, 2026; Zhou et al, 2022), we compared murine aCP models to data from human tumours and control pituitary tissue (Capper et al, 2018). This showed that Wt murine pituitary clustered alongside human control pituitary tissue, whereas *Cre;Ctnnb1*^*LoxP(exon3)/+*^ and, to a lesser extent the *Cre:Apc*^*LoxP15/15*^ tumours, clustered alongside human aCP tumour samples (Fig. 8D). Consistent with this finding, both *Cre:Apc*^*LoxP15/15*^ and *Cre:Ctnnb1*^*LoxP(exon3)/+*^ showed significantly higher mean Pearson correlations with human aCP references (Capper et al, 2018) when compared to Wt murine pituitary (Fig. EV3A).

Differential methylation analysis between *Cre:Ctnnb1*^*loxP(exon3)/+*^ and Wt pituitaries identified significant 1710 dmr-linked genes (Fig. 8E). While fewer total dmr-linked genes were detected in the *Cre:Apc*^*LoxP15/15*^ tumours, 106/122 (86.8%) were also detected in the *Cre;Ctnnb1*^*loxP(exon3)/+*^ tumours, indicating substantial shared epigenetic alterations between the two murine models. Interestingly, among the shared hypomethylated dmr-linked genes, there was *Il6*, consistent with its established role in aCP as a canonical SASP cytokine (Gonzalez-Meljem et al, 2017). Additional shared hypomethylated genes included *Parp1*, which is linked to DNA damage response and plays a role in the establishment of cellular senescence and the maintenance of the SASP phenotype (Kolacz and Robaszkiewicz, 2024). Similarly, we identified *Tlr4*, a key mediator of NF-κB signalling (Kim et al, 2023), which is known to regulate SASP-associated inflammatory responses (Acosta et al, 2013) and *Igfbp4*, a secreted growth factor induced by genotoxic stress with a role in SASP signalling (Alessio et al, 2020). Gene Ontology enrichment analysis of shared dmr-linked genes identified over-representation of inflammatory-related pathways, including nitric oxide biosynthesis, *IL-1β*, *IL-6* and *TNF-α* production (Fig. 8F). Interestingly, NF-κB activation drives inducible nitric oxide synthase (iNOS) expression and nitric oxide (NO) production, a known and secreted mediator of senescence (Bagheri et al, 2017).

We compared our murine tumours from the *Cre:Apc*^*LoxP15/15*^ and *Cre:Ctnnb1*^*LoxP(exon3)/+*^ models to the recently described human aCP DNA methylation subtypes (Marrero-Gutierrez et al, 2024). We show that the *Cre:Ctnnb1*^*LoxP(exon3)/+*^ tumours were significantly more correlated to both ACP-1 and -2 subtypes than those from *Cre:Apc*^*LoxP15/15*^; with a more prominent effect in the *CTNNB1*-enriched ACP-2 subtype. As expected, the *Cre:Ctnnb1*^*LoxP(exon3)/+*^ tumours were significantly more correlated to human tumours containing *CTNNB1* mutations than *Apc-*driven tumours because the *Cre:Ctnnb1*^*LoxP(exon3)/+*^ have the same underlying mutated driver as the ACP-2 subgroup (Fig. EV3B,C). Neither of the murine genotypes were associated with significant copy number alterations (CNVs), which is consistent with reports that CNVs are often absent in primary aCPs but can emerge at recurrence, consistent with genetic instability (Apps et al, 2024) (Appendix Fig. S8a).

Taken together, these data show that murine aCP models recapitulate epigenomic changes found in human tumours and support a model in which shared epigenetic alterations converge on a SASP-like inflammatory programme that is highly consistent with established aCP biology.

## Discussion

Previous genetic studies in mice (Andoniadou et al, 2013; Gaston-Massuet et al, 2011) and humans (Brastianos et al, 2014; Buslei et al, 2005; Sekine et al, 2002) have demonstrated that somatic mutations leading to a non-degradable form of β-catenin are the main driver of most aCPs. Recently, two studies presented an association between mutations in *APC* leading to FAP in three patients reported to carry germline truncated mutations in *APC* (Gorelyshev et al, 2020; Passos et al, 2020). A further association between aCPs and either FAP (Fodde et al, 2001), or *APC*-pathogenic syndromes such as Gardner (Gardner and Richards, 1953) and Turcot syndromes (Malbari and Lindsay, 2020) has been reported in 8 patients developing aCPs (Alvarez Salgado et al, 2017; Aquilina et al, 2006; Bose et al, 2024; Kim et al, 2014; Link et al, 2002; Powers et al, 2007; Rivera et al, 2011; Shah et al, 2007; Uemura et al, 2022; Yan et al, 2009). However, the lack of full genomic sequencing data for these patients’ tumours has not resolved whether mutations in *APC* had a direct role in the causation and formation of their aCPs. In this paper, we use three transgenic models to unequivocally show that disruption of *Apc* in the murine pituitary progenitor/stem cells is an alternative driver of aCPs without mutations in β-catenin, leading to activation of the Wnt/β-catenin pathway. Firstly, we show that bi-allelic loss of *Apc exon15 (Cre:Apc*^*LoxP15/15*^) leads to severe intrauterine aCP tumours, which are not compatible with postnatal life. Importantly, cases of severe intrauterine aCPs have been reported by both our group (Scagliotti et al, 2016) and others (Chentli et al, 2012; do Prado Aguiar et al, 2013), indicating that aCPs can present at any stage of life. The severity of the phenotype, with a lack of all hormone-producing cells, leads to early perinatal lethality in this model. We developed a second model by generating a hypomorphic allele for *Apc* in which Wt-*Apc* is still produced (*Cre:Apc*^*LoxP1-15/15*^), leading to 100% penetrant postnatal aCPs. In this *Apc*-hypomorphic allele, postnatal tumours develop that have all the histopathological and molecular hallmarks of human aCPs, including large deposits of wet keratin, which were not observed in previously published β-catenin-mutated+ve aCP murine models (Gaston-Massuet et al, 2011; Gonzalez-Meljem et al, 2017). Importantly, this new *Apc*-driven aCP model, faithfully recapitulates one of the most important aCP-disease co-morbidities, tumour-induced morbid obesity, which was not seen in the previous murine model of aCPs driven by oncogenic β-catenin (*Hesx1*^*Cre/+*^*;Ctnnb1*^*Loxp(Exon3)/+*^) (Gaston-Massuet et al, 2011; Gonzalez-Meljem et al, 2017). Thus, our model represents a robust genetic tool to study aCP tumour-induced hypothalamic obesity, a major complication seen in aCP patients (Apps et al, 2023; Erfurth and Muller, 2025; Muller, 2025; Muller et al, 2019; Muller et al, 2022). It is worth noting that in our *Apc*-driven aCPs (*Cre:Apc*^*LoxP1-15/15*^), we observed gender differences in tumour progression with females living longer than males. Whether this reflects a difference in tumour immune microenvironment between genders, as seen in models of *Wnt*-driven adrenocortical carcinoma (ACCs) (Wilmouth et al, 2022) will require further investigation. However, human epidemiological studies from large aCP patient registries do not show gender differences in disease progression for β-catenin-mutated+ve aCPs (Momin et al, 2021; Muller, 2014; Muller et al, 2019; Zacharia et al, 2012). Our mouse genetic study provides further information on the role of Apc in tumorigenesis and argues against a dominant negative effect of truncated *Apc*, which has been hypothesised in other cancers (Su et al, 1993; Zhang et al, 2016). We show three allelic series of pituitary-specific deletion of *Apc*, resulting in different phenotypic severity, which could be due to the degree of recombination leading to different amounts of Wt-Apc. Firstly, our *Cre:Apc*^*LoxP15/15*^ shows larger clusters of β-catenin that significantly express higher levels of p21, suggesting a more robust senescence compared to the hypomorphic model of aCPs (*Cre:Apc*^*LoxP1-15/1-15*^). Indeed, small amounts of Wt-Apc- expression have been shown to be sufficient to degrade β-catenin, resulting in a lower threshold of β-catenin-activity and to reduce tumour burden in colon adenoma (Buchert et al, 2010; Dow et al, 2015; Li et al, 2005). Genetic studies using mouse allelic series for *Apc* resulting in 10% expression of Wt-Apc were sufficient to ameliorate tumour progression, which aligns with our results (Albuquerque et al, 2002; Dow et al, 2015; Kielman et al, 2002; Su et al, 1993). Our data complement the above studies and point towards a threshold or “just-right signalling” levels of Apc resulting in different phenotypic outcomes (Clarke, 2006).

A previous report using a *Hesx1*^*Cre/+*^ driver to delete exon 14 of *Apc* (*Hesx1*^*Cre/+*^*;Apc*^*LoxP(exon14/14)*^) showed that in this genetic scenario, small clusters of nuclear β-catenin were formed, resulting in attenuated SASP, which was not sufficient to drive tumour formation (Gonzalez-Meljem et al, 2017). These experiments highlighted the importance of the level of SASP in aCP progression compared to the more robust, stronger SASP from the *Ctnnb1*-driven tumours. This previous model resembles our milder phenotype (*Cre:Apc*^*LoxP1-15/1-15*^), exhibiting small clusters of nuclear β-catenin and low levels of p21 expression, leading to hyperplasia of the pituitary gland instead of tumours. Using different Cre-driver and *Apc* alleles *Cre:Apc*^*LoxP15/15*^ and *Cre:Apc*^*LoxP1-15/15*^, we achieved larger clusters of nuclear β-catenin with stronger expression of p21-mediated senescence leading to robust SASP and to aCP tumour formation. Hence, our murine models, explain the causal effect of APC disruption reported in patients with FAP (Gabel et al, 2017; Passos et al, 2020) or APC-related syndromes (Alvarez Salgado et al, 2017; Aquilina et al, 2006; Bose et al, 2024; Kim et al, 2014; Link et al, 2002; Powers et al, 2007; Rivera et al, 2011; Shah et al, 2007; Uemura et al, 2022; Yan et al, 2009).

β-catenin-mutated+ve aCPs have been shown to originate from pituitary progenitor/stem cells that express the stem cell marker Sox2 (Andoniadou et al, 2013). Using a transgenic *Sox2*^*CreERT2/+*^ to delete *Apc* exon15, we show that bi-allelic loss of *Apc* exon15 in the Sox2+ve pituitary stem cells is sufficient to initiate tumour formation both embryonically and in adults, resulting in clusters of nucleo-cytoplasmic β-catenin that express early aCP markers, which further support a role of Apc disruption in the causation of aCPs. aCPs are characterised histologically by the presence of nucleo-cytoplasmic β-catenin+ve cell clusters that are a diagnostic hallmark of aCPs (Andoniadou et al, 2012; Buslei et al, 2005; Gaston-Massuet et al, 2011; Hofmann et al, 2006). These clusters have been shown to initiate tumour formation and have been studied extensively in β-catenin-mutated+ve aCPs both in mice (Andoniadou et al, 2012; Gaston-Massuet et al, 2011; Gonzalez-Meljem et al, 2017) and humans (Andoniadou et al, 2012; Apps et al, 2018; Wang et al, 2024b). These nucleo-cytoplasmic β-catenin+ve cell clusters exhibit SASP activity, secreting pro-inflammatory mediators, chemokines, cytokines and growth factors that collectively alter the tumour microenvironment. Our transcriptomic analyses of *Apc*-driven clusters revealed that, indeed, these tumour-initiating cells undergo SASP and act as secretory hubs to the tumour microenvironment. We identified key secreted SASP factors such as pro-inflammatory mediators, abundant cytokines and chemokines, interleukins and angiogenesis factors, all of which have been associated with SASP in various tumours (Meguro and Nakanishi, 2025; Ohtani, 2022; Schmitt et al, 2022). These clusters secrete numerous downstream targets of the Wnt canonical pathway and developmental growth factors such as Fgfs, Bmps and Shh, which may influence the tumour growth. Indeed, a recent study has shown that in β-catenin-mutated+ve aCPs, clusters can modify tumour-associated macrophages from a M1 (anti-tumourigenic) to M2 (pro-tumourigenic) phenotype in co-culture experiments (Wang et al, 2024b), an effect that could be similar within *Apc-*driven tumours. Although the *Apc-*driven β-catenin clusters seem largely analogous to their β-catenin-mutated+ve counterparts, some expression differences were noted that could confer specific targeted treatment. Compared to other previously published murine aCP models (Gaston-Massuet et al, 2011; Gonzalez-Meljem et al, 2017), we identify that the *Apc*-driven clusters express a large number of keratins and keratin-associated proteins which contribute to the large wet-keratin deposits that are parallel to human aCPs which were absent in other published aCP murine models. Another difference within our *Apc*-driven tumour model is the mechanism of SASP, which relies on the p21 pathway rather than *Cdkn2a* (p16), differing from previously published human and murine data on β-catenin-mutated+ve aCPs (Apps et al, 2018; Gonzalez-Meljem et al, 2017).

We provide novel epigenetic methylome data comparing our murine *Apc*+ve tumours to that of murine *Ctnnb1*+ve and human aCP *CTNNB1*+ve tumours, indicating some epigenetic similarities between tumour subtypes. These findings collectively point toward a convergent epigenetic programme that reinforces a senescence-associated inflammatory niche in aCP pathogenesis. Whether these transcriptional and epigenetic features are characteristic of all APC-mutated+ve tumours in humans requires additional studies in larger cohorts to enable stratification of transcriptional and epigenetic signatures based on the aCP-mutation-specific driver.

Our data call for in-depth genomic studies to identify the overall prevalence of *APC*-mutations in larger cohorts of aCP patients. These studies will lead to genotype/clinical phenotype correlation in patients without mutations in oncogenic β-catenin and identify whether their clinical trajectories differ i.e. number of recurrences and/or hypothalamic involvement. Importantly, our murine genetic studies show that dysfunction of *Apc* is a driver of aCPs, leading to activation of the Wnt/β-catenin pathway and hence *APC*-mutated+ve tumours represent a novel genetic subtype of aCPs. Since our data demonstrate the causal effect of *Apc*-dysfunction in the development of aCPs, this highlights the need for genetic testing and increased surveillance of patients with FAP or germline *APC*-pathogenic syndromes for the development of aCPs early in life. Furthermore, it warrants the inclusion of aCPs as part of the broader spectrum of tumours associated with *APC*-syndromic conditions. Equally, patients who develop aCPs with no mutations in β-catenin should be tested for underlying mutations in *APC* as a possible cause of FAP or APC-related syndromes such as Gardner and Turcot syndromes.

## Methods

**Table:** Reagents and tools table

Reagent/resource	Reference or source	Identifier or catalogue number
**Experimental models**		
*Rosa26* ^*CAGLoxpSTOPLoxpTdTomato*^	Jax-Lab	stock #007905
*Apc*Loxp^*(Exon15)/Loxp(Exon15)*^	Jax-Lab	stock #029275
*Apc* ^*Loxp(Exon1-15)/Loxp(Exon1-15)*^	Jax-Lab	stock #009045
*Tcf/Lef-1:H2B-GFP*	Jax-Lab	stock #032838
*Sox2* ^*CreERT2/+*^	Jax-Lab	stock #017593
*Ctnnb1* ^*LoxP(exon3)/+*^	M Taketo (Harada, N. et al, 1999)	Provided by M Taketo (Harada, N. et al, 1999)
**Antibodies**		
Rabbit α-phosphorylated-histone H3 (α-pHH3)	1:500 dilution, Millipore, 06-570	Millipore 06-570
Rabbit α-PRL	1:500 dilution	The National Hormone and Peptide Program (NHPP), Harbour-UCLA Medical Centre
Rabbit α-LH	1:500 dilution	The National Hormone and Peptide Program (NHPP), Harbour-UCLA Medical Centre
Rabbit α-TSH	1:500 dilution	The National Hormone and Peptide Program (NHPP), Harbour-UCLA Medical Centre
Rabbit α-GH	1:500 dilution	The National Hormone and Peptide Program (NHPP), Harbour-UCLA Medical Centre
Rabbit α-Pomc	1:500 dilution	The National Hormone and Peptide Program (NHPP), Harbour-UCLA Medical Centre
Rabbit α-GSU	1:500 dilution	The National Hormone and Peptide Program (NHPP), Harbour-UCLA Medical Centre
Goat α-Sox2	1:200 dilution, Statech, GT15098	Statech, GT15098
Rabbit α-Sox9	1:500 dilution	Millipore (AB5535)
Mouse α-β-Catenin	1:300 dilution, SIGMA, C7082	SIGMA (C7082)
Chicken α-GFP	1:100 Abcam (ab13970)	Abcam (ab13970)
Mouse α-APC CT (clone C-APC 28.9)	1:1000 Millipore (MAB3786)	Millipore (MAB3786)
Rabbit α-p21 [EPR18021]	1:1000 (Abcam) ab188224	Abcam (ab188224)
Rabbit α-pSTAT3	1:300 Cell Signalling	Cell Signalling (9145)
Rabbit α-NF-kB (p65)	1:300 (Abcam) ab16502	Abcam (ab16502)
Rabbit α-β-Catenin	1:300 (Abcam)	Abcam (ab16051)
Rat α-CDKN2A/p16INK4a antibody [PABLO33B])	1:300 (Abcam) ab241543	Abcam (ab241543)
Mouse α-H2A.X phosphor (Ser 139)	1:150 Biolegend G13402	Biolegend (G13402)
Mouse α-β-Actin	1:5000 Merck A5441	Merck (A5441)
Rabbit α-Vinculin	1:1000 dilution	Abcam (ab129002)
Biotinylated goat anti-rabbit antibody	1:300 dilution, Vector Laboratories, BA-1000	Vector Laboratories (BA-1000)
Biotinylated horse anti-goat antibody	1:200 dilution, Vector Laboratories, BA-9500	Vector Laboratories (BA-9500)
Donkey anti-goat antibody, Alexa Fluor 568	1:300, Invitrogen, A-11057	Invitrogen (A-11057)
Goat anti-mouse antibody, Alexa Fluor 488	1:300, Invitrogen, A-11001	Invitrogen (A-11001)
Goat-anti-mouse IRDye 800CW	(1:1000) 926-32210 LI-COR	926-32210 LI-COR
Goat-anti-Rabbit IRDye 680RD	(1:1000) 926-68071 LI-COR	926-68071 LI-COR
**Oligonucleotides and other sequence-based reagents**		
*Apc (exon 15)*	FW: ACGAAGACCACAGGCAAATC REV: CCACAAAGTTCCACATGCATTAC	Merck
*Axin2*	FW: ACGAAGACCACAGGCAAATC REV: CCACAAAGTTCCACATGCATTAC	Merck
*Lef1*	FW: ACTGTCAGGCGACACTTCCATG REV: GTGCTCCTGTTTGACCTGAGGT	Merck
*Wnt7a*	FW: TTCGCCAAGGTCTTCGTGGATG REV: TACAGGAGCCTGACACACCATG	Merck
*Ccl2*	FW: TTAAAAACCTGGATCGGAACCAA REV: GTCTGGACCCATTCCTTCTTGG	Merck
*Fgf3*	FW: GCAAGCTCTACTGCGCTACCAA REV: CACTTCCACCGCAGTAATCTCC	Merck
*Ccl3*	FW: TGTACCATGACACTCTGCAAC REV: CAACGATGAATTGGCGTGGAA	Merck
*Notum*	FW: CGTGGTACACTCAAGGATGTGC REV: GCCTTATGGCTGTCATGGAAGC	Merck
*Gh*	FW: TCAGAATGCCCAGGCTGCTTTC REV: ACTGGATGAGCAGCAGCGAGAA	Merck
*Tsh*	FW: GAGAGTGTGCCTACTGCCTG REV: ACAGACATCCTGAGAGAGTGC	Merck
*Prl*	FW: CTCAGGCCATCTTGGAGAAG REV: TCGGAGAGAAGTCTGGCAGT	Merck
*p21 (Cdkn1a)*	FW: GTCGCTGTCTTGCACTCTGG RE: CTCTTGCAGAAGACCAATCTGC	Merck
**Software**		
R version 4.3.0 (2023-04-21)	https://www.r-project.org	NA
GraphPad PRISM 11.0.1 (99)		GraphPad PRISM
Photoshop 2025		Adobe
**Other**		

### Animals

All experiments were conducted under regulations, licences and local ethical review (QM-AWERB Ethical Committee) following the UK Home Office Animals (Scientific Procedures) Act 1986. The transgenic lines *Rosa26*^*CAGLoxpSTOPLoxpTdTomato*^ (stock #007905) (Madisen et al, 2010), *Apc*^*Loxp(Exon15)/Loxp(Exon15)*^ (stock #029275) (Robanus-Maandag et al, 2010), *Apc*^*Loxp(Exon1-15)/Loxp(Exon1-15)*^ (stock #009045) (Cheung et al, 2010), *Tcf/Lef-1:H2B-GFP* (stock #032838) (Ferrer-Vaquer et al, 2010) and the *Sox2*^*CreERT2/+*^ (stock #017593) (Arnold et al, 2011) were obtained from the JAX-lab and have been previously described. The *Ctnnb1*^*LoxP(exon3)/+*^ was provided by M.Taketo (Gaston-Massuet et al, 2011; Harada et al, 1999). The *Prop1:Cre* transgenic line (Davis et al, 2016; Gualtieri et al, 2021) was kindly provided by Shannon Davis and Sally Camper. Animals were kept in 12 h light/12 h dark cycle, with a constant supply of food and water, temperatures of 65–75 °F ( ~ 18–23 °C) with 40–60% humidity. All animals were in a C57B6 genetic background. Mice from different litters and cages were randomly distributed across experimental groups to reduce potential confounding effects. Whenever possible, genotypes were kept blinded. Kaplan–Meier survival curves were plotted using humane endpoints as determined in line with UK Home Office regulations regarding the use of mice in research. Mice were humanely culled when health deterioration was assessed to be irreversible. Tamoxifen induction of the Cre recombinase in (*Sox2*^*CreERT2/+*^;*Apc*^*LoxP15/15*^) was achieved by 3 consecutive daily injections of 0.1 mg/g of mouse at e12, e13, e14 in embryos or two consecutive days for postnatal studies.

### Immunohistochemistry, immunofluorescence, and in situ hybridisation

Immunostaining was performed as previously described (Gaston-Massuet et al, 2008; Gaston-Massuet et al, 2016; Rai et al, 2022). In short, sections were deparaffinised, followed by rehydration through decreasing ethanol dilutions. Heat-induced antigen retrieval was performed with a microwave in 10 mM sodium citrate buffer (pH 6). Samples were left to cool and incubated for 1 h in blocking buffer [1X PBS, 0.1% Triton X-100, 5% Normal Goat Serum (Vector Laboratories)]. Antibodies, their concentration and source are listed in the reagents and tools table. Immunohistochemistry (IHC) staining was achieved using DAB Peroxidase Substrate Kit (Vector Laboratories; SK-4100). The colorimetric reaction was stopped with water, and sections were counterstained using haematoxylin (Sigma-Aldrich). For immunofluorescence (IF), conjugated secondary antibodies Alexa Fluor 568 or 488 were used, or a biotinylated secondary followed by streptavidin (Appendix Table S1). Sections were mounted with VECTASHIELD containing DAPI (Vector Laboratories). Images were acquired with a Leica microscope. Figures were generated with Adobe Photoshop CS9. The mitotic index (MI) is the percentage of pHH3-positive cells compared to the total number of cells (average counts from three different sections, separated approximately by 100 μm, per embryo/pituitary with a minimum of *n*  =  3–4 per genotype and stage). In situ hybridisation was performed by adapting the protocol from (Moorman et al, 2001) and described before in (Gaston-Massuet et al, 2005; Gaston-Massuet et al, 2016; Giri et al, 2017; Moorman et al, 2001). In short, slides were deparaffinised, rehydrated and fixed with 4% PFA. Slides were incubated with proteinase K, followed by a second fixation with 4% PFA and finally incubated with 0.1 M triethanolamine and 0.1% acetic anhydride (Sigma). Hybridisation was achieved by an overnight incubation with 100 ng of the digoxigenin-labelled probe at 65 °C. Sections were washed in 0.1 M Tris-HCl Buffer (pH = 7.5) followed by an overnight incubation at 4 °C with anti-Dig antibody (Sigma-Aldrich). Signal detection was achieved by colorimetric reaction using 4-nitro blue tetrazolium chloride solution (NBT; Sigma-Aldrich) and 5-bromo-4-chloro-3-indolyl phosphate disodium salt (BCIP; Sigma-Aldrich). The digoxigenin-labelled antisense probes *p21*, *Axin2*, *Shh*, *Fgf8*, *Fgf3*, *Wnt6* and *Wnt7a* were generated from plasmids containing either a portion or full-length cDNA of each respective gene, and plasmids were obtained from Source Bioscience, or gifts from Sally Camper, Andreas Kispert and Peter Gruss and published before (Potok et al, 2008; Trowe et al, 2013; Walther et al, 1991). Pituitary volume quantification was performed using H&E-stained pituitaries. Four sections in every 16 were counted for estimation. Microscope images were taken at ×5 magnification and analysed using ImageJ software. Estimation of pituitary volume was calculated by multiplying the area of the pituitary by the thickness of each section (7 µm) and correcting for the remaining sections in the series [Pituitary volume (µm^3^) = Measured area(µm^2^) × 0.7(µm) × 4]. Comparative analyses of pituitary volumes at each embryonic stage from at least 3 different embryos per genotype from different litters were made using Student’s *T* test.

### Fluorescent activated cell sorting (FACS)

To FAC-sort pituitary cell populations, pituitary single cell suspension was achieved by enzymatic treatment using 250 µl enzyme mix [0.05% Collagenase Type II (LorneLaboratories Ltd), 50 µg/ml DNaseI (Worthington), 2.5 μg/ml Amphotericin B (Gibco), 0.1% v/v trypsin-EDTA solution 0.05% (Gibco) in Hank’s Balanced Salt Solution (HBSS, Gibco)] in an RNase and DNsae-free 1.5-ml microcentrifuge tube (Axygen). Samples were incubated for 1 h at 37 °C under constant agitation at 500 rpm. Pituitaries were mechanically dissociated by repeated pipetting. Dead cells were stained with 500 µL of 1/5000 sterile DAPI solution, incubated under gentle agitation for 3 min. Samples were centrifuged, and cells were resuspended in 500 µL FACS buffer [25 mM HEPES (Gibco), 1% FCS (Sigma-Aldrich) in sterile PBS] by repeated pipetting and transferred to a flow cytometry tube. For mRNA purification, cells were FAC-sorted directly in 750 µL of TRIzolTM-LS (Invitrogen). Cells were sorted using a BD FACS Aria II sorter (BD Bioscience) using a 100-µm nozzle. Side scatter (SSCr) area and forward scatter (FSC) area were compared to ensure cellular matter was identified and sorted. SSCr width and FSC area were then compared to ensure only single cells were sorted. A 405 nm laser was used to sort DAPI+ve and DAPI-ve cells. A 561 nm laser was used to sort tdTomato+ve and tdTomato-ve cell populations. A 488 nm laser was used to sort GFP+ve and GFP-ve cells. Sorted cells were immediately snap frozen and stored at –80 °C until mRNA purification.

### Protein extraction and western blotting

Lysates of cultures from pituitary progenitors/stem cells from different genotypes were grown to 80% confluence, rinsed with ice-cold 1× PBS, and lysed in 200 μL lysis buffer (7 M urea, 10% glycerol [v/v], 10 mM Tris–HCl, pH 6.8, 1% SDS, 1 mM dithiothreitol) supplemented with protease/phosphatase inhibitors (Thermo Fisher Scientific). Lysis was enhanced by sonication (2 × 15 s) and passage through a 21 G needle (6×), followed by rotation for 30 min at 4 °C. Lysates were clarified by centrifugation (13,000×*g*, 15 min, 4 °C), and supernatants were collected. Protein concentrations were determined by BCA assay (Thermo Fisher Scientific) and normalised using lysis buffer. Samples were mixed with 6× Laemmli buffer (Thermo Fisher Scientific), heated at 95 °C for 5 min, and stored at −80 °C. Equal amounts of protein, alongside a molecular weight marker (Thermo Fisher Scientific), were separated on pre-cast polyacrylamide gels (NuPAGE 4–12% Bis-Tris, Novex, Invitrogen) at 200 V for 30–45 min. Proteins were transferred to PVDF membranes (Invitrogen) using a semi-dry system (Bio-Rad Trans-Blot) in transfer buffer (48 mM Tris base, 390 mM glycine, 0.1% SDS, 20% methanol [v/v]) at 15 V for 1 h. Membranes were rinsed in TBST, blocked in 5% (w/v) milk in TBST for 1 h at room temperature, and incubated overnight at 4 °C with primary antibodies diluted in blocking buffer (see Supplementary Table 2). After washing, membranes were incubated for 1 h at room temperature with IRDye 680CW or 800CW secondary antibodies (LI-COR; 1:5000 in blocking buffer). Signals were detected using a LI-COR imaging system. Band intensities were quantified using ImageJ software densitometric analyses. p21 expression levels were normalised to β-actin, and data represented as relative expression.

### RNA purification, RT-qPCR for recombination efficiency and validation of mRNA-seq

RNA from FAC-sorted cells from *Sox2*^*CreERT2/+*^*;Rosa26*^*LoxpSTOPLoxPtdTmT*^, *Cre:Apc*^*LoxP15/15*^;*Lef-1:GFP;Rosa26*^*LoxPSTOPLoxPtdTmT*^ or stem cell cultures from *Cre:Apc*^*15/15*^ or Wt pituitaries was obtained using PureLink^TM^ RNA Micro Kit (Invitrogen) following the manufacturer’s protocols. Samples were submitted to quality control using an Agilent 2100 Bioanalyser, and only samples with RNA integrity values ≥ 8.1 were kept for mRNA-seq analyses. To generate cDNA for RT-qPCR, 500 ng of RNA was incubated at room temperature for 15 min with 1 µL of DNAseI (Invitrogen), 1 µL 10x DNaseI Reaction Buffer and UltraPure RNAse-free water (Invitrogen) to a total volume of 10 µL. Reverse Transcription was performed using a High-Capacity cDNA Reverse Transcription Kit (Applied Biosystems) to produce cDNA according to the manufacturer's protocol. Quantity and quality of the cDNA samples were analysed using a Nanodrop ND-1000 spectrophotometer (Nanodrop), and the quality of cDNA was assessed by determination of the 260/280 and 260/230 ratios. Gene expression levels were analysed by RT-qPCR using QuantiTect SYBR Green PCR Kit (Qiagen) according to the manufacturer’s protocol and analysed with Stratagene (Agilent Technologies). A comparative Ct method (2^−ΔΔCT^ method) was used to compare the mRNA expression levels of genes of interest between cell populations, normalised to GAPDH. RT-qPCR primers for *GAPDH*, *Apc exon15, Axin2, Wnt7a, Notum, Fgf3, Shh, Il6 Gh, Tsh, Pomc*, *p21* and *Prl* are shown in.

### Pituitary stem cells (PSCs) culture and proliferation assays

PSCs were cultured from Wt or *Cre:Apc*^*loxP15/1-15*^ mutant pituitaries and incubated for 2 h in enzyme mix [0.5% w/v Collagenase, 50 µg/ml DNase, 2.5 µg/ml Fungizone, trypsin 0.1% in Hank’s Balanced Salt Solution] and mechanically dissociated into single cells. In all, 10,000 cells/well were plated in 12-well plates and cultured in stem cell-promoting media [Ultraculture Medium (Lonza), supplemented with 5% FCS (Sigma), 1% penicillin/streptomycin (P/S: Fisher), 1% glutamax (Fisher), 20 ng/ml basic fibroblast growth factor (R&D) and 50 ng/ml cholera toxin (Sigma)] (Gaston-Massuet et al, 2016). Media was changed every 48 h and cultures were maintained for 8 days. Cells were stained with Coomassie blue (Fisher), and either the number of colonies or cells per colony was counted.

### Statistics and reproducibility

Statistical analyses were performed using Prism9/10 software (GraphPad). The number of independent experiments and replicates (*n*) is indicated in each of the figure legends. Unless stated otherwise, at least three biologically independent replicates were performed for each panel, which came from at least three independent experiments. Each statistical test and the number of experimental animals (*n*)/repeats are reported in each figure legend. When appropriate, normalisation of the data was performed within each independent experiment. The number of experimental animals was minimised using estimation of sample size and power test calculations using two-sample inference -estimation with a α = 0.05 and power of 0.90. To minimise bias effect, genotypes were blinded to the researcher. No exclusion or inclusion criteria were applied in any of the experiments.

### mRNA-seq and bioinformatic analyses

For library preparation, mRNA enrichment for cell samples using the SMARTer ultralow input kit (Illumina), and 150 bp paired-end sequencing was then performed on a NovaSeq6000 platform. Reads were quality trimmed using Trimgalore v0.6.5 and aligned to the reference genome GRCm39 using STAR v2.7.9a (Dobin et al, 2013). Reads were counted using feature counts, and the ENSEMBL annotation gencode.vM33.annotation.gtf. Downstream analysis was performed in DESeq2 (Love et al, 2014), with filtering of genes with less than one read count per million (cpm). Genes with a |logFC | >2 and *P*.adj <0.05 were considered significant. Gene Ontology (GO) enrichment was calculated on the most strongly upregulated genes (logFC>5) and downregulated (logFC < –5) genes separately using the TopGO v2.52.0 package. For unsupervised hierarchical clustering, the top 5000 variable genes (ranked by standard deviation) were used. For comparison to the BcatGOF model (Andoniadou et al, 2013) and human aCP (Apps et al, 2018) published data raw fastqs were accessed from the European Nucleotide Archive (PRJEB18056) and analysed as above using GRCh38.release109 and ENSEMBL annotation Homo_sapiens.GRCh38.109.gtf for the human samples.

### DNA methylation analysis

To obtain sufficient genomic DNA, isolation was performed using the Quick-DNA/RNA micro-prep plus kit from ZYMO using three samples per genotype, consisting of pools of two tumours per sample. DNA methylation was assessed using Illumina’s Infinium Mouse Methylation BeadChip at the UCL Genomics, Institute of Child Health. Microarray data were analysed using a cross-package workflow in R, using minfi (Aryee et al, 2014), IlluminaMouseMethylationanno.12.v1.mm10, and DMRcate (Peters et al, 2015). Briefly, raw data were imported in R using minfi and quality control was performed by assessing the mean detection *P* values of all CpGs. Normalisation was performed using minfi’s preprocess Quantile method. Sample sex was determined using minfi’s getSex() function (7 males:2 females). Since not all experimental groups harboured both sexes, ComBat() from the sva package (Leek et al, 2012) was used to generate normalised beta-values with sex-linked variation removed as a batch effect. Differential methylation analysis was performed on the normalised matrix using the R packages limma and DMRcate (Peters et al, 2015), with an FDR <0.1 for cpg.annotate() and default parameters for dmrcate(). Thresholds of beta-value mean diff >|0.05| and *Stouffer* < 0.05 were used to define significant DMRs. Gene Ontology term enrichment for shared differentially methylated genes in *Cre:Apc*^*LoxP15/15*^ and *Cre:Ctnnb1*^*LoxP(exon3)*/+^ tumour was calculated in TopGO using a Fisher’s exact test. Copy number alterations were assessed using conumee2 (Daenekas et al, 2024), with a cohort of 11 Wt murine foetal brain samples as a reference (GEO: GSE184410 (Zhou et al, 2022)). For cross-species reference comparison to human samples, ortholog CpGs to the human EPIC array were selected (described at https://zwdzwd.github.io/InfiniumAnnotation). Control pituitary and aCP human samples were accessed from the processed data of the Heidelberg cohort (GEO: GSE109381 (Capper et al, 2018)), and the top 3000 variable probes (by standard deviation) were selected for principal component and Pearson correlation analysis alongside murine samples. For comparison to human adamanitomnatous craniopharyngioma DNA methylation subtypes, ACP-1 and ACP-2 (Marrero-Gutierrez et al, 2024), raw data were accessed (GEO: GSE239695), loaded, and quantile normalised with minfi. The top 5000 variable probes (by standard deviation) in the human references were selected for principal component and Pearson correlation analysis alongside murine samples. For both reference comparisons ComBat() from the sva package (Leek et al, 2012), was used to batch correct between mouse vs human samples.

## Supplementary information

Appendix

Peer Review File

Source data Fig. 1

Source data Fig. 2

Source data Fig. 3

Source data Fig. 5

Source data Fig. 4

Source data Fig. 6

Source data Fig. 7

Expanded View Figures

## Data Availability

The authors declare that all the data supporting the findings herein are included in the article (or Supplementary materials) and available from the corresponding author (CGM) upon reasonable request. All raw and processed sequencing data files have been deposited to GEO under reference GSE282683 (RNA) and GSE328638 (DNA methylation) with the following secure token: mdczogccxtitfgh, to allow review while it remains under private status. The source data underlying graphs and supplementary materials are provided as a Source Data file. A reporting summary for this article is available as a Supplementary Information file. The source data of this paper are collected in the following database record: biostudies:S-SCDT-10_1038-S44321-026-00525-2.
