# Supplementary material for: Apc-deficient pituitary stem cells drive adamantinomatous craniopharyngioma through senescence-associated inflammation
Source: EMBO Mol Med. 2026 Oct 9;18(11):4386–412. doi: 10.1038/s44321-026-00525-2 (PMC13748607; doi:10.1038/s44321-026-00525-2)
Supplement: Supplementary file 1 [file 44321_2026_525_MOESM10_ESM.pdf]

Expanded View Figures

Figure EV1. Histopathological features across aCP tumour subtypes.

High-magnification images of tumours from embryonic *Cre:Apc*$^{LoxP15/15}$ (**A**), murine embryonic *Ctnnb1*-driven tumours (*Cre:Ctnnb1*$^{LoxP(exon3)/+}$) (**B**) and human *Ctnnb1+ve* aCP (**C**) reveal common histological diagnostic hallmarks of aCPs. (**A**) High magnification of H&E (from Fig. 1B) or IHC (against β-catenin) of sections from embryonic *Cre:Apc*$^{Loxp15/15}$ tumours revealed the presence of classical histopathological features of aCPs, such as whorl-like cell clusters (arrows) and palisading epithelium (arrowheads) that form finger-like protrusions. Consecutive sections stained against β-catenin revealed that these whorl-like cell clusters express nuclear β-catenin (arrows), which is a diagnostic marker for aCPs. Likewise, these histological features were present in sections from murine *Ctnnb1*+ve driven tumours (**B**) (*Cre:Ctnnb1*$^{LoxP(exon3)/+}$) with whorl-like clusters of cells (arrows) which express nuclear β-catenin (arrows) and form finger-like protrusions. (**C**) Representative section of a *CTNNB1+ve* human aCP stained with H&E or IHC against β-catenin exhibiting the classical histological diagnostic features of aCP, such as: whorl-like clusters of cells (arrows), palisading epithelium (arrowheads), deposits of wet keratin (wk) (which are also present in our mid-stage Apc-tumours from our murine *Cre:Apc*$^{Loxp1\text{-}15/15}$ Fig. 4E). These tumours present clusters of accumulating β-catenin (arrows). Images are representative of 4 tumours per genotype. H&E haematoxylin and eosin, IHC immunohistochemistry, wk wet keratin. Scale bars represent 50 µm.

© The Author(s)

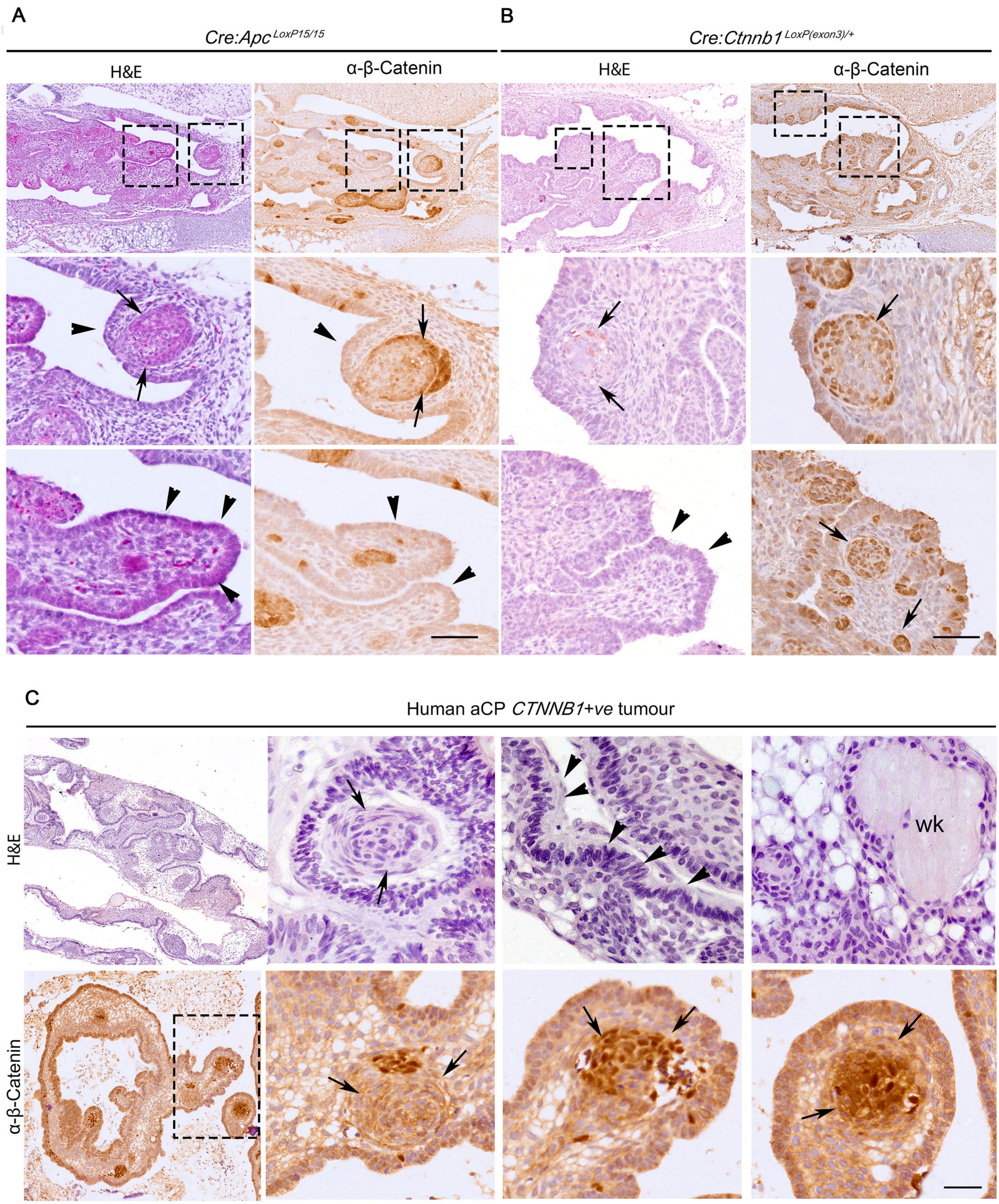
A
Cre:Apc LoxP15/15
H&E
α-β-Catenin
B
Cre:Ctnnb1 LoxP(exon3)/+
H&E
α-β-Catenin
C
Human aCP CTNNB1+ve tumour
H&E
wk
α-β-Catenin

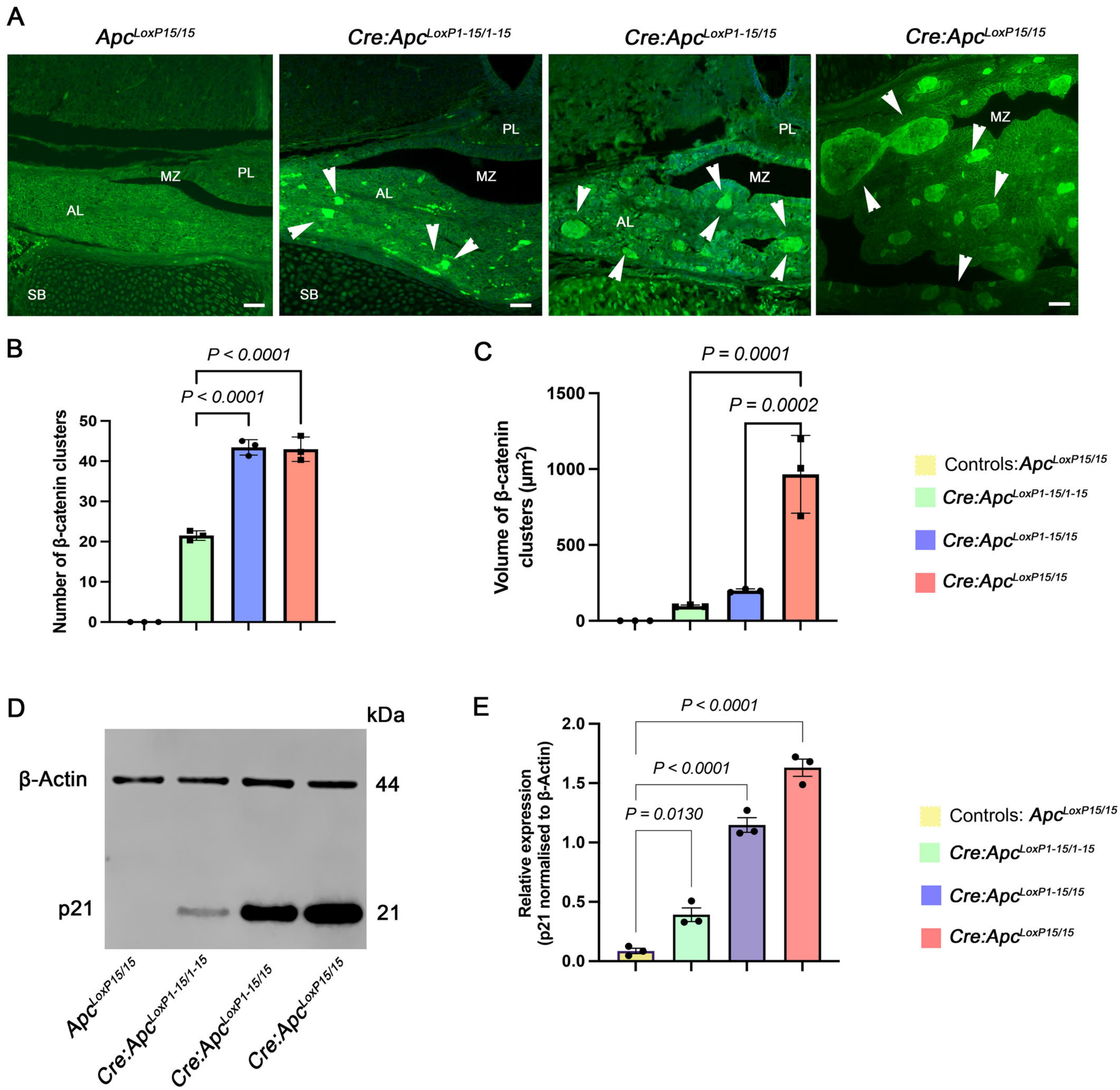

Figure EV2. The *Cre;Apc*$^{LoxP15/15}$ mutant pituitaries exhibit larger nucleo-cytoplasmic β-catenin accumulating cell clusters and express higher levels of the senescence marker p21 compared to the hypomorph alleles *Cre;Apc*$^{LoxP1-15/15}$, *Cre;Apc*$^{LoxP1-15/1-15}$.

(**A**) Immunofluorescence against β-catenin (green) on coronal sections through the pituitary glands reveals that the *Cre;Apc*$^{LoxP15/15}$ mice have larger nucleo-cytoplasmic β-catenin cell clusters compared to the hypomorph allele *Cre;Apc*$^{LoxP1-15/15}$. The *Cre;Apc*$^{LoxP1-15/1-15}$ exhibits small cell clusters (arrowheads) which are not sufficient to drive tumour formation, leading to hyperplasia only (also in Appendix Fig. S1). (**B**) The number of clusters (**B**) and the volume of β-catenin cell clusters (**C**) are significantly higher in the *Cre;Apc*$^{LoxP15/15}$ and the *Cre;Apc*$^{LoxP1-15/15}$ compared to the *Cre;Apc*$^{LoxP1-15/1-15}$ milder phenotype. (**D**) Western blot against p21 from progenitor/stem cells derived from the 3 different genotypes and controls (*Apc*$^{LoxP15/15}$), reveals that p21 is highly expressed in the *Cre;Apc*$^{LoxP15/15}$ and to a lesser extent in the *Cre;Apc*$^{LoxP1-15/15}$ compared to the *Cre;Apc*$^{LoxP1-15/1-15}$. (**E**) Quantification of the relative protein expression of p21 normalised to β-actin shows that levels of p21 increase with the severity of the phenotype, with cells derived from *Cre;Apc*$^{LoxP15/15}$ expressing the highest levels of p21, suggesting a stronger p21-mediated senescence function. p21 was found to be upregulated in the β-catenin cell clusters by double immunofluorescence (see Appendix Fig. S7). Images in (**A**) are representative of 4 embryos per genotype. *P* values in (**B**, **C**, **E**) were obtained using one-way ANOVA and data represented as mean ± SEM, from three pituitaries per genotype and in triplicate independent experiments. AL anterior lobe, MZ marginal zone, PL posterior lobe, SB sphenoid bone. Scale bars in (**A**) represent 50 μm.

 © The Author(s)

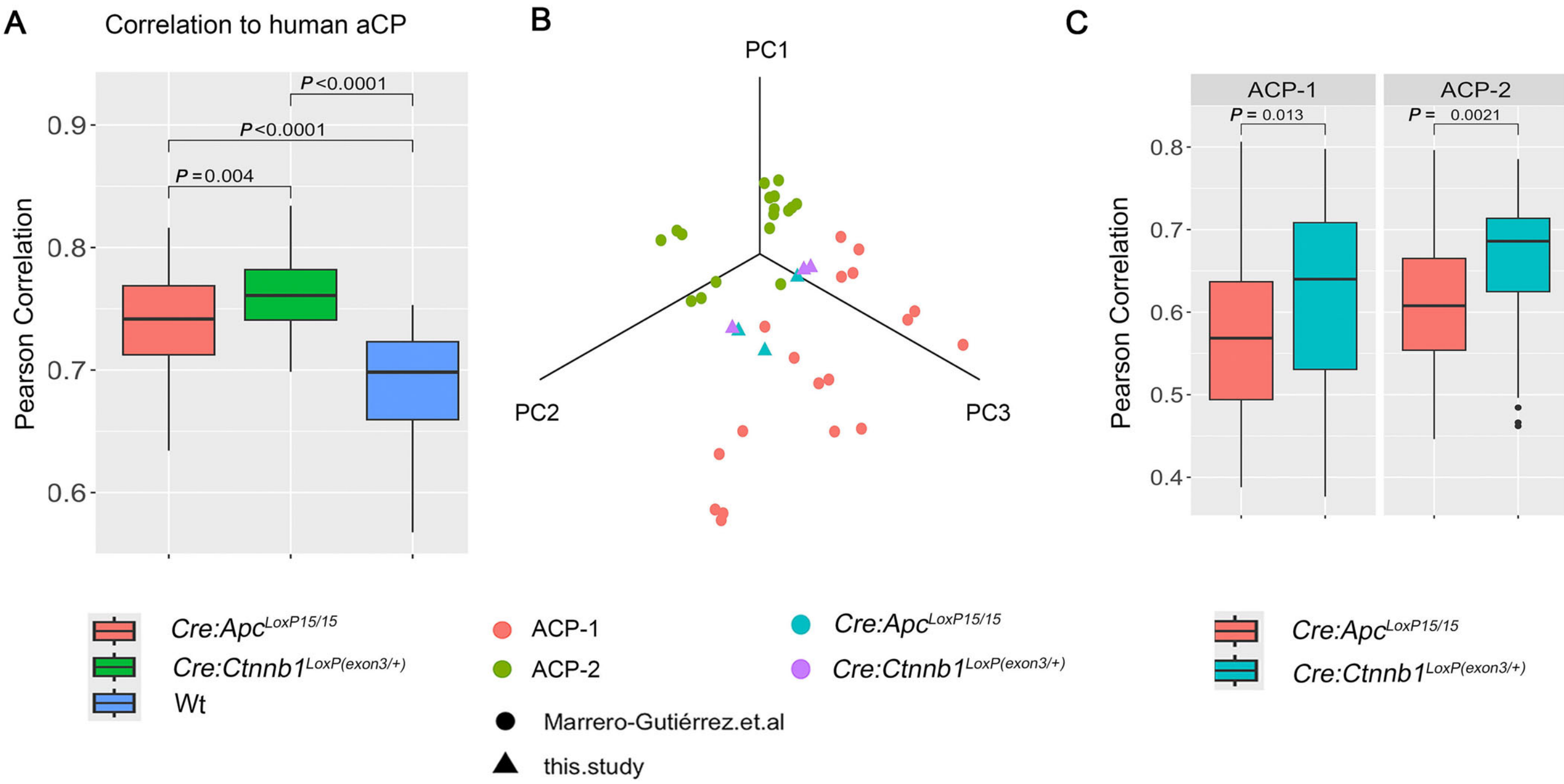

Figure EV3. Methylation analyses among aCP genetic subtypes.

(**A**) Boxplot showing Pearson Correlations between DNA samples from *Cre:Apc*$^{LoxP15/15}$ or *Cre:Ctnnb1*$^{LoxP(exon3)/+}$ tumours or Wt murine pituitaries compared to human aCPs samples from Capper et al, 2018 revealed correlation between murine *Acp* and *Ctnnb1.* Reported *P* values calculated by Wilcoxon test. (**B**) Principal component analysis of DNA methylation samples from *Cre:Apc*$^{LoxP15/15}$ or *Cre:Ctnnb1*$^{LoxP(exon3)/+}$ tumours and Wt murine pituitaries alongside reference human samples from Marrero-Gutierrez, et al, 2024 study. (**C**) Boxplot showing Pearson correlations between DNA samples from and *Cre:ApcLoxP*$^{15/15}$ or *Cre: Ctnnb1*$^{LoxP(Exon3)/+}$ tumours and human samples split according to two reported methylation subtypes ACP *Ctnnb1* mutation status (right). Reported *P* values calculated by the Wilcoxon test. For boxplots in (**A**) and (**C**), the centre line represents the median (50th percentile), box bounds represent the 25th and 75th percentiles, and the lower and upper whiskers represent the minimum and maximum observed values, respectively, within 1.5× the interquartile range; values beyond the whiskers are plotted individually as outliers.

© The Author(s)
