# Supplementary material for: Apc-deficient pituitary stem cells drive adamantinomatous craniopharyngioma through senescence-associated inflammation
Source: EMBO Mol Med. 2026 Oct 9;18(11):4386–412. doi: 10.1038/s44321-026-00525-2 (PMC13748607; doi:10.1038/s44321-026-00525-2)
Supplement: Supplementary file 2 [file 44321_2026_525_MOESM1_ESM.pdf]

Appendix Table of Content:

Appendix Figure S1	p2
Appendix Figure S2	p3
Appendix Figure S3	p4
Appendix Figure S4	p5
Appendix Figure S5	p6
Appendix Figure S6	p7
Appendix Figure S7	p8
Appendix Figure S8	p9

Appendix Figure S1

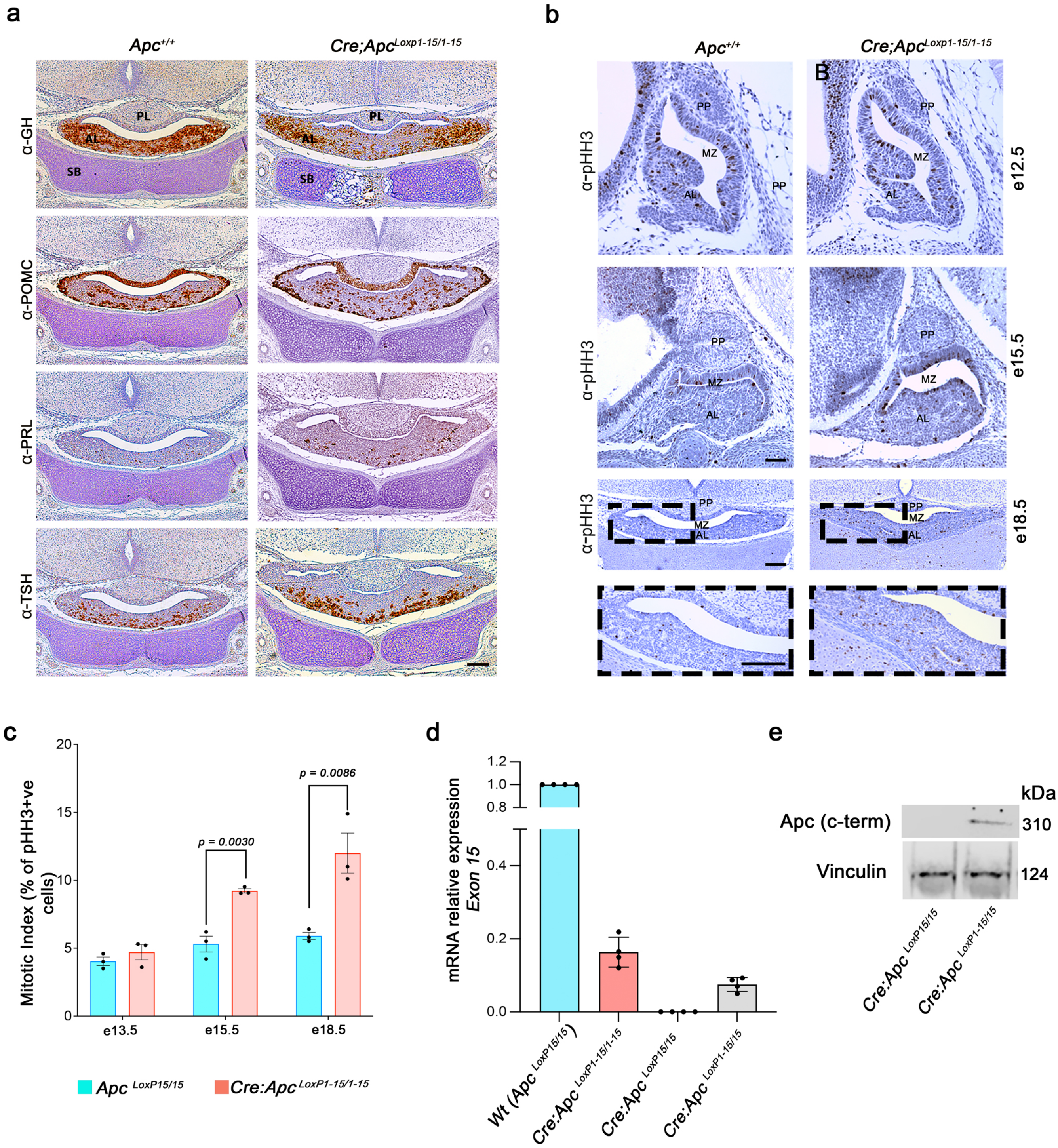

Appendix Figure S1. Deletion of *Apc* exon1 to exon15 in the pituitary progenitors, *Cre:Apc*$^{LoxP1\text{-}15/1\text{-}15}$, results in hyperplasia of the pituitary gland but not tumour formation. a) IHC of coronal section through the pituitary gland and brain at e18.5 against GH, POMC, PRL and TSH of Wt (left column) or *Cre:Apc*$^{LoxP1\text{-}15/1\text{-}15}$ mutant (right column) reveals that the mutant pituitaries exhibit hyperplasia with decreased in GH hormone producing cells. **b**) IHC against the proliferation marker pHH3 at e12.5, e15.5 and e18,5 reveals increased number of pHH3+ve cells in *Cre:Apc*$^{LoxP1\text{-}15/1\text{-}15}$ mutant compared to Wt pituitaries leading to statistically significant increased mitotic index **c**. **d**) RT-qPCR to amplify exon15 of Apc using controls (*Apc*$^{LoxP15/15}$) or FAC-sorted cells from *Cre:Apc*$^{LoxP1\text{-}15/1\text{-}15}$, *Cre:Apc*$^{LoxP15/15}$ or the compound heterozygote *Cre:Apc*$^{LoxP1\text{-}15/15}$. Exon15 was fully recombined in the *Cre:Apc*$^{LoxP15/15}$, but exon 15 of *Apc* was being detected in the *Cre:Apc*$^{LoxP1\text{-}15/1\text{-}15}$ at 16.5% and 7.5% in the *Cre:Apc*$^{LoxP1\text{-}15/15}$ indicating incomplete recombination of the *Apc LoxP exon 1-15* locus. **e**) Western blot identified Wt-Apc protein using an antibody against the C-terminal part of Apc in cell cultures from progenitors derived from *Cre:Apc*$^{LoxP1\text{-}15/15}$ whilst no Wt-Apc protein was detected in cells derived from *Cre:Apc*$^{LoxP15/15}$ validating the RT-qPCR results. *P* values in **c** were obtained using unpaired two-tailed Student's T-test and data represented as mean ± SEM, from 3 pituitaries per genotype. Images are representative of 4-5 embryos per genotype. Western blots are representative of 4 independent experiments. Abbreviations: AL, anterior lobe; LH, luteinizing hormone; GH, growth hormone; H&E, haematoxylin and eosin; IHC, immuno-histochemistry; MZ, marginal zone; PL, posterior lobe; POMC, Pro-opiomelanocortin; PRL, prolactin; SB, sphenoid bone; TSH thyroid-stimulating hormone. Scale bars in **a** and **b** represent 100 μm.

Appendix Figure S2

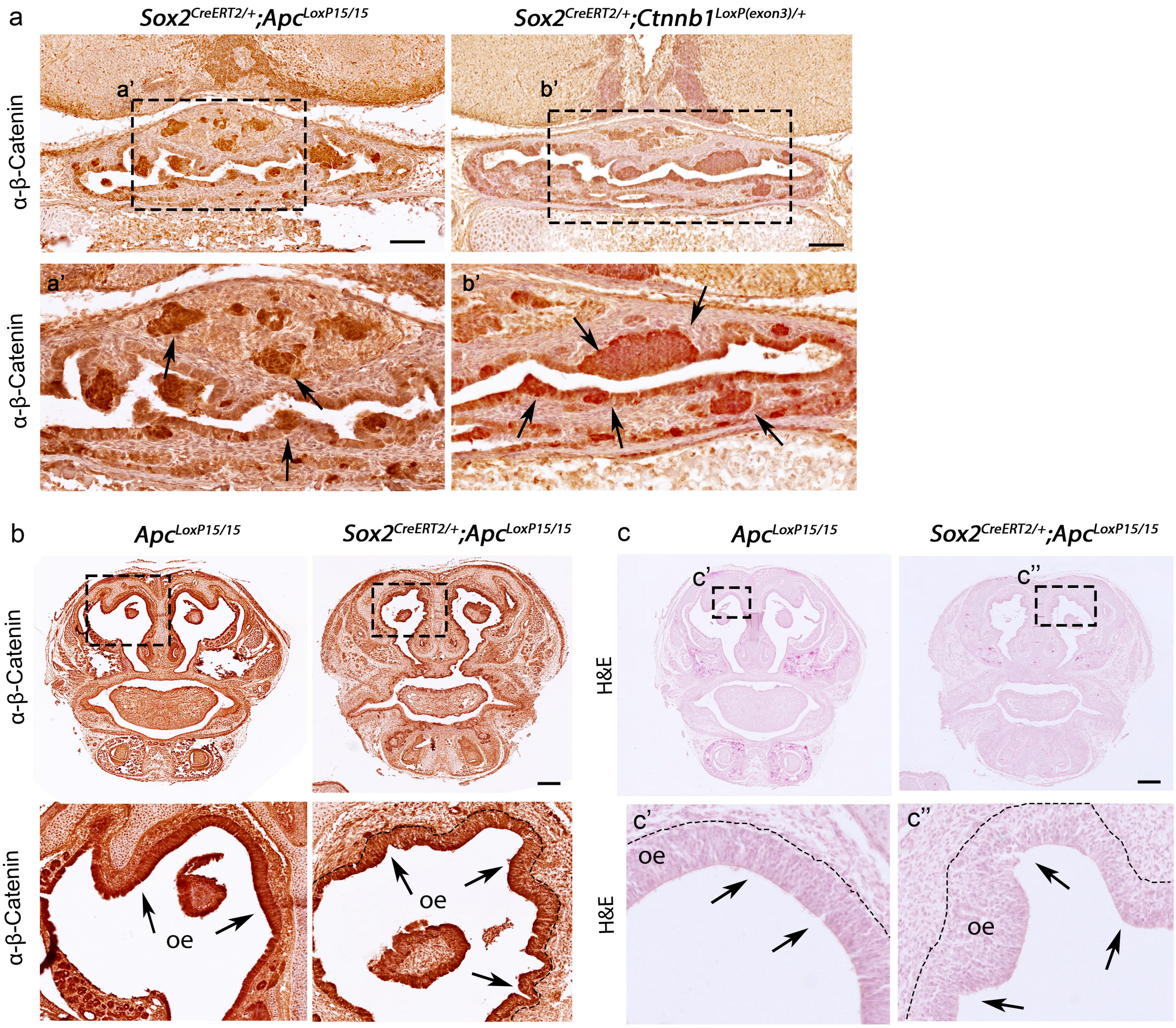

Appendix Figure S2. Activation of oncogenic β-catenin (*$Sox2^{CreERT2/+}$,$Ctnnb1^{LoxP(exon3/+}$*) and genetic deletion of *Apc exon15* (*$Sox2^{CreERT2/+}$,$Apc^{LoxP15/15}$*) in the Sox2+ve stem cells leads to aCP tumour formation during embryogenesis and to olfactory epithelium hyperplasia. a) IHC against β-catenin on coronal sections through *$Sox2^{CreERT2/+}$;$Apc^{LoxP15/15}$* or *$Sox2^{CreERT2/+}$;$Ctnnb1^{LoxP(exon3/+)}$* Tam-induced embryos shows tumours of the pituitary gland exhibiting large clusters of whorl-like cells that over-express nuclear β-catenin (arrows) which is diagnostic hallmark of aCPs. **b**) Coronal sections through olfactory region of controls *$Apc^{Loxp15/15}$* or tamoxifen induced mutant *$Sox2^{CreERT2/+}$,$Apc^{LoxP15/15}$* IHC for β-catenin (b) or H&E stained (c) show abnormal hyperplastic olfactory epithelium (oe) composed of abnormal extra folding and thickening of the epithelial layer. c) H&E staining show abnormal cell disorganisation of the olfactory epithelium in Tam-induced *$Sox2^{CreERT2/+}$,$Apc^{LoxP15/15}$* embryos (c'' arrows) compared to control littermates (c' arrows). a', b', c' and c'' represent enlarged images of the square-dashed areas respectively. Images are representative of 4 embryos per genotype. Abbreviations: H&E, haematoxylin and eosin; IHC, immuno-histo chemistry; oe, olfactory epithelium. Scale bars in **a** represent 200 μm and in **b** & **c** represent 50 μm.

Appnedix Figure S3

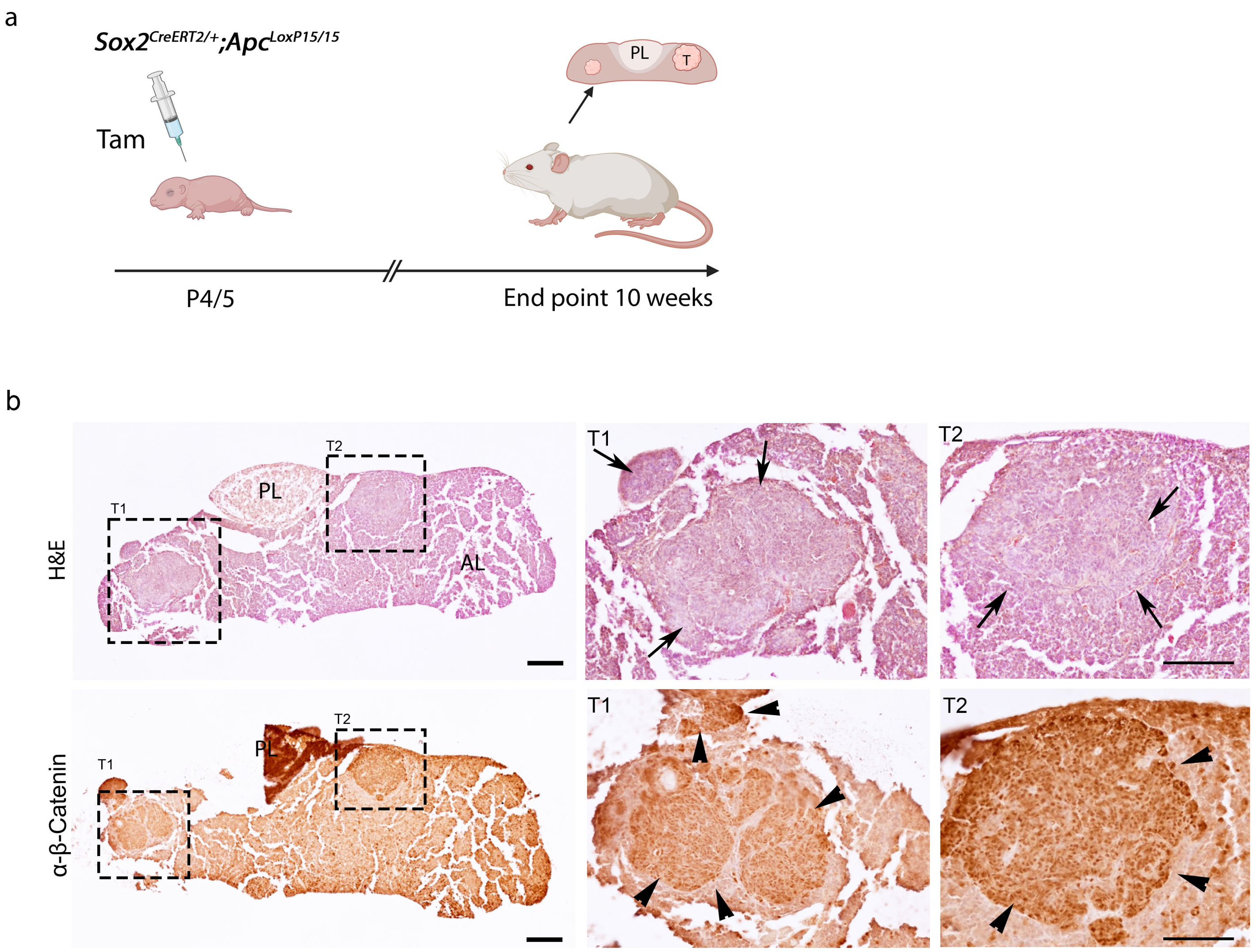

Appendix Figure S3. Postnatal deletion of *Apc* exon15 in the Sox2+ve stem cells results in tumour formation. a) Experimental diagram: to achieve deletion of *Apc* exon15 postnatally *$Sox2^{CreERT2/+}$;$Apc^{LoxP15/15}$* pups were Tam injected (0.1 mg/g of body weight) with 2 injections at postnatal day 4 and 5. Pituitaries were dissected at 10 weeks of age post Tam-induction and histological analyses performed to ascertain presence of tumours. **b**) H&E sections through the pituitary gland of 10 weeks old mice post Tam-injection revealed the presence of tumours growing within the pituitary AL (T1 and T2). T1 and T2 are magnified images of the squared dashed areas, showing histological abnormalities with presence of whorl-like cell clusters within the AL. IHC against β-catenin on consecutive sections revealed that these whorl-like cell clusters are positive for nuclear β-catenin. Images are representative of 4 postnatal induced *$Sox2^{CreERT2/+}$;$Apc^{LoxP15/15}$* adult mice. Abbreviations: AL, anterior lobe; H&E: haematoxylin and eosin; P, postnatal day; PL, posterior lobe; T, tumour. Scale bars in **b** represent 100 μm.

Appendix Figure S4

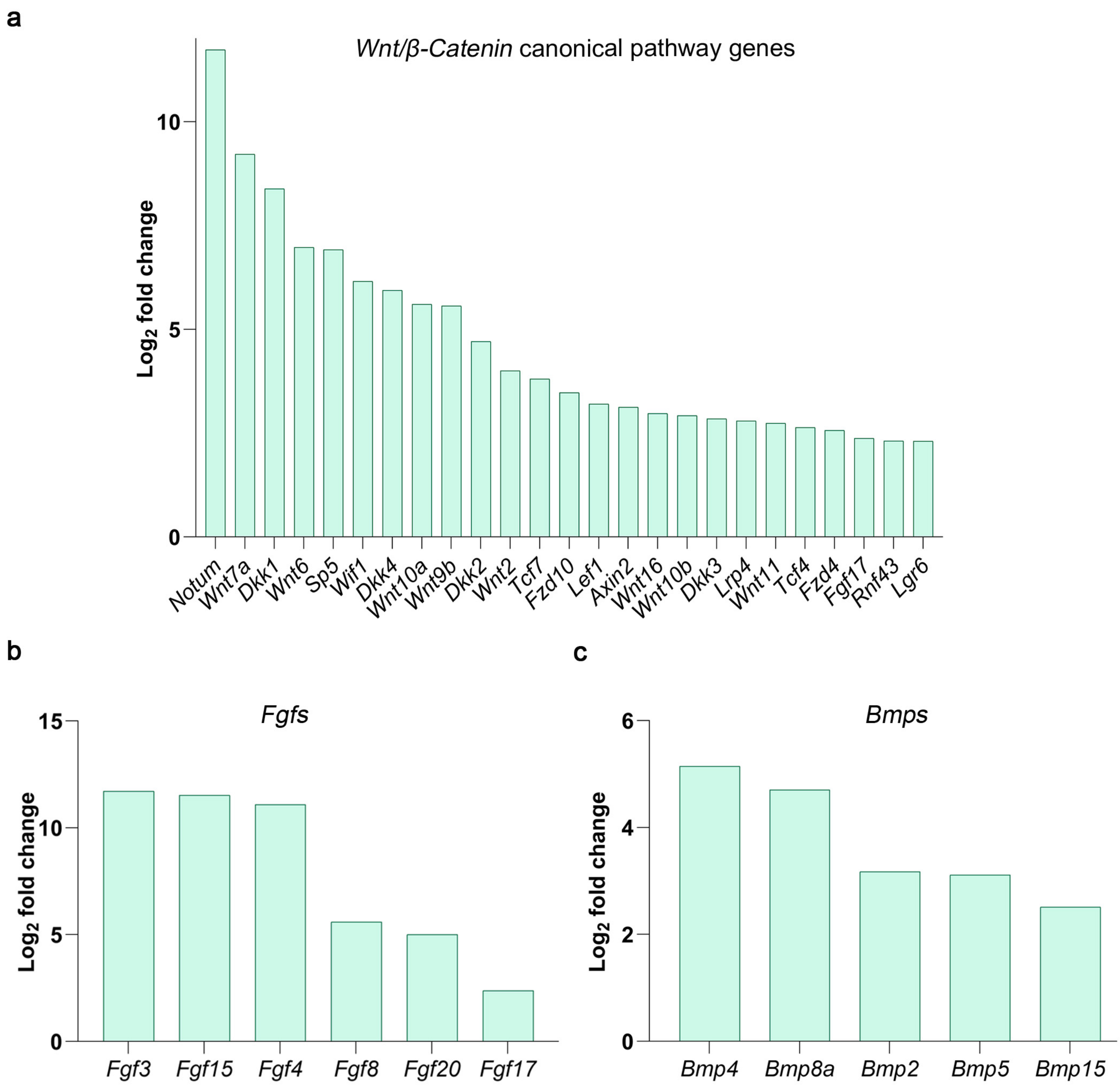

Appendix Figure S4. The β-catenin+ve GFP+ve cell clusters from *Cre:Apc*$^{LoxP15/15}$*;Lef-1:GFP* tumours over activate the Wnt/β-catenin canonical pathway, Fibroblast growth factors (Fgfs) and Bone morphogenetic (Bmps) pathways. **a**) Graph representing Log_2 fold change of upregulated genes belonging to the Wnt/β-catenin canonical pathway. The Wnt-downstream targets *Axin2*, *Lef1*, *Sp5*, *Tcf4*; the secreted ligands: *Wnt7a*, *Wnt6*, *Wnt10a*, *Wnt9b*, *Wnt2*, *Wnt16, Wnt11* and their receptors and co-receptors *Fzd4*, *Fzd10*, *Lrp4, Lgr6*. Moreover, increase in secreted Wnt antagonist *Notum, Dkk1, Wif1*, *Dkk4*, *Dkk2*, *Dkk3* were found to be highly upregulated in the *Apc*-driven β-catenin+ve GFP+ve cell clusters. **b**) Genes belonging to the secreted fibroblast growth factor (*Fgfs*): *Fgf3*, *Fgf15*, *Fgf4*, *Fgf8, Fgf20* and *Fgf17* and the bone morphogenic factors (**c**): *Bmp4, Bmp8a*, *Bmp2*, *Bmp5*, *Bmp15* were found upregulated by the *Apc*-driven β-catenin+ve GFP+ve cell clusters.

Appendix Figure S5

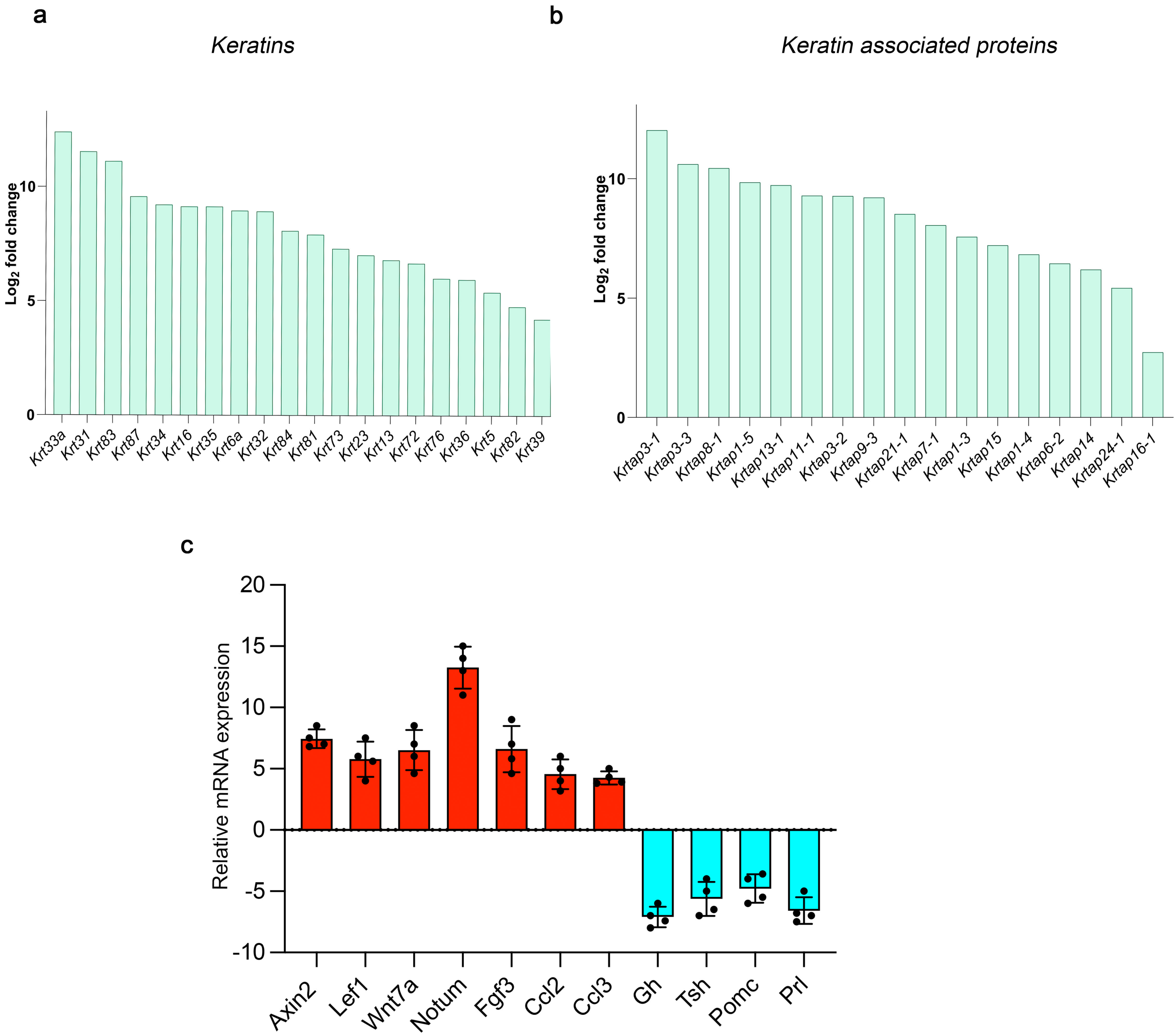

Appendix Figure S5. ***Apc*-driven clusters of accumulating β-catenin (β-catenin+ve GFP+ve cell clusters) from *Cre:Apc$^{loxP15/15}$;Lef-1:GFP* tumours over-express numerous cytokeratin and cytokeratin's associated proteins**. **a**) Graph representing Log_2 fold change of the most upregulated cytokeratins identified from the transcriptomic analyses. **b**) Graph representing Log_2 fold change of the most upregulated cytokeratin's associated protein identified in the transcriptomic analyses. **c**) Validation of the Apc-driven β-catenin+ve GFP+ve cells clusters transcriptomic analyses by RT-qPCR of some of the upregulated genes (red) *Axin2* (p<0.0001), *Lef1* (*p*=0.0025), *Wnt7a* (*p*=0.0001), *Notum* (*p*=0.0024), *Fgf3* (*p*=0.0039); *Ccl2* (*p*=0.0043), *Ccl3* (*p*=0.0003) and downregulated genes (blue) *Gh* (*p*=0.0001), *Tsh* (*p*=0.0005), *Pomc* (*p*=0.0004), *Prl* (*p*=0.0001). *P* values in **c** were obtained using unpaired two-tailed Student's T-test and data represented as mean ± SEM, from 3-4 pituitaries per genotype.

Appendix Figure S6

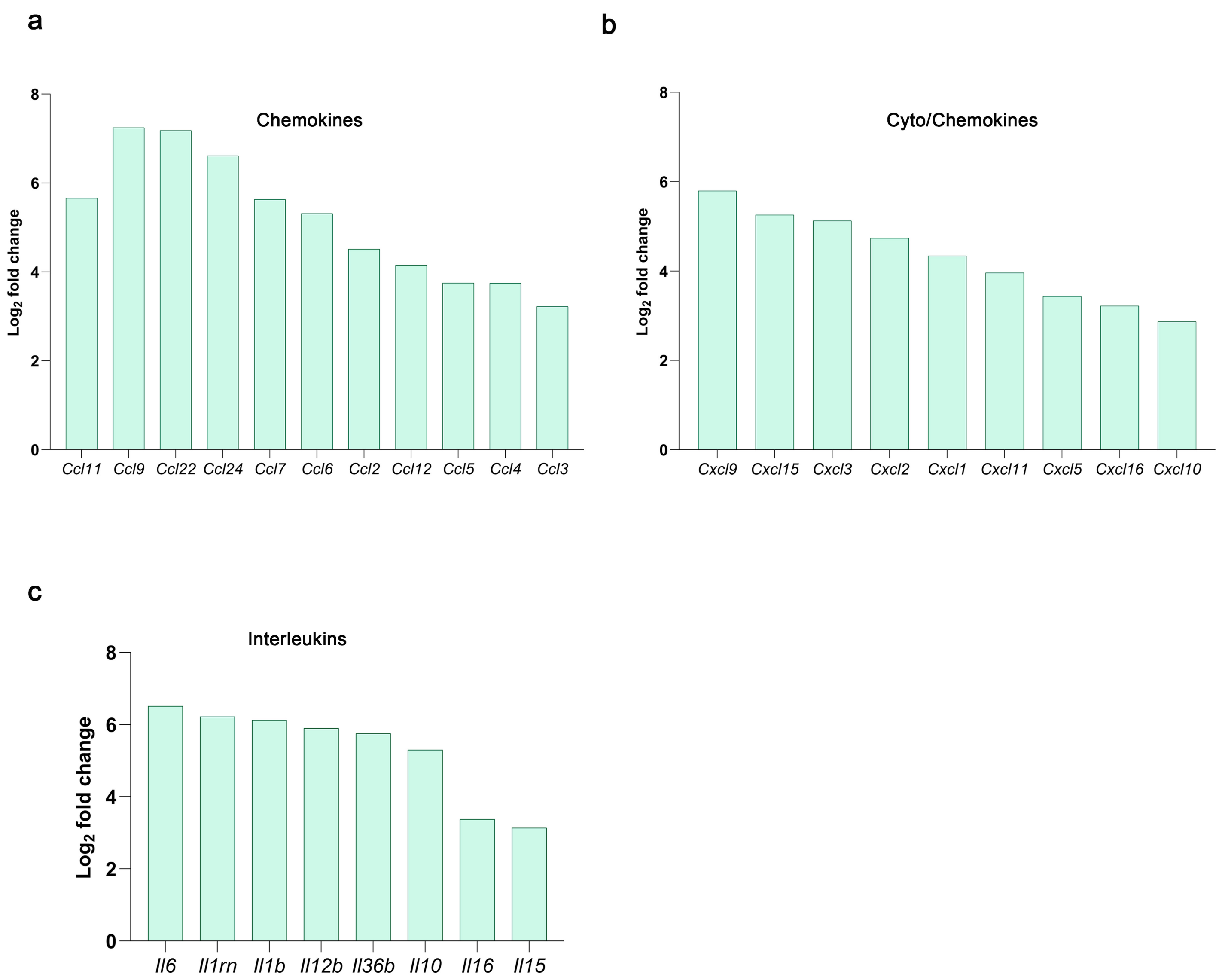

Appendix Figure S6. *Apc*-driven clusters of accumulating β-catenin (β-catenin+ve GFP+ve) from the *Cre:Apc$^{LoxP15/15}$;Lef-1:GFP* tumours overexpress high levels of multiple cytokines, chemokines and interleukins. a) Graph representing Log_2 fold change of the most upregulated chemokines. **b**) Graph representing Log_2 fold change of the most upregulated cytokines expressed by the β-catenin+ve GFP+ve cell clusters. **c**) Graph representing Log_2 fold change of the most upregulated interleukins expressed by the β-catenin+ve GFP+ve cell clusters.

Appendix Figure S7

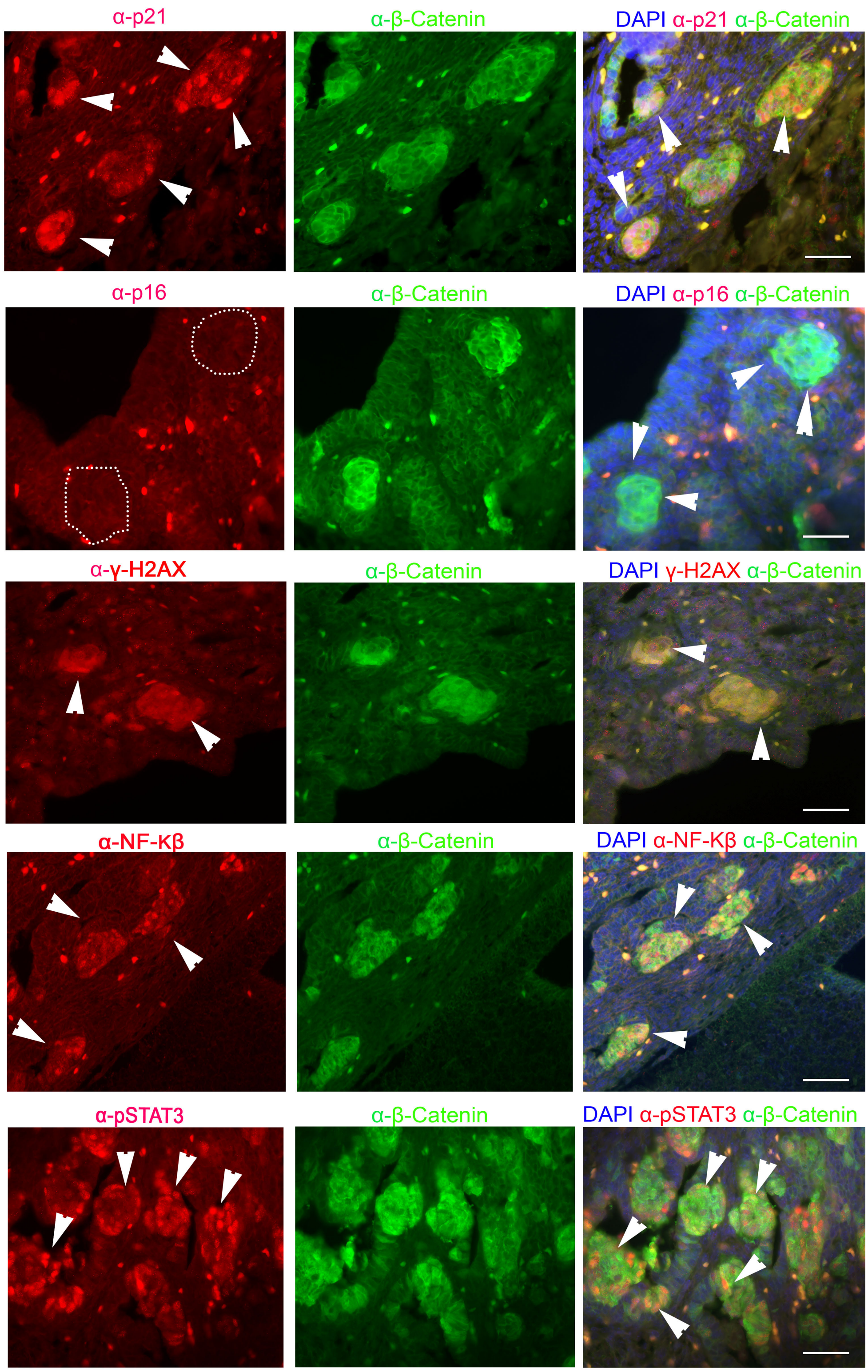

Appendix Figure S7. *Apc*-driven clusters of accumulating β-catenin express the senescence associated markers: p21, γ-H2AX, NF-Kβ (p65) and pSTAT3. Double immunofluorescence against β-catenin (green) and p21(red), γ-H2AX (red), NF-Kβ (p65, red) and pSTAT3 (red) showed that these proteins (arrowheads) are highly upregulated withing the nucleo-cytoplasmic β-catenin+ve cell clusters (white arrowheads in the merged image). The senescence marker p16 was not found expressed within the clusters of accumulating β-catenin (white dotted circles). Interestingly, not all the cells within the clusters equally express the γ-H2AX, NF-Kβ (p65) and pSTAT3 suggesting a cell heterogenicity within the β-catenin+ve cell clusters. pSTAT3 a downstream mediator of the SASP-inflammatory cytokine IL6 was found upregulated withing some clusters and adjacent cells. These results validate our mRNA-seq data. Images are representative of 3 *Cre:Apc*$^{LoxP15/15}$ tumours. Scale bars represent 50 μm.

Appendix Figure S8

a

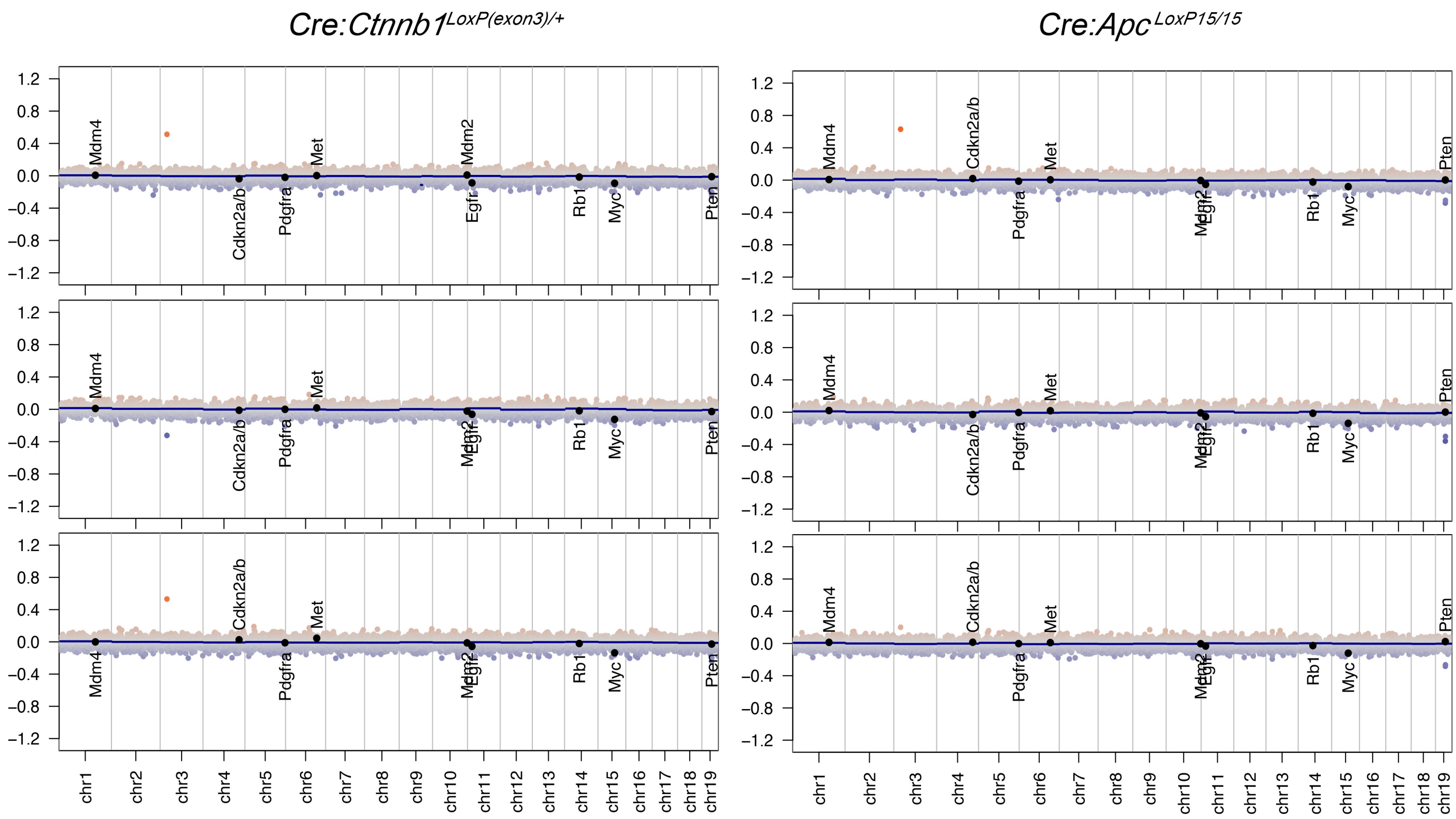

Appendix Figure S8. Genome plots, showing an absence of detectable copy number variations in the *Cre:Apc*$^{LoxP15/15}$ and *Cre:Ctnnb1*$^{LoxP(exon3)/+}$ tumours. Genes commonly amplified or deleted in cancer are labelled.
