# Supplementary material for: Apc-deficient pituitary stem cells drive adamantinomatous craniopharyngioma through senescence-associated inflammation
Source: EMBO Mol Med. 2026 Oct 9;18(11):4386–412. doi: 10.1038/s44321-026-00525-2 (PMC13748607; doi:10.1038/s44321-026-00525-2)
Supplement: Supplementary file 3 [file 44321_2026_525_MOESM2_ESM.pdf]

EMBO Molecular Medicine

Apc-deficient pituitary stem cells drive adamantinomatous craniopharyngioma through senescence-associated inflammation

James Blackburn, James Nicholson, Laura Gomez Corral, Jingyi Xue, Ashutosh Rai, Byan Finn, Rachael Tan, Kalyana Gangaraju, Angelica Gualtieri, Charlotte Hall, Gabor Czibik, Pinaki Dutta, Márta Korbonits, Federico Roncaroli, Shannon Davis, and Carles Gaston-Massuet

Corresponding author: Carles Gaston-Massuet (c.gaston-massuet@qmul.ac.uk)

Review Timeline:

Submission Date:	15th Dec 25
Editorial Decision:	2nd Feb 26
Revision Received:	8th Jul 26
Editorial Decision:	19th Aug 26
Revision Received:	30th Aug 26
Accepted:	8th Sep 26

Editor: Zeljko Durdevic

Transaction Report:

(Note: With the exception of the correction of typographical or spelling errors that could be a source of ambiguity, letters and reports are not edited. Depending on transfer agreements, referee reports obtained elsewhere may or may not be included in this compilation. Referee reports are anonymous unless the Referee chooses to sign their reports.)

2nd Feb 2026

Dear Dr. Gaston-Massuet,

Thank you for the submission of your manuscript to EMBO Molecular Medicine. We have now received feedback from the three reviewers who agreed to evaluate your manuscript. While the referee #2 supports publication of the manuscript, referees #1 and #3 recognize potential interest of the study but also raise important concerns that should be addressed in a major revision. If you would like to discuss further the points raised by the referees, I am available to do so via email or video. Let me know if you are interested in this option.

Further consideration of a revision that addresses reviewers' concerns in full will entail a second round of review. EMBO Molecular Medicine encourages a single round of revision only and therefore, acceptance or rejection of the manuscript will depend on the completeness of your responses included in the next, final version of the manuscript. For this reason, and to save you from any frustrations in the end, I would strongly advise against returning an incomplete revision.

We would welcome the submission of a revised version within three months for further consideration. Please let us know if you require longer to complete the revision.

I look forward to receiving your revised manuscript.

Yours sincerely,

Zeljko Durdevic

Zeljko Durdevic
Senior Editor
EMBO Molecular Medicine

*****
When preparing your revised manuscript, please refer to our guidelines: https://link.springer.com/journal/44321/submission-guidelines#cms-Revised-submissions. We perform an initial quality control of all revised manuscripts before re-review; failure to include requested items will delay the evaluation of your revision.

We require:

1) A .docx formatted version of the manuscript text (including legends for main figures, EV figures and tables). Please make sure that the changes are highlighted to be clearly visible.

2) Individual production quality figure files as .eps, .tif, .jpg (one file per figure). For guidance, download the 'Figure Guide PDF': https://media.springernature.com/original/springer-cms/rest/v1/content/27825798/data/v1.

3) A .docx formatted letter INCLUDING the reviewers' reports and your detailed point-by-point responses to their comments. As part of the EMBO Press transparent editorial process, the point-by-point response is part of the Review Process File (RPF), which will be published alongside your paper.

4) A complete author checklist, which you can download from our author guidelines. Please insert information in the checklist that is also reflected in the manuscript. The completed author checklist will also be part of the RPF.

5) Please note that all corresponding authors are required to supply an ORCID ID for their name upon submission of a revised manuscript.

6) It is mandatory to include a 'Data Availability' section after the Materials and Methods. Before submitting your revision, primary datasets produced in this study need to be deposited in an appropriate public database, and the accession numbers and

database listed under 'Data Availability'. Please remember to provide a reviewer password if the datasets are not yet public.

In case you have no data that requires deposition in a public database, please state so in this section. Note that the Data Availability Section is restricted to new primary data that are part of this study.

7) For data quantification: please specify the name of the statistical test used to generate error bars and P values, the number (n) of independent experiments (specify technical or biological replicates) underlying each data point and the test used to calculate p-values in each figure legend. The figure legends should contain a basic description of n, P and the test applied. Graphs must include a description of the bars and the error bars (s.d., s.e.m.).

8) At EMBO Press we ask authors to provide source data for the main manuscript figures. You will receive a separate email with instructions for providing source data with your revised manuscript, including how to upload and organize the files.

9) Our journal encourages inclusion of *data citations in the reference list* to directly cite datasets that were re-used and obtained from public databases. Data citations in the article text are distinct from normal bibliographical citations and should directly link to the database records from which the data can be accessed. In the main text, data citations are formatted as follows: "Data ref: Smith et al, 2001" or "Data ref: NCBI Sequence Read Archive PRJNA342805, 2017". In the Reference list, data citations must be labeled with "[DATASET]". A data reference must provide the database name, accession number/identifiers and a resolvable link to the landing page from which the data can be accessed at the end of the reference.

10) We replaced Supplementary Information with Expanded View (EV) Figures and Tables that are collapsible/expandable online. A maximum of 5 EV Figures can be typeset. EV Figures should be cited as 'Figure EV1, Figure EV2" etc... in the text and their respective legends should be included in the main text after the legends of regular figures.

- For the figures that you do NOT wish to display as Expanded View figures, they should be bundled together with their legends in a single PDF file called *Appendix*, which should start with a short Table of Content. Appendix figures should be referred to in the main text as: "Appendix Figure S1, Appendix Figure S2" etc.

- Additional Tables/Datasets should be labeled and referred to as Table EV1, Dataset EV1, etc. Legends have to be provided in a separate tab in case of .xls files. Alternatively, the legend can be supplied as a separate text file (README) and zipped together with the Table/Dataset file.

11) The paper explained: EMBO Molecular Medicine articles are accompanied by a summary of the articles to emphasize the major findings in the paper and their medical implications for the non-specialist reader. Please provide a draft summary of your article highlighting

- the medical issue you are addressing,

- the results obtained and

- their clinical impact.

This may be edited to ensure that readers understand the significance and context of the research. Please refer to any of our published articles for an example.

12) Author contributions: You will be asked to provide CRediT (Contributor Role Taxonomy) terms in the submission system. These replace a narrative author contribution section in the manuscript.

13) A Conflict of Interest statement should be provided in the main text.

14) Every published paper includes a 'Synopsis' to further enhance discoverability. Synopses are displayed on the journal webpage and are freely accessible to all readers. They include a short stand first (maximum of 300 characters, including space) as well as 2-5 one-sentences bullet points that summarizes the paper. Please write the bullet points to summarize the key NEW findings. They should be designed to be complementary to the abstract - i.e. not repeat the same text. We encourage inclusion of key acronyms and quantitative information (maximum of 30 words / bullet point). Please use the passive voice. Please attach these in a separate file or send them by email, we will incorporate them accordingly.

Please also suggest a striking image or visual abstract to illustrate your article as a PNG file 550 px wide x 300-600 px high.

15) Include a Reagents and Tools Table as part of the Methods section, which can be downloaded from our author guidelines.

EMBO Molecular Medicine has a "scooping protection" policy, whereby similar findings that are published by others during review or revision are not a criterion for rejection. Should you decide to submit a revised version, I do ask that you get in touch

after three months if you have not completed it, to update us on the status.

***** Reviewer's comments *****

Referee #1 (Remarks for Author):

This is a well-done manuscript on the role of Apc in Prop1 and Sox2-positive pituitary progenitor cells. The work shows that constitutive activation of WNT signaling by deleting Apc is sufficient to drive murine tumors that resemble human craniopharyngeomas. Here are may comments and concerns:
- It is, in my view, a little misleading to state that "a proportion of aCPs are driven by mutations in the CTNNB1 gene encoding for β -catenin", while the current knowledge states CTNNB1 mutations are present in virtually all aCPs (51/53 tumor in Brastianos et al). Similarly, please dont state that „main genetic drivers for a proportion of aCPs remain unknown" and be more honest in this regard. This will not diminish the impact on your work, but will improve your manuscript!
- Sox2-creERT2 mice have also been used to drive an exon3-deleted variant of Ctnnb1, which apparently resulted in lesions of the olfactory epithelium (but not in the pituitary?, see PMID: 27902722). How does the pituitary look like in Sox2-creERT2:Ctnnb1 (exon3 deleted) mice and the olfactory epithelium in Sox2-creERT2:Apc mice?
- Please provide larger high-quality and high power H&E images of the murine aCPs shown in Fig. 1, ideally side by side with human aCPs and murine aCPs from Ctnnb1-driven tumors. By the way: Are there morphological, transcriptional or epigentic differences of human aCPs depending on whether they are driven by APC or CTNNB1?
- Classification and granularity of human tumors is currently best reflected by global DNA methylation, and mouse tumor epigenetics may actually well reflect the humand counterparts (see PMID: 41381884). How well do the murine aCPs match human aCPs regarding global DNA methylation. How do copy number profiles of these tumors look like?

Referee #2 (Remarks for Author):
Suitable for publication.

Referee #3 (Remarks for Author):

This manuscript investigates whether loss of the Wnt pathway antagonist APC in pituitary progenitor/stem cells can drive the development of aCP.
The authors conclude that APC dysfunction is a causal driver of aCP and propose that APC-mutant aCP represents a novel genetic subtype with clinical implications for FAP/APC syndrome patients.
The study is ambitious, mechanistically rich, and addresses an important gap in aCP genetics. The mouse models are compelling and the transcriptomic work adds depth. However, several key issues regarding interpretation, controls, and translational claims need clarification before publication.

The authors should temper the language and clarify that: APC loss represents an alternative genetic mechanism leading to Wnt/β-catenin activation, rather than a β-catenin-independent pathway.
The conclusion that 7.5% residual Apc expression defines a hypomorphic tumour-permissive state is very interesting but currently underdeveloped.

The Sox2CreERT2 experiment shows early cluster formation, but the claim that $Sox2^{+}$ cells are the cell of origin is stronger than the data.

The distinction that Apc-driven clusters use p21 but not p16 is intriguing and potentially important. Evidence is limited to transcript levels. Should temper to:
"These clusters display a transcriptional profile consistent with a SASP-like program".

The manuscript should more directly discuss prior work by Gonzalez-Meljem et al. (Nat Commun 2017), which included Apc knockout models in the context of pituitary tumourigenesis. In Gonzalez-Meljem's, loss of Apc did result in β-catenin accumulation and formation of small clusters with some senescence markers, but did not lead to frank tumour development and was associated with an attenuated SASP relative to oncogenic Ctnnb1 models. This highlights an important conceptual distinction: while Apc loss activates Wnt/β-catenin signalling, the strength and paracrine impact of the resulting clusters differ from those driven by stabilized β-catenin in prior models. The authors should clarify how their models and tumour outcomes compare to these published Apc loss experiments and explain what novel mechanistic or biological insights are contributed beyond these earlier observations."

Reply to the Reviewers comments for the manuscript: James Blackburn et al**. "***Disruption of the Wnt-antagonist APC in pituitary stem cells drives the development of adamantinomatous craniopharyngioma"*

We would like to sincerely thank the Reviewers for their thoughtful, constructive, and insightful evaluation of our manuscript. We greatly appreciate their positive assessment of our study as: *"well done, ambitious, mechanistically rich, and addressing an important gap in aCP genetics."*

We have carefully considered all comments and suggestions and have substantially strengthened the manuscript in response. In addition to revisions of the text, we have performed several new experiments, including additional histopathological analyses, postnatal tamoxifen induction studies, protein-level validation of SASP to strengthen our mRNA-seq data and cross-species methylome analyses comparing our murine models with human adamantinomatous craniopharyngioma (aCP).

We are grateful for the Reviewers' suggestions, which have significantly improved the quality and clarity of the manuscript. All changes made in response to the Reviewers' comments are highlighted in grey in the main revised manuscript version for ease of reference.

Reviewer 1

Comment 1: *"It is, in my view, a little misleading to state that 'a proportion of aCPs are driven by mutations in the CTNNB1 gene encoding* β*-catenin', while current knowledge suggests that CTNNB1 mutations are present in virtually all aCPs."*

Response: We thank the Reviewer for this observation. We have revised the text throughout the manuscript The revised text now reads:

"Although most of aCPs are driven by somatic mutations in β-catenin (*CTNNB1*) some cases remain genetically unresolved." This modification has been implemented throughout the manuscript.

Comment 2: *"Similarly, please don't state that 'main genetic drivers for a proportion of aCPs remain unknown' and be more honest in this regard."*

Response: We thank the Reviewer for this valuable suggestion. Accordingly, we have revised the text throughout the manuscript to more accurately reflect the current state of knowledge and to avoid implying that unidentified genetic drivers represent a major proportion of cases.

Comment 3: *"Please provide larger high-quality and high-power H&E images of the murine aCPs shown in Fig. 1, ideally side by side with human aCPs and murine aCPs from Ctnnb1-driven tumours."*

Response: We thank the Reviewer for this excellent suggestion. To address this point, we have generated a new expanded view figure **(Fig. EV. 1) .** containing high-resolution histological images of *Apc*-driven murine tumours shown in Fig.1 (as requested), including both H&E and β-catenin immunostaining. For direct comparison, corresponding images from murine *Ctnnb1*-driven tumours and human *CTNNB1*-mutant aCP specimens are presented alongside the *Apc*-driven lesions.

These additions provide a more comprehensive histopathological comparison between the murine models and human disease and further support the conclusion that *Apc*-driven tumours faithfully recapitulate the characteristic pathological features of aCP.

Comment 4: *"Sox2-CreERT2 mice have also been used to drive an exon 3-deleted variant of Ctnnb1, resulting in lesions of the olfactory epithelium. How does the pituitary look in Sox2-CreERT2 mice and the olfactory epithelium in Sox2-CreERT2mice?"*

Response: We thank the Reviewer for this insightful question. As noted by the Reviewer, *Sox2-CreERT2* has previously been used to activate oncogenic β-catenin in Sox2-positive cells of the olfactory epithelium (Neils et al., 2016, PLOS1[1]). However, the pituitary gland was not examined in that study, and therefore direct assessment of pituitary pathology was not reported.

Importantly, *Sox2-CreERT*2-mediated activation of mutant β-catenin in pituitary Sox2-positive stem cells was previously performed (Andoniadou et al., 2013, Cell Stem Cells)[2], where we demonstrated that oncogenic β-catenin in Sox2-positive pituitary stem cells is sufficient to initiate aCP formation.

To directly address the Reviewer's question, we have now performed additional analyses and generated a new **Appendix Figure S2**.

First, we compared pituitary phenotypes in $Sox2^{CreERT2/+};Ctnnb1^{LoxP(exon3)/+}$ and $Sox2^{CreERT2/+};Apc^{LoxP15/15}$ mice. These analyses demonstrate that both models develop characteristic β-catenin-accumulating cell clusters and early tumour lesions, with remarkably similar histopathological features. In both cases, clusters of cells exhibiting nuclear accumulation of β-catenin are already evident by embryonic day 18.5.

Second, we examined the olfactory epithelium of $Sox2^{CreERT2/+};Apc^{LoxP15/15}$ mice. We observed epithelial hyperplasia and disruption of normal tissue architecture, resembling the phenotype reported following activation of mutant β-catenin in the olfactory epithelium. These findings further support the known roles of Wnt/β-catenin canonical signalling in maintaining olfactory epithelial homeostasis[3-5].

Comment 5: *"Are there morphological, transcriptional or epigenetic differences between human aCPs driven by APC or CTNNB1?"*

Response: We thank the Reviewer for this highly interesting question. To our knowledge, comprehensive studies directly comparing *APC*-mutant+ve and *CTNNB1*-mutant+ve human aCPs at the transcriptomic or epigenomic level have not yet been reported. We agree that such analyses would be highly informative and represent an important future direction for the field.

While human comparative datasets are currently unavailable, our study provides both transcriptomic and newly added methylomic analyses comparing *Apc*-driven and *Ctnnb1*-driven murine tumours and relating these findings to human aCP.

These analyses reveal substantial overlap between the two models, consistent with their shared activation of Wnt/β-catenin canonical pathway signalling (**Fig.8 b-c**). At the same time, we identify potentially important biological differences. Notably, *Apc*-driven tumours exhibit increased expression of p21-associated SASP components in the absence of detectable p16 activation (**Appendix Figure S7**). We also observe enhanced expression of keratins and keratin-associated proteins, which may contribute to the extensive wet-keratin deposits observed in *Apc*-driven tumours. These findings provide a useful framework for future studies investigating molecular heterogeneity among human aCP subtypes.

Comment 6: *"How well do the murine aCPs match human aCPs regarding global DNA methylation? How do copy number profiles of these tumours look?"*

Response: We thank the Reviewer for this excellent suggestion and agree that DNA methylation profiling provides one of the most robust approaches for tumour classification and cross-species comparison.

To directly address this point, we have performed new integrative methylomic analyses comparing *Apc*-driven and *Ctnnb1*-driven murine tumours compared with publicly available human aCP methylation dataset (suggested by the reviewer, Capper et al, 2018 [6]) using orthologous CpG probes shared between mouse and human methylation arrays.

These analyses revealed striking epigenetic concordance between murine and human tumours. Unsupervised principal component analysis demonstrated clear clustering of both murine tumour models with human aCP samples, whereas wild-type murine pituitary tissue clustered with non-neoplastic human control samples (new **Figure 8 d-f**).

This observation was further supported by quantitative correlation analyses, which demonstrated significantly stronger similarity between murine tumours and human aCP references than between tumours and control tissues (new **Fig. EV. 3**).

Interestingly, murine *Ctnnb1*-driven tumours showed the strongest similarity to the recently described human ACP-2 methylation subgroup[7] (enriched for *CTNNB1*-mutations) consistent with their shared underlying genetic driver (new **Fig. EV. 3 b-c**).

At the level of individual loci, differential methylation analyses identified a conserved set of hypomethylated genes shared between murine and human tumours, including *IL6*, *TLR4, PARP1* and *IGFBP4*. These genes are key regulators of inflammatory signalling, DNA damage responses and SASP-associated pathways. Their coordinated hypomethylation provides independent epigenetic support for a conserved activation of a SASP-like inflammatory programme in both species and across tumours subtypes (new **Figure 8 e-f**).

We additionally assessed copy number alterations (as requested by the reviewer) and did not identify recurrent CNVs in either murine model (new **Appendix Figure S8 a**). This finding is consistent with published human studies showing that primary aCPs are generally genomically stable and lack recurrent copy number alterations[8].

Together, these new analyses demonstrate that our murine models faithfully recapitulate key epigenetic features of human aCP while also reflecting the genomic stability characteristic of primary disease.

Reviewer 3:

Comment 1: *The authors should temper the language and clarify that: "-APC loss represents an alternative genetic mechanism leading to Wnt/*β*-catenin activation, rather than a* β*-catenin-independent pathway*.

Response: We thank the Reviewer for this clarification. We agree that our original wording was ambiguous. We have therefore revised the manuscript throughout to state that:

"APC disruption represents an alternative genetic mechanism leading to Wnt/β-catenin activation"

Comment 2: *The conclusion that 7.5% residual Apc expression defines a hypomorphic tumour-permissive state is very interesting but currently underdeveloped*.

Response: We agree that the biological significance of the 7.5% residual *Apc* expression required further clarification and strengthening. In our *Cre:Apc*$^{LoxP15/15}$ model, efficient recombination of exon 15 results in complete loss of exon 15 of APC and aggressive tumour development with postnatal lethality (**Fig. 1a**; **Appendix Figure S1d**). In contrast, the *Cre:Apc*$^{LoxP1-15/15}$ model shows significantly less aggressive tumour formation and retains detectable Apc wild-type mRNA (~7.5%).

To directly assess whether this residual transcript is translated, we performed additional western blot analysis using an antibody recognising the C-terminal region of Apc, which is absent following complete exon 15 recombination. Importantly, we did not detect full-length Wt-Apc protein in the severe *Cre:Apc*$^{LoxP15/15}$ model, whereas C-terminal Apc protein was clearly detectable in the *Cre:Apc*$^{LoxP1\text{-}15/15}$ model (**Appendix Figure S1e**). These data confirm that the milder phenotype retains low-level of Wt-Apc protein, consistent with a hypomorphic state.

Although the expression of Wt-Apc in the hypomorph mutants is low, many research articles have shown that small amounts of Wt-Apc are sufficient to be able to degrade β-catenin resulting in partial restoration of colon adenoma formation[9-11]. Studies using hypomorph allelic series showed that only 10% of Wt-Apc expression was sufficient to reduced tumour burden *in vivo* and *in vitro*[12]. Hence, we hypothesise like the published data in colon adenoma and other APC-driven tumours, that small amounts of Wt-Apc in the *Cre:Apc*$^{loxP1\text{-}15/15}$ leads to decreased tumour burden and less severe phenotype compared to the *Cre:Apc*$^{loxP15/15}$.

Comment 3: *The Sox2CreERT2 experiment shows early cluster formation, but the claim that Sox2⁺ cells are the cell of origin is stronger than the data*

Response: We thank the Reviewer for this important caution. We have revised the text throughout to avoid overstatement and now state that: "genetic deletion of *Apc* exon15 in Sox2+ve stem cell compartments can initiate tumour formation".

To further strengthen this conclusion, we have performed additional postnatal tamoxifen-induction experiments at (P4–P5), analysing pituitaries at 10 weeks of age (new **Appendix Figure S3**). These revealed anterior pituitary tumours characterised by whorl-like β-catenin-positive clusters, a hallmark of aCP pathology. Because the *Sox2*$^{CreERT2/+}$ drives expression in all Sox2 cell compartments, we could not leave these animals beyond the 10 weeks threshold, as they reach the end human point under our animal's experimentation licence. Together the new experiments show that deletion of *Apc* in both embryonic and postnatal Sox2+ve cells can initiate tumour formation.

Comment 4: *The distinction that Apc-driven clusters use p21 but not p16 is intriguing and potentially important. Evidence is limited to transcript levels. Should temper to:*
"These clusters display a transcriptional profile consistent with a SASP-like program".

Response: We agree that our initial interpretation required further validation beyond transcriptomic data. Since we believe this aspect of our research is very important, we have performed several new experiments to strengthen our previous results. Firstly, we provide double immunofluorescence (new **Appendix Figure S7**) of p21, p16 and the senescence markers: γ-H2AX (DNA-damage associated protein), NF-Κβ (regulator SASP that drives expression of pro-inflammatory cytokines) and the IL6-downstream signalling mediator pSTAT3 together with β-catenin. We show that clusters of accumulating β-catenin express high levels of p21, γ-H2AX, NF-Κβ and pSTAT3 in accordance with p21 mediated SASP whilst no expression of p16 was observed in the β-catenin+ve clusters. Together these results validate our mRNA-seq data and provides further evidence that p21-mediated SASP is activated in Apc-driven tumours.

Secondly, we have performed western blots to measure p21 levels (as a marker/read out of SASP) from tumours initiating cells (Lef1:GFP+ve cells) derived from tumours from our 3 different genotypes: *Cre:Apc*$^{LoxP15/15}$ (large severe embryonic tumours, **Fig.1**) , Cre:*Apc*$^{LoxP1\text{-}15/15}$ (milder aCP-postnatal tumours, **Fig.3-4-5**) and *Cre:Apc*$^{LoxP1\text{-}15/1\text{-}15}$ (only hyperplasia **Appendix Figure S1**) or Wt pituitaries. Western blot shows higher upregulation of p21 in tumour cells derived from our severe phenotype model (*Cre:Apc*$^{LoxP15/15}$) compared to lesser expression of p21 in the milder postnatal tumour genotype (*Cre:Apc*$^{LoxP1\text{-}15/15}$) or even less compared to

the *Cre:Apc$^{LoxP1-15/1-15}$* (only hyperplasia) (new **Fig. VE. 2 d-e**). Interestingly, we see a dose dependent increase of p21 which correlates with the tumour severity and the size of β-catenin accumulating cell clusters (new **Fig. VE. 2 a-c**). This data suggests a more robust p21-mediated SASP linked to tumour severity in the different mutants: *Cre:Apc$^{LoxP15/15}$, Cre:Apc$^{LoxP1-15/1-15}$* and *Cre:Apc$^{LoxP1-15/1-15}$*

Comment 5: "*The manuscript should more directly discuss prior work by Gonzalez-Meljem et al. (Nat Commun 2017), which included Apc knockout models in the context of pituitary tumourigenesis. In Gonzalez-Meljem's, while Apc loss activates Wnt/β-catenin signalling, the strength and paracrine impact of the resulting clusters differ from those driven by stabilized β-catenin in prior models and hence the authors conlcusen that Apc loss in their model is not tumorigeneic*"

Response: Thanks so much for this comment. We have included in the discussion the results from Gonzalez-Meljem et al. (Nat Commun 2017). Whilst these observations are very interesting and their model shows that genetic deletion of Apc exon14 does not lead to tumour formation due to small clusters of accumulating β-catenin that exhibit attenuated SASP, these results do not fully explain why patients with APC-mutations develop craniopharyngioma. We think that the results from Gonzalez-Meljem et al. (Nat Commun 2017) are not conflicting with our work, as their conclusion shows that attenuated senescence in their Apc-model using Hesx1Cre/+ line is not sufficient to drive tumour formation due to low SASP. In our models, we see different degrees of severity related to SASP robustness based on the levels of p21 and size of the β-catenin clusters (new **Fig. VE. 2**). This is in line with Gonzalez-Meljem et al. hypothesis. Our work presents two new models with larger clusters, high levels of p21 and sustained SASP sufficient to form tumours. The differences in phenotype between our models and previous published data may be due to several reasons: firstly, we use a different transgenic Cre, and different ApcLoxp lines to that published previously Gonzalez-Meljem et al. Prop1Cre line has been shown to be very efficient. Secondly, based on the literature we noticed that exon15 is a hotspot for mutations resulting in Apc-severe phenotypes, so we decided to use the ApcLoxP exon15/15. When we use LoxP sites covering the full coding sequence, ApcLoxP1-15/1-15, we did not achieve full recombination resulting in small clusters and low SASP as assessed by p21 expression (new **Fig. VE. 2**) leading to hyperplasia and not tumour formation. This could suggest that Gonzalez-Meljem's model is more like our mildest phenotype resulting only in hyperplasia with reduced clusters size and SASP which is not sufficient for tumorigenesis. By using different transgenic lines and deleting different exon, we achieve more robust senesce which is permissive of tumour formation with larger clusters of β-catenin.

Comment 6: "*explain what novel mechanistic or biological insights are contributed beyond these earlier observations.*"

Response: We thank the Reviewer for requesting clarification of the conceptual advance of our study. We have revised the discussion to more explicitly define these contributions.

Beyond prior work, our study provides the first evidence that:

1. Apc disruption is sufficient for the development of aCP tumorigenesis in vivo, resolving the prior assumption that Apc loss was non-tumorigenic in murine and human context.
2. We define a previously undescribed early tumour initiating cells specific transcriptional signature**.** We achieved this by purifying *Cre:Apc$^{LoxP15/15}$;Lef1:GFP+* tumour initiating cells (7 days post-deletion), capturing the earliest stages of transformation rather than end-stage tumour tissue.
3. Apc-driven tumour initiating cells undergo p21-driven SASP-program (p16-negative), defining a mechanistically distinct senescence state compared with previously described β-catenin-mutated+ve murine models.

4. Our data suggest that tumour outcome could be determined by Apc dosage and β-catenin+ve cluster size, suggesting a threshold mechanism in which residual Apc activity modulates β-catenin control and SASP intensity, thereby determining whether lesions progress from hyperplasia to tumour formation.
5. Our novel methylomic data comparing Apc-driven and β-catenin-driven murine tumours to publicly available human methylome data suggest a converge on shared epigenetic features across tumour typs, supporting a conserved epigenomic framework underlying tumour-associated inflammatory programming and SASP.

Collectively, these findings establish a **mechanistic model in which Apc disruption drives tumorigenesis leading to a p21-driven SASP state that is sufficient for tumour initiation in the pituitary**. Our data provides with novel biological insights and covers a current gap on aCP genetics. The findings have translational potential, as it broadens the APC-related phenotypic clinical spectrum to aCP tumours. It also hihglithg the importance to closer monitor patients with FAP or APC-related syndromes for the development of aCPs early in life.

REFERENCES:

1 Engel, N. W. *et al.* Canonical Wnt Signaling Drives Tumor-Like Lesions from Sox2-Positive Precursors of the Murine Olfactory Epithelium. *PLoS One* **11**, e0166690 (2016). https://doi.org/10.1371/journal.pone.0166690

2 Andoniadou, C. L. *et al.* Sox2(+) stem/progenitor cells in the adult mouse pituitary support organ homeostasis and have tumor-inducing potential. *Cell Stem Cell* **13**, 433-445 (2013). https://doi.org/10.1016/j.stem.2013.07.004

3 Chen, M. *et al.* Wnt-responsive Lgr5(+) globose basal cells function as multipotent olfactory epithelium progenitor cells. *J Neurosci* **34**, 8268-8276 (2014). https://doi.org/10.1523/JNEUROSCI.0240-14.2014

4 Yang, L. M., Huh, S. H. & Ornitz, D. M. FGF20-Expressing, Wnt-Responsive Olfactory Epithelial Progenitors Regulate Underlying Turbinate Growth to Optimize Surface Area. *Dev Cell* **46**, 564-580 e565 (2018). https://doi.org/10.1016/j.devcel.2018.07.010

5 Wang, Y. Z. *et al.* Canonical Wnt signaling promotes the proliferation and neurogenesis of peripheral olfactory stem cells during postnatal development and adult regeneration. *J Cell Sci* **124**, 1553-1563 (2011). https://doi.org/10.1242/jcs.080580

6 Capper, D. *et al.* DNA methylation-based classification of central nervous system tumours. *Nature* **555**, 469-474 (2018). https://doi.org/10.1038/nature26000

7 Marrero-Gutierrez, J. *et al.* Methylation and gene expression patterns in adamantinomatous craniopharyngioma highlight a panel of genes associated with disease progression-free survival. *Front Endocrinol (Lausanne)* **16**, 1585618 (2025). https://doi.org/10.3389/fendo.2025.1585618

8 Apps, J. R. *et al.* Recurrent adamantinomatous craniopharyngiomas show MAPK pathway activation, clonal evolution and rare TP53-loss-mediated malignant progression. *Acta Neuropathol Commun* **12**, 127 (2024). https://doi.org/10.1186/s40478-024-01838-4

9 Clarke, A. R. Wnt signalling in the mouse intestine. *Oncogene* **25**, 7512-7521 (2006). https://doi.org/10.1038/sj.onc.1210065

10 Buchert, M. *et al.* Genetic dissection of differential signaling threshold requirements for the Wnt/beta-catenin pathway in vivo. *PLoS Genet* **6**, e1000816 (2010). https://doi.org/10.1371/journal.pgen.1000816

11 Dow, L. E. *et al.* Apc Restoration Promotes Cellular Differentiation and Reestablishes Crypt Homeostasis in Colorectal Cancer. *Cell* **161**, 1539-1552 (2015). https://doi.org/10.1016/j.cell.2015.05.033
12 Li, Q., Ishikawa, T. O., Oshima, M. & Taketo, M. M. The threshold level of adenomatous polyposis coli protein for mouse intestinal tumorigenesis. *Cancer Res* **65**, 8622-8627 (2005). https://doi.org/10.1158/0008-5472.CAN-05-2145

19th Aug 2026

Dear Dr. Gaston-Massuet,

Thank you for the submission of your manuscript to EMBO Molecular Medicine. We have now received feedback from the two reviewers who agreed to evaluate your manuscript. As you will see from their reports pasted below both referees support publication of your manuscript. Therefore, I am pleased to inform you that we will be able to accept your manuscript pending the following final amendments:

1) Figures: We note that some images/panels are reused. Figure 5A image Cre:ApcLoxP1-15/15 α-pHH3 α-β-Catenin is reused in Figure EV2A. Please cite in the respective figure legend every reused image/panel.
2) Authors Checklist: Please complete column D.
3) Title: Please consider revising the title to better emphasize the main findings, e.g. "Apc-deficient pituitary stem cells drive adamantinomatous craniopharyngioma through senescence-associated inflammation"
4) In the main manuscript file, please do the following:
- Please address all comments suggested by our data editors listed below:
o Data availability statement
1. Please note that the specific URLs for datasets (GSE282683 (RNA) and GSE328638) are not provided in the data availability statement.
o Figure legends:
1. Please note that the legends for figure 2 are not provided in a sequential manner (legend for figure 2C is provided before legend of figure 2B). This needs to be rectified.
2. Please note that the exact p values are not provided in the legends of figures 3D,E; EV-2B,E; EV-3A.
3. Please indicate the statistical test used for data analysis in the legends of figure 7D.
4. Please note that the box plots need to be defined in terms of centre, minima, maxima, and whiskers, bounds of box and percentile in the legends of figure EV-3A,C.
5. Please note that information related to n is missing in the legends of figures 3D; 4C; EV-3A,C.
6. Please note that scale bar and its definition are missing for figure 1A.
- Please update the order and the headings of the manuscript sections to: Abstract / The Paper Explained / Introduction / Results / Discussion / Methods / Data Availability / Acknowledgements / Disclosure and Competing Interests Statement / References / Figure Legends / Expanded View Figure Legends
- Remove "data not shown" (p. 4).
- In Methods, a statistical paragraph should reflect all information that you have filled in the Authors Checklist, especially regarding randomization, blinding, replication.
- Indicate in legends exact n and exact p values, not a range, along with the statistical test used. To keep the figures "clear" some authors found providing an Appendix table Sx with all exact p-values preferable. You are welcome to do this if you want to.
- Rename "Declarations" to "Disclosure Statement & Competing Interests". We updated our journal's competing interests policy in January 2022 and request authors to consider both actual and perceived competing interests. Please review the policy https://www.embopress.org/competing-interests and update your competing interests if necessary.
- Author contributions: Please remove it from the manuscript and specify author contributions in our submission system. CRediT has replaced the traditional author contributions section because it offers a systematic machine-readable author contributions format that allows for more effective research assessment. You are encouraged to use the free text boxes beneath each contributing author's name to add specific details on the author's contribution. More information is available in our guide to authors:
https://www.embopress.org/page/journal/17574684/authorguide#authorshipguidelines
- Correct the reference citation in the text and reference list. In the text, a reference should be cited by author and year of publication. Include a space between a word and the opening parenthesis of the reference that follows. In the reference list, citations should be listed in alphabetical order. Where there are more than 10 authors on a paper, 10 will be listed, followed by "et al.". Please remove the DOIs for all published articles. Please check "Author Guidelines" for more information.
https://link.springer.com/journal/44321/submission-guidelines#cms-Reference-guidelines
5) Appendix: Please add page numbers to the table of content, move the figure legends underneath the corresponding figure and correct the nomenclature for the tables to "Appendix Table S1" and "Appendix Table S2". Also, update their callouts in the main manuscript.
6) Funding: Please make sure that information about all sources of funding are complete in both our submission system and in the manuscript. Parks-GOS(V4323), Barts Charity (MGU055), the Newton-Bhabha Fund and NIFR8\1037 are missing in our submission system. Please correct.
7) The Paper Explained: Please provide "The Paper Explained" and add it to the main manuscript text. Please check any of recently published EMBO Mol Med research articles for reference.
8) Synopsis: Every published paper now includes a 'Synopsis' to further enhance discoverability. Synopses are displayed on the journal webpage and are freely accessible to all readers. They include separate synopsis image and synopsis text.
- Synopsis image: Please provide a visual abstract as a high-resolution .jpeg file exactly 550 px-wide x 300 pixels high to illustrate your article.

- Synopsis text: Please provide a short standfirst (maximum of 300 characters, including space) as well as 2-5 one sentence bullet points that summarize the paper as a .doc file. Please write the bullet points to summarize the key NEW findings. They should be designed to be complementary to the abstract - i.e. not repeat the same text. We encourage inclusion of key acronyms and quantitative information (maximum of 30 words / bullet point). Please use the passive voice.
- Please check your synopsis text and image before submission with your revised manuscript. Please be aware that in the proof stage minor corrections only are allowed (e.g., typos).
9) As part of the EMBO Publications transparent editorial process (see our Editorial at http://embomolmed.embopress.org/content/2/9/329), EMBO Molecular Medicine will publish online a Review Process File (RPF) to accompany accepted manuscripts. This file will be published in conjunction with your paper and will include the anonymous referee reports, your point-by-point response and all pertinent correspondence relating to the manuscript. Let us know if you want to remove or not any figures from it prior to publication. Please note that the Authors checklist will be published at the end of the RPF.
10) Please provide a point-by-point letter INCLUDING my comments as well as the reviewer's reports and your detailed responses (as Word file).

I look forward to reading a new revised version of your manuscript as soon as possible.

Yours sincerely,

Zeljko Durdevic

Zeljko Durdevic
Senior Editor
EMBO Molecular Medicine

*** Instructions to submit your revised manuscript ***

*** PLEASE NOTE *** As part of the EMBO Publications transparent editorial process initiative (see our Editorial at https://www.embopress.org/doi/pdf/10.1002/emmm.201000094), EMBO Molecular Medicine will publish online a Review Process File to accompany accepted manuscripts.

In the event of acceptance, this file will be published in conjunction with your paper and will include the anonymous referee reports, your point-by-point response and all pertinent correspondence relating to the manuscript. If you do NOT want this file to be published, please inform the editorial office at contact@embomolmed.org.

*****

When preparing your revised manuscript, please refer to our guidelines: https://link.springer.com/journal/44321/submission-guidelines#cms-Revised-submissions. We perform an initial quality control of all revised manuscripts before re-review; failure to include requested items will delay the evaluation of your revision.

We require:

1) A .docx formatted version of the manuscript text (including legends for main figures, EV figures and tables). Please make sure that the changes are highlighted to be clearly visible.

2) Individual production quality figure files as .eps, .tif, .jpg (one file per figure). For guidance, download the 'Figure Guide PDF': https://media.springernature.com/original/springer-cms/rest/v1/content/27825798/data/v1.

3) A .docx formatted letter INCLUDING the reviewers' reports and your detailed point-by-point responses to their comments. As part of the EMBO Press transparent editorial process, the point-by-point response is part of the Review Process File (RPF), which will be published alongside your paper.

4) A complete author checklist, which you can download from our author guidelines. Please insert information in the checklist

that is also reflected in the manuscript. The completed author checklist will also be part of the RPF.

5) Please note that all corresponding authors are required to supply an ORCID ID for their name upon submission of a revised manuscript.

6) It is mandatory to include a 'Data Availability' section after the Materials and Methods. Before submitting your revision, primary datasets produced in this study need to be deposited in an appropriate public database, and the accession numbers and database listed under 'Data Availability'. Please remember to provide a reviewer password if the datasets are not yet public.

In case you have no data that requires deposition in a public database, please state so in this section. Note that the Data Availability Section is restricted to new primary data that are part of this study.

7) For data quantification: please specify the name of the statistical test used to generate error bars and P values, the number (n) of independent experiments (specify technical or biological replicates) underlying each data point and the test used to calculate p-values in each figure legend. The figure legends should contain a basic description of n, P and the test applied. Graphs must include a description of the bars and the error bars (s.d., s.e.m.).

8) At EMBO Press we ask authors to provide source data for the main manuscript figures. You will receive a separate email with instructions for providing source data with your revised manuscript, including how to upload and organize the files.

9) Our journal encourages inclusion of *data citations in the reference list* to directly cite datasets that were re-used and obtained from public databases. Data citations in the article text are distinct from normal bibliographical citations and should directly link to the database records from which the data can be accessed. In the main text, data citations are formatted as follows: "Data ref: Smith et al, 2001" or "Data ref: NCBI Sequence Read Archive PRJNA342805, 2017". In the Reference list, data citations must be labeled with "[DATASET]". A data reference must provide the database name, accession number/identifiers and a resolvable link to the landing page from which the data can be accessed at the end of the reference.

10) We replaced Supplementary Information with Expanded View (EV) Figures and Tables that are collapsible/expandable online. A maximum of 5 EV Figures can be typeset. EV Figures should be cited as 'Figure EV1, Figure EV2" etc... in the text and their respective legends should be included in the main text after the legends of regular figures.

- For the figures that you do NOT wish to display as Expanded View figures, they should be bundled together with their legends in a single PDF file called *Appendix*, which should start with a short Table of Content. Appendix figures should be referred to in the main text as: "Appendix Figure S1, Appendix Figure S2" etc.

- Additional Tables/Datasets should be labeled and referred to as Table EV1, Dataset EV1, etc. Legends have to be provided in a separate tab in case of .xls files. Alternatively, the legend can be supplied as a separate text file (README) and zipped together with the Table/Dataset file.

11) The paper explained: EMBO Molecular Medicine articles are accompanied by a summary of the articles to emphasize the major findings in the paper and their medical implications for the non-specialist reader. Please provide a draft summary of your article highlighting

- the medical issue you are addressing,

- the results obtained and

- their clinical impact.

This may be edited to ensure that readers understand the significance and context of the research. Please refer to any of our published articles for an example.

12) Author contributions: You will be asked to provide CRediT (Contributor Role Taxonomy) terms in the submission system. These replace a narrative author contribution section in the manuscript.

13) A Conflict of Interest statement should be provided in the main text.

14) Every published paper includes a 'Synopsis' to further enhance discoverability. Synopses are displayed on the journal webpage and are freely accessible to all readers. They include a short stand first (maximum of 300 characters, including space) as well as 2-5 one-sentences bullet points that summarizes the paper. Please write the bullet points to summarize the key NEW findings. They should be designed to be complementary to the abstract - i.e. not repeat the same text. We encourage inclusion of key acronyms and quantitative information (maximum of 30 words / bullet point). Please use the passive voice. Please attach these in a separate file or send them by email, we will incorporate them accordingly.

Please also suggest a striking image or visual abstract to illustrate your article as a PNG file 550 px wide x 300 px high.

15) Include a Reagents and Tools Table as part of the Methods section, which can be downloaded from our author guidelines.

EMBO Molecular Medicine has a "scooping protection" policy, whereby similar findings that are published by others during review or revision are not a criterion for rejection. Should you decide to submit a revised version, I do ask that you get in touch after three months if you have not completed it, to update us on the status.

*Additional important information regarding Figures

Each figure should be given in a separate file and should have the following resolution:
Graphs 800-1,200 DPI
Photos 400-800 DPI
Colour (only CMYK) 300-400 DPI"

Figures are not edited by the production team. All lettering should be the same size and style; figure panels should be indicated by capital letters (A, B, C etc). Gridlines are not allowed except for log plots. Figures should be numbered in the order of their appearance in the text with Arabic numerals. Each Figure must have a separate legend and a caption is needed for each panel.

*Additional important information regarding figures and illustrations can be found at
https://media.springernature.com/original/springer-cms/rest/v1/content/27825798/data/v1

***** Reviewer's comments *****

Referee #1 (Comments on Novelty/Model System for Author):

All fine

Referee #1 (Remarks for Author):

Thanks for addressing my comments

Referee #2 (Remarks for Author):
Is suitable for publication

The authors addressed the remaining editorial issues.

8th Sep 2026

Dear Dr. Gaston-Massuet,

We are pleased to inform you that your manuscript is accepted for publication and is now being sent to our publisher to be included in the next available issue of EMBO Molecular Medicine.

Your manuscript will be processed for publication by EMBO Press. It will be copy edited and you will receive page proofs prior to publication. Please note that you will be contacted by Springer Nature Author Services to complete licensing and payment information.

You may qualify for financial assistance for your publication charges - either via a Springer Nature fully open access agreement or an EMBO initiative. Check your eligibility: https://link.springer.com/journal/44321/how-to-publish-with-us

Should you be planning a Press Release on your article, please notify the production team via this form in order to coordinate publication and release dates: https://forms.cloud.microsoft/e/eH4A8JVP6g

If you have any questions, please do not hesitate to contact the Editorial Office. Thank you for your contribution to EMBO Molecular Medicine.

Yours sincerely,
Zeljko Durdevic

Zeljko Durdevic
Senior Editor
EMBO Molecular Medicine

>>> Please note that it is EMBO Molecular Medicine policy for the transcript of the editorial process (containing referee reports and your response letter) to be published as an online supplement to each paper. If you do NOT want this, you will need to inform the Editorial Office via email immediately. More information is available here: https://link.springer.com/partners/embo-press/editorial-policies#Peer%20review

EMBO Press Author Checklist

Corresponding Author Name: Carles Gaston-Massuet	
Journal Submitted to: Embo Molecular Medicine	
Manuscript Number: EMM-2025-23130-V2	

USEFUL LINKS FOR COMPLETING THIS FORM
The EMBO Journal - Author Guidelines
EMBO Reports - Author Guidelines
Molecular Systems Biology - Author Guidelines
EMBO Molecular Medicine - Author Guidelines

Reporting Checklist for Life Science Articles (updated January
This checklist is adapted from Materials Design Analysis Reporting (MDAR) Checklist for Authors. MDAR establishes a minimum set of requirements in transparent reporting in the life sciences (see Statement of Task: 10.31222/osf.io/9sm4x). Please follow the journal's guidelines in preparing your
Please note that a copy of this checklist will be published alongside your article.

Abridged guidelines for figures
1. Data
The data shown in figures should satisfy the following conditions:
- the data were obtained and processed according to the field's best practice and are presented to reflect the results of the experiments in an accurate and unbiased manner.
- ideally, figure panels should include only measurements that are directly comparable to each other and obtained with the same assay.
- plots include clearly labeled error bars for independent experiments and sample sizes. Unless justified, error bars should not be shown for technical
- if n<5, the individual data points from each experiment should be plotted. Any statistical test employed should be justified.
- Source Data should be included to report the data underlying figures according to the guidelines set out in the authorship guidelines on Data

2. Captions
Each figure caption should contain the following information, for each panel where they are relevant:
- a specification of the experimental system investigated (eg cell line, species name).
- the assay(s) and method(s) used to carry out the reported observations and measurements.
- an explicit mention of the biological and chemical entity(ies) that are being measured.
- an explicit mention of the biological and chemical entity(ies) that are altered/varied/perturbed in a controlled manner.
- the exact sample size (n) for each experimental group/condition, given as a number, not a range;
- a description of the sample collection allowing the reader to understand whether the samples represent technical or biological replicates (including how many animals, litters, cultures, etc.).
- a statement of how many times the experiment shown was independently replicated in the laboratory.
- definitions of statistical methods and measures:
  - common tests, such as t-test (please specify whether paired vs. unpaired), simple χ2 tests, Wilcoxon and Mann-Whitney tests, can be unambiguously identified by name only, but more complex techniques should be described in the methods section;
  - are tests one-sided or two-sided?
  - are there adjustments for multiple comparisons?
  - exact statistical test results, e.g., P values = x but not P values < x;
  - definition of 'center values' as median or average;
  - definition of error bars as s.d. or s.e.m.

Please complete ALL of the questions below.
Select "Not Applicable" only when the requested information is not relevant for your study.

Materials

**Newly Created Materials**	**Information included in the manuscript?**	**In which section is the information available?** (Reagents and Tools Table, Materials and Methods, Figures, Data Availability Section)
New materials and reagents need to be available; do any restrictions apply?	Yes	Materials and methods and supplementary materials and methods

**Antibodies**	**Information included in the manuscript?**	**In which section is the information available?** (Reagents and Tools Table, Materials and Methods, Figures, Data Availability Section)
For **antibodies** provide the following information:<br>- Commercial antibodies: RRID (if possible) or supplier name, catalogue number and or/clone number<br>- Non-commercial: RRID or citation	Yes	Supplementary Table 1

**DNA and RNA sequences**	**Information included in the manuscript?**	**In which section is the information available?** (Reagents and Tools Table, Materials and Methods, Figures, Data Availability Section)
**Short novel DNA or RNA including primers, probes**: provide the sequences.	Yes	Supplementary Table 2

**Cell materials**	**Information included in the manuscript?**	**In which section is the information available?** (Reagents and Tools Table, Materials and Methods, Figures, Data Availability Section)
**Cell lines:** Provide species information, strain. Provide accession number in repository **OR** supplier name, catalog number, clone number, and/**OR** RRID.	Not Applicable	NA
**Primary cultures:** Provide species, strain, sex of origin, genetic modification status.	Not Applicable	NA
Report if the cell lines were recently **authenticated** (e.g., by STR profiling) and tested for mycoplasma contamination.	Not Applicable	NA

**Experimental animals**	**Information included in the manuscript?**	**In which section is the information available?** (Reagents and Tools Table, Materials and Methods, Figures, Data Availability Section)
**Laboratory animals or Model organisms:** Provide species, strain, sex, age, genetic modification status. Provide accession number in repository **OR** supplier name, catalog number, clone number, **OR** RRID.	Yes	This is provided in materials and methods section under animals
**Animal observed in or captured from the field:** Provide species, sex, and age where possible.	Not Applicable	NA
Please detail **housing and husbandry conditions**.	Yes	This is provided in materials and methods section under animals

**Plants and microbes**	**Information included in the manuscript?**	**In which section is the information available?** (Reagents and Tools Table, Materials and Methods, Figures, Data Availability Section)
**Plants:** provide species and strain, ecotype and cultivar where relevant, unique accession number if available, and source (including location for collected wild specimens).	Not Applicable	NA
**Microbes:** provide species and strain, unique accession number if available, and source.	Not Applicable	NA

**Human research participants**	**Information included in the manuscript?**	**In which section is the information available?** (Reagents and Tools Table, Materials and Methods, Figures, Data Availability Section)
If collected and within the bounds of privacy constraints report on age, sex and gender or ethnicity for all study participants.	Not Applicable	NA

**Core facilities**	**Information included in the manuscript?**	**In which section is the information available?** (Reagents and Tools Table, Materials and Methods, Figures, Data Availability Section)
If your work benefited from core facilities, was their service mentioned in the acknowledgments section?	Yes	UCL genomics ICH in the materials and methods sections DNA methylation analyses

Design

Study protocol	Information included in the manuscript?	In which section is the information available? (Reagents and Tools Table, Materials and Methods, Figures, Data Availability Section)
If study protocol has been **pre-registered, provide DOI in the manuscript**. For clinical trials, provide the trial registration number **OR** cite DOI.	Not Applicable	NA
Report the **clinical trial registration number** (at ClinicalTrials.gov or equivalent), where applicable.	Not Applicable	NA

Laboratory protocol	Information included in the manuscript?	In which section is the information available? (Reagents and Tools Table, Materials and Methods, Figures, Data Availability Section)
Provide DOI OR other citation details if **external detailed step-by-step protocols** are available.	Yes	Materials and Methods

Experimental study design and statistics	Information included in the manuscript?	In which section is the information available? (Reagents and Tools Table, Materials and Methods, Figures, Data Availability Section)
Include a statement about **sample size** estimate even if no statistical methods were used.	Yes	Materials and Methods, Figures and Data Availability
Were any steps taken to minimize the effects of subjective bias when allocating animals/samples to treatment (e.g. **randomization procedure**)? If yes, have they been described?	Yes	Materials and Methods: under Animals
Include a statement about **blinding** even if no blinding was done.	Yes	Materials and Methods: under Animals
Describe **inclusion/exclusion criteria** if samples or animals were excluded from the analysis. Were the criteria pre-established?<br><br>If sample or data points were omitted from analysis, report if this was due to attrition or intentional exclusion and provide justification.	Not Applicable	No animal were excluded from the study and no data was omitted
For every figure, are **statistical tests** justified as appropriate? Do the data meet the assumptions of the tests (e.g., normal distribution)? Describe any methods used to assess it. Is there an estimate of variation within each group of data? Is the variance similar between the groups that are being statistically compared?	Yes	Each figure has the statistical test

Sample definition and in-laboratory replication	Information included in the manuscript?	In which section is the information available? (Reagents and Tools Table, Materials and Methods, Figures, Data Availability Section)
In the figure legends: state number of times the experiment was **replicated** in laboratory.	Yes	Figures
In the figure legends: define whether data describe **technical or biological replicates**.	Yes	Figures

Ethics

Ethics	Information included in the manuscript?	In which section is the information available? (Reagents and Tools Table, Materials and Methods, Figures, Data Availability Section)
Studies involving **human participants**: State details of **authority granting ethics approval** (IRB or equivalent committee(s), provide reference number for approval.	Not Applicable	NA
Studies involving **human participants**: Include a statement confirming that **informed consent** was obtained from all subjects and that the experiments conformed to the principles set out in the WMA Declaration of Helsinki and the Department of Health and Human Services Belmont Report.	Not Applicable	NA
Studies involving **human participants:** For publication of **patient photos**, include a statement confirming that consent to publish was obtained.	Not Applicable	NA
Studies involving experimental **animals**: State details of **authority granting ethics approval** (IRB or equivalent committee(s), provide reference number for approval. Include a statement of compliance with ethical regulations.	Yes	This is explained in Matherials and Methods
Studies involving **specimen and field samples:** State if relevant **permits** obtained, provide details of authority approving study; if none were required, explain why.	Not Applicable	NA

Dual Use Research of Concern (DURC)	Information included in the manuscript?	In which section is the information available? (Reagents and Tools Table, Materials and Methods, Figures, Data Availability Section)
Could your study fall under dual use research restrictions? Please check biosecurity documents and list of **select agents and toxins** (CDC): https://www.selectagents.gov/sat/list.htm	Not Applicable	NA
If you used a select agent, is the security level of the lab appropriate and reported in the manuscript?	Not Applicable	NA
If a study is subject to dual use research of concern regulations, is the name of the **authority granting approval and reference number** for the regulatory approval provided in the manuscript?	Not Applicable	NA

Reporting

The MDAR framework recommends adoption of discipline-specific guidelines, established and endorsed through community initiatives. Journals have their own policy about requiring specific guidelines and recommendations to complement MDAR.

Adherence to community standards	Information included in the manuscript?	In which section is the information available? (Reagents and Tools Table, Materials and Methods, Figures, Data Availability Section)
State if relevant guidelines or checklists (e.g., **ICMJE, MIBBI, ARRIVE, PRISMA**) have been followed or provided.	Not Applicable	NA
For **tumor marker prognostic studies,** we recommend that you follow the **REMARK** reporting guidelines (see link list at top right). See author guidelines, under 'Reporting Guidelines'. Please confirm you have followed these guidelines.	Not Applicable	NA
For **phase II and III randomized controlled trials**, please refer to the **CONSORT** flow diagram (see link list at top right) and submit the CONSORT checklist (see link list at top right) with your submission. See author guidelines, under 'Reporting Guidelines'. Please confirm you have submitted this list.	Not Applicable	NA

Data Availability

Data availability	Information included in the manuscript?	In which section is the information available? (Reagents and Tools Table, Materials and Methods, Figures, Data Availability Section)
Have **primary datasets** been deposited according to the journal's guidelines (see 'Data Deposition' section) and the respective accession numbers provided in the Data Availability Section?	Yes	Yes in matherials and methods & data availabitiy section
Were **human clinical and genomic datasets** deposited in a public access-controlled repository in accordance to ethical obligations to the patients and to the applicable consent agreement?	Yes	Yes in matherials and methods & data availabitiy section
Are **computational models** that are central and integral to a study available without restrictions in a machine-readable form? Were the relevant accession numbers or links provided?	Not Applicable	NA
If publicly available data were reused, provide the respective **data citations in the reference list**.	Yes	Yes this has been done in matherial and methods
