# Supplementary material for: Apc-deficient pituitary stem cells drive adamantinomatous craniopharyngioma through senescence-associated inflammation
Source: EMBO Mol Med. 2026 Oct 9;18(11):4386–412. doi: 10.1038/s44321-026-00525-2 (PMC13748607; doi:10.1038/s44321-026-00525-2)

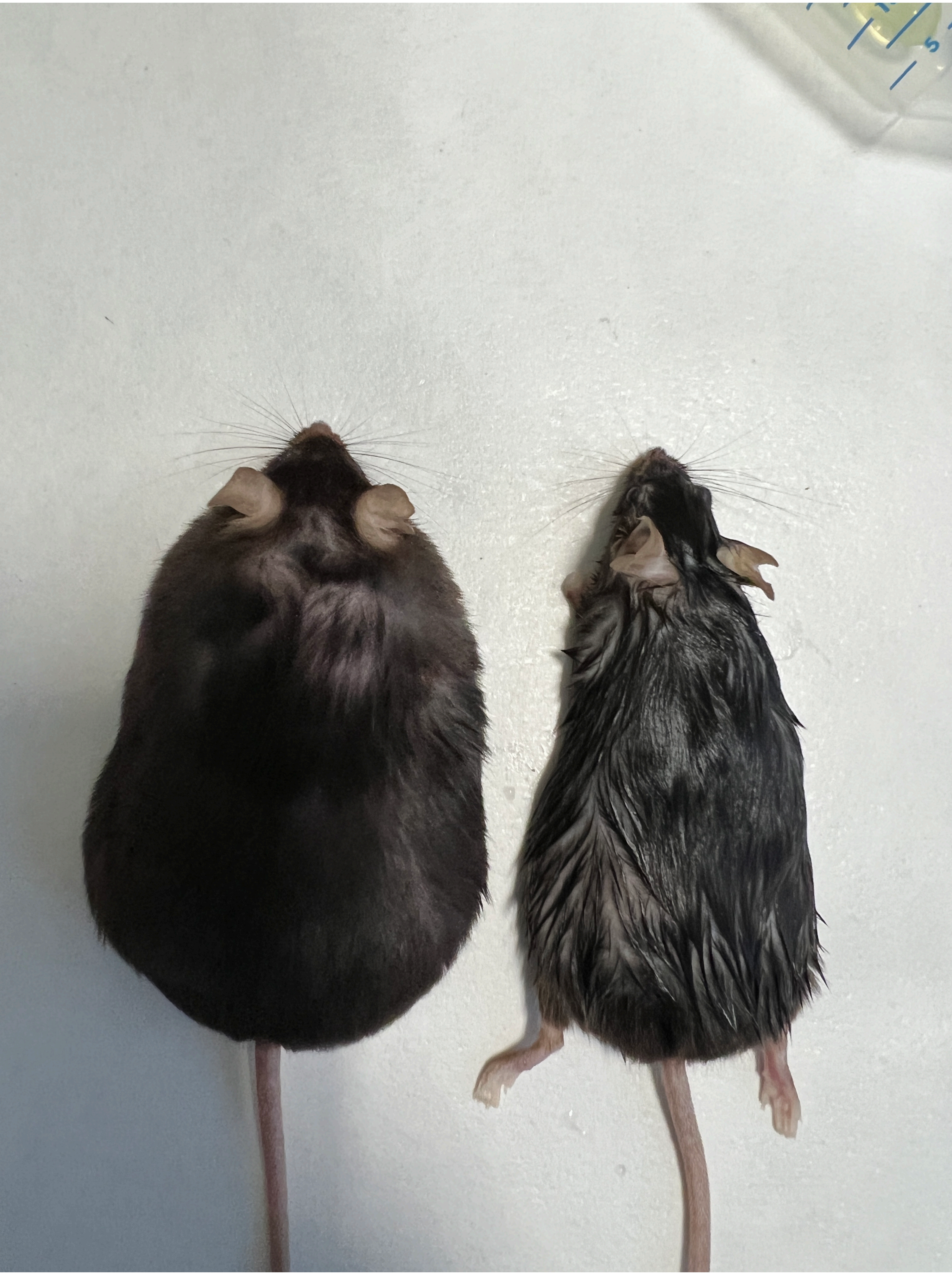

Supplement: Supplementary file 6 [file 44321_2026_525_MOESM5_ESM.zip › Figure 3/Fig. 3c/IMG_9425.JPG.pdf]
